# The genome-scale metabolic network analysis *of Zymomonas mobilis *ZM4 explains physiological features and suggests ethanol and succinic acid production strategies

**DOI:** 10.1186/1475-2859-9-94

**Published:** 2010-11-24

**Authors:** Kyung Yun Lee, Jong Myoung Park, Tae Yong Kim, Hongseok Yun, Sang Yup Lee

**Affiliations:** 1 Metabolic and Biomolecular Engineering National Research Laboratory, Department of Chemical and Biomolecular Engineering (BK21 program), KAIST, 335 Gwahangno, Yuseong-gu, Daejeon 305-701, Republic of Korea; 2 BioProcess Engineering Research Center, and Center for Systems and Synthetic Biotechnology, Institute for the BioCentury, KAIST, 335 Gwahangno, Yuseong-gu, Daejeon 305-701, Republic of Korea; 3 Bioinformatics Research Center, KAIST, 335 Gwahangno, Yuseong-gu, Daejeon 305-701, Republic of Korea

## Abstract

**Background:**

*Zymomonas mobilis *ZM4 is a Gram-negative bacterium that can efficiently produce ethanol from various carbon substrates, including glucose, fructose, and sucrose, *via *the Entner-Doudoroff pathway. However, systems metabolic engineering is required to further enhance its metabolic performance for industrial application. As an important step towards this goal, the genome-scale metabolic model of *Z. mobilis *is required to systematically analyze *in silico *the metabolic characteristics of this bacterium under a wide range of genotypic and environmental conditions.

**Results:**

The genome-scale metabolic model of *Z. mobilis *ZM4, ZmoMBEL601, was reconstructed based on its annotated genes, literature, physiological and biochemical databases. The metabolic model comprises 579 metabolites and 601 metabolic reactions (571 biochemical conversion and 30 transport reactions), built upon extensive search of existing knowledge. Physiological features of *Z. mobilis *were then examined using constraints-based flux analysis in detail as follows. First, the physiological changes of *Z. mobilis *as it shifts from anaerobic to aerobic environments (i.e. aerobic shift) were investigated. Then the intensities of flux-sum, which is the cluster of either all ingoing or outgoing fluxes through a metabolite, and the maximum *in silico *yields of ethanol for *Z. mobilis *and *Escherichia coli *were compared and analyzed. Furthermore, the substrate utilization range of *Z. mobilis *was expanded to include pentose sugar metabolism by introducing metabolic pathways to allow *Z. mobilis *to utilize pentose sugars. Finally, double gene knock-out simulations were performed to design a strategy for efficiently producing succinic acid as another example of application of the genome-scale metabolic model of *Z. mobilis*.

**Conclusion:**

The genome-scale metabolic model reconstructed in this study was able to successfully represent the metabolic characteristics of *Z. mobilis *under various conditions as validated by experiments and literature information. This reconstructed metabolic model will allow better understanding of *Z. mobilis *metabolism and consequently designing metabolic engineering strategies for various biotechnological applications.

## Background

The impact of biotechnology on industry and society is dramatically gaining momentum, particularly in the field of agriculture-food, medicine and chemical production. For the chemical industry, which aims to producing value-added chemicals and fuels in a sustainable way, efforts have been put into strain improvement of microorganisms, utilizing many newly emerging state-of-art techniques, for the overproduction of chemicals of interest [1-6]. Yet, the most common problem encountered in strain improvement is that the microorganisms are not naturally optimized for the overproduction of the target compounds desired for human use. Instead, these compounds are produced in small amounts which are sufficient for the microorganism's purpose. Therefore, it is necessary to engineer the microorganism so that the metabolic fluxes are redirected towards overproducing the target products without significantly hampering the overall cellular behavior.

*In silico *genome-scale metabolic modeling and simulation have proven to be useful in the field of systems metabolic engineering. This approach has successfully contributed to the design of strategies for engineering microorganisms for the production of amino acids, including L-valine [7] and L-threonine [8], lycopene [9], succinic acid [10], ethanol [11], and polylactic acid [12]. Genome-scale modeling and constraints-based flux analysis enables the calculation of intracellular fluxes based on the complex stoichiometric relationship of metabolites constituting the metabolic network. The strength of genome-scale modeling is that it not only predicts the effects of genetic and environmental perturbations on cellular metabolism from a holistic point of view, but can also be used in combination with other high-throughput techniques, for instance gene expression data [7,13].

*Zymomonas mobilis*, a Gram-negative bacterium, metabolizes glucose, fructose and sucrose *via *the Entner-Doudoroff (ED) pathway, and is capable of producing up to 12% (w/v) ethanol at a faster rate than yeast [14,15]. In addition to its high ethanol producing ability, its fast sugar consumption and processing rate, and high ethanol tolerance of up to 16% (vol/vol) have attracted attention to *Z. mobilis*, as a host for industrial biotechnology [15,16]. Recently, Seo *et al*. [15] first reported the complete sequence and the annotation of the *Z. mobilis *ZM4 genome, and Yang *et al*. [17] updated the data, enabling subsequent systematic studies of this organism and hence applications. Spurred with this complete genome sequence and annotation of *Z. mobilis *ZM4 genome, its genome-scale modeling and simulation could be employed for systematic analyses to understand the characteristics of its metabolism and to design efficient metabolic strategies.

**Figure 1:**
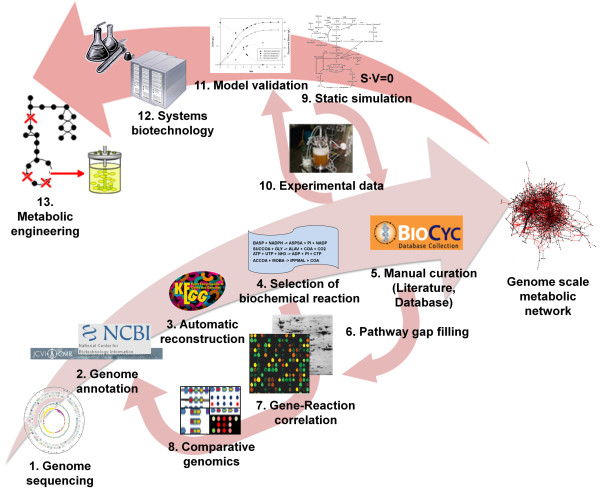
**Procedure for the reconstruction of genome-scale metabolic network in *Z. mobilis *and its application to metabolic engineering**. Automatic reconstruction of metabolic network based on genome sequence and annotation data (1-2-3-4). Manual curation of the metabolic network using literatures, databases, gene-reaction correlation, and comparative genomics to correct errors and fill gaps in the pathways (4-7-8-2 or 5-6-8-2). Determination of biomass composition and validation of metabolic model in comparison with experimental data (9-10-11). Systems metabolic engineering for strain improvement by combining experimental and *in silico *approaches (12-13). The detailed information about fermentation profile (11), utilized to refine reconstructed metabolic model is in additional file 7.

A small scale model of engineered *Z. mobilis *has already been constructed by Tsantili *et al*. [18]. This small scale model consists of the central metabolism supported by a few key metabolic reactions that are a lumped representation of cellular functions. However, with the genome-scale metabolic model, we aim to describe the overall metabolic characteristics of *Z. mobilis *with greater accuracy and scope of its metabolic functions. Pinto *et al*. [19] reported a study about data integration process for the metabolic network reconstruction of the *Z. mobilis*. Pinto *et al *took genome annotation data about *Z. mobilis *from the NCBI, and obtained the reaction list with stoichiometry data from KEGG [20], BioCyc [21] and BRENDA [22]. However, Pinto *et al *only focused on the first steps for the collection and processing of the information related to the reconstruction of genome-scale metabolic network. For the reconstruction of promising genome-scale metabolic model, several steps such as proofreading process, determination of biomass composition, and *in silico *simulations and their validations of the model based on experiments should be encompassed, as depicted on Figure 1. Here, we present a genome-scale metabolic model of the *Z. mobilis *ZM4, ZmoMBEL601, composed of 601 reactions and 579 metabolites, for systematic characterization of this organism (Table 1; Additional file 1 and 2).

**Table 1:** Features of the *in silico *metabolic model of *Z. mobilis *ZM4

Features	Number
Genome feature^a^	
Genome size (base pair, bp)	2,056,363
Total genes	1,808
Open reading frames (ORFs)	1,728
*In silico *metabolic model	
Total reactions	601
Biochemical reactions	571
Transport reactions	30
Metabolites	579
ORFs assigned in metabolic network	348
ORF coverage^b^	20.14%

^a^The genome features of *Z. mobilis *ZM4 were obtained from NCBI [15,17,23].

^b^The number of ORFs incorporated in the genome-scale metabolic model divided by the total number of ORFs in the genome of *Z. mobilis.*

## Results and discussion

### Genome-scale reconstruction of *Z. mobilis *ZM4 metabolic model

To reconstruct the *Z. mobilis *metabolic model, the NCBI [23], CMR [24], and ExPASy [25] databases are utilized first to obtain information regarding *Z. mobilis*'s genome sequence and its annotation. Then, the data on the metabolic reactions and metabolites were obtained from several databases and literatures. KEGG [20], which contains diverse information about biological pathways, was predominantly utilized in the construction of the draft metabolic model (Figure 1). The TCDB [26] and TransportDB [27] were employed in the collection of information regarding transport systems, which KEGG is deficient in, present in *Z. mobilis *to implement the nutrient uptake and product secretion systems between the intracellular and external environments. Meanwhile, the BioCyc [21] database was used to define each reaction's reversibility. Additionally, the BioSilico database [28] was applied to compare and integrate previously obtained information. Reactions, which are not assigned to a gene in *Z. mobilis *or for which no evidence is available for its presence in *Z. mobilis*, can also be added to the metabolic model. In the former case, literature evidence would allow for the addition of the metabolic reaction to the metabolic model. In the latter case, there are instances where the metabolic reaction where no literature evidence is present but is necessary to achieve the feasible flux distribution in the reconstructed metabolic model. In this case the metabolic reaction is added and noted as a point of interest in further studies into *Z. mobilis*.

The resulting ZmoMBEL601 metabolic model comprises 579 metabolites and 601 metabolic reactions, comprising of 571 biochemical conversions and 30 transport reactions. A total of 347 open reading frames (ORFs) were included in the metabolic model, which represents approximately 20.1% of the ORFs with assigned function in the *Z. mobilis *ZM4 genome (Table 1; Additional file 1 and 2). This ORF coverage in the ZmoMBEL601 metabolic model is similar to other reported genome-scale metabolic models (Additional file 3) [29-38] due to its small genome size. Once reconstructed, ZmoMBEL601 was dissected in detail to further characterize the metabolic network.

### General features of the *Z. mobilis *ZM4 metabolic model

**Figure 2:**
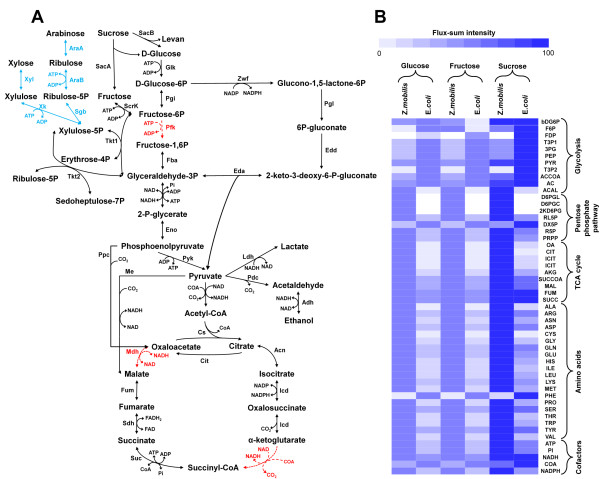
**Central metabolism and analysis of flux-sum intensity in *Z. mobilis***. (A) Central metabolism, including ED Pathway and TCA cycle in *Z. mobilis *based on annotated genes. Reactions for utilizing pentose sugars in *Z. mobilis *are introduced and represented to blue lines. The red lines indicate the missing reactions in *Z. mobilis*. (B) Flux-sum intensity of selected metabolites, including cofactors, amino acids and others, in central metabolism for *Z. mobilis *and *E. coli*, respectively, for three carbon sources (i.e. glucose, fructose, and sucrose). Uptake rate of each carbon source is fixed to 10 mmol/gDCW/h, and reaction for oxygen uptaking was deleted to describe anaerobic condition in both organisms. NGAME (i.e. non-growth associated maintenance energy) value for both organisms was eliminated. Upper constraints (i.e. limit the flux on fumaric acid, acetic acid, malic acid, acetoin, and acetaldehyde production) in *Z. mobilis *metabolic model for regulating the flux more realistically are relieved. Results are normalized by maximum value of each metabolite. Darker color indicates stronger flux-sum intensity.

The central carbon metabolism of *Z. mobilis *is different compared to other known gram-negative microorganisms, such as *E. coli*. *Z. mobilis *is known to metabolize only glucose, fructose and sucrose through the ED pathway, producing ethanol and CO_2_, and is unable to utilize the glycolytic pathway due to the absence of 6-phosphofructokinase, which converts fructose-6-phosphate into fructose-1,6-bisphosphate (Figure 2A) [16,39]. *Z. mobilis *ZM4 also does not have two enzymes in the tricarboxylic acid (TCA) cycle: 2-oxoglutarate dehydrogenase and malate dehydrogenase (Figure 2A). Despite the absence of these enzymes, *Z. mobilis *is still able to produce important building blocks including oxaloacetate, malic acid, and fumaric acid through alternative metabolic pathways; phosphoenolpyruvate carboxylase (phophoenolpyruvate + CO_2 _→ oxaloacetate + orthophosphate) and citrate lyase (citrate → acetate + oxaloacetate) for oxaloacetate production, malic enzyme (malate ↔ pyruvate + CO_2_) for malic acid production, and fumarate dehydratase (malate ↔ fumarate) for fumaric acid production. These characteristic features of central metabolism in *Z. mobilis *ZM4 were reflected in ZmoMBEL601.

The equation for biomass formation, which requires precursors from a set of biosynthetic reactions, was constructed to describe the cell growth (Additional file 1 and 4). The lipid composition of *Z. mobilis *was found to have a unique feature compared to other organisms, which allows for tolerance to higher alcohol levels. Because short-chain alcohols (e.g. methanol, ethanol) directly interacts with the lipid bilayer and increases its fluidity, the cell tries to maintain its membrane fluidity by changing the composition of the lipid layer; increasing the amount of long-chain fatty acids to create a rigid cell wall and synthesizing hopanoids, a pentacyclic lipid compounds, which can adjust the cell membrane permeability. This abundance of hopanoids and vaccenic acid (C18:1) in *Z. mobilis *was proposed to explain the evolutionary adaptation of *Z. mobilis *to survive in the presence of high ethanol level [40]. It has been reported that the lipids of *Z. mobilis *are composed of three groups of compounds: phospholipids, hopanoids, and nonpolar lipids [41]. Hopanoids are further categorized into five types: tetrahydroxybacteriohopanetetrol (THBH), tetrahydroxybacteriohopane-glucosamine (THBH-GA), tetrahydroxybacteriohopane-ether (THBH-ET), diplopterol, and dopene [41-43]. However, genes involved in the biosynthesis of the five types of hopanoids have not been annotated. Therefore, hopanoids biosynthetic pathways were constructed and incorporated into the metabolic model based on the results previously reported [42,44-46] (Additional file 1 and 4). Reactions biosynthesizing 5 different types of phospholipids [40,41,47] and 9 different types of nonpolar lipids [40] in the membrane of *Z. mobilis *were included in the same manner based on existing reports.

### Physiological characteristics of *Z. mobilis *related to aerobic metabolism

*Z. mobilis *ZM4 is a facultative anaerobic microorganism, and has been reported to have a reduced growth rate and ethanol production rate under aerobic conditions compared to anaerobic condition [15]. This phenomenon can be explained by the presence of two key enzymes of the *Z. mobilis *metabolism; a type 2 NADH oxidoreductase and an NADH oxidase.

*Z. mobilis *possesses a type 2 NADH oxidoreductase, which does not pump protons during electron transport in aerobic respiration, unlike the more common type 1 NADH oxidoreductase [48]. Thus, type 2 NADH oxidoreductase does not contribute to the proton gradient of the cellular membrane, which is the driving force in generating ATP. While this type 2 NADH oxidoreductase does not pump protons while passing electrons through the electron transport chain in *Z. mobilis*, other membrane proteins, such as cytochrome bc1 complex, electron transfer flavoprotein, and ubiquinone protein, are present to generate the proton gradient and thereby drive ATP generation under aerobic condition. Therefore, *Z. mobilis *can grow under aerobic conditions but at a lower growth rate due to the decreased supply of ATP. The *Z. mobilis *NADH oxidase catalyzes the oxidization of NADH (NADH + 0.5 O_2 _→ NAD) under aerobic condition. Because of this enzyme, the pool of NADH, which is used for ethanol production, is decreased, resulting in a decrease in capacity for producing ethanol under aerobic condition [48].

**Figure 3:**
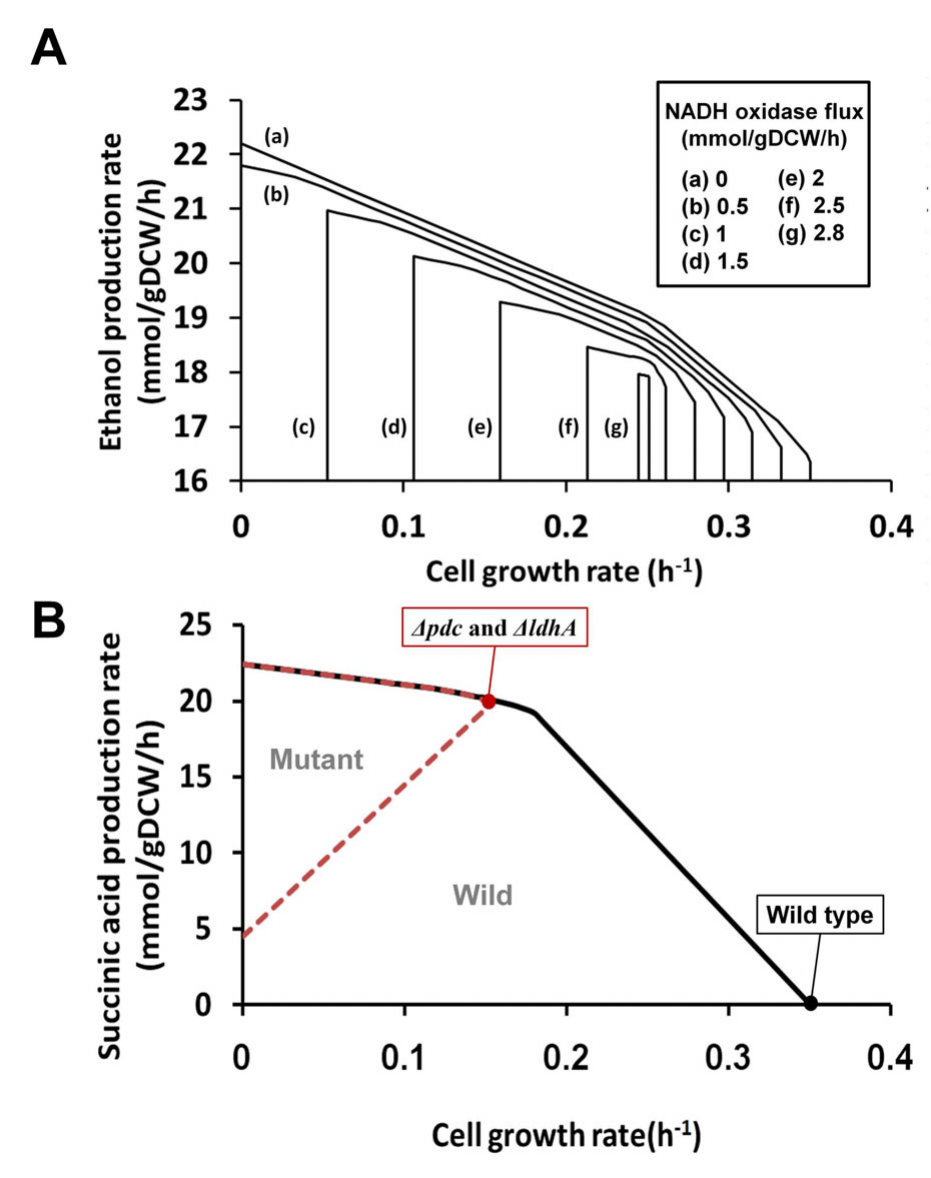
**Characteristics of aerobic shift and strategies for succinic acid production in *Z. mobilis***. (A) Trade-off curves of *Z. mobilis *for the flux rate of NADH oxidase reaction, ethanol production rate, and growth rate under aerobic condition. The relationship between growth rate and ethanol production rate was investigated for several flux levels of NADH oxidase reaction constrained by 0, 1, 2, and 2.8 mmol/gDCW/h under aerobic condition. The vertical lines of each graph that meet x-axis drop to zero. (B) Trade-off curves of wild type and mutant (*Δpdc *and *ΔldhA*) strains of *Z. mobilis *for succinic acid production under anaerobic condition. The black solid line and the red dotted line indicate the flux solution space of the wild type and mutant strain of *Z. mobilis*, respectively. Each circle denotes the state of the wild type and mutant strain of *Z. mobilis *on each maximal growth rate, respectively.

These characteristics of aerobic shift, which is the environmental change from anaerobic to aerobic condition, are reflected in ZmoMBEL601, and were validated by performing simulations using constraints-based flux analysis and comparing the results to published data. The growth rate and ethanol production rate in *Z. mobilis *were modeled at several different values of the constraints (0, 1, 2, and 2.8 mmol/gDCW/h) applied to the NADH oxidase activity during the simulation under aerobic condition (Figure 3A). The predicted growth and ethanol production rate were found to decrease gradually as the flux rate of NADH oxidase increased (Figure 3A). Additionally, because the NADH oxidase utilizes O_2 _to oxidize NADH, the activity level of NADH oxidase is directly correlated to the O_2 _uptake rate. Therefore, it can say that the production rate of ethanol and growth rate decreased as the O_2 _uptake rates increased [16,49]. These consistent simulation outcomes validate ZmoMBEL601.

### Analysis of flux-sum intensity between *Z. mobilis *and *E. coli*

The analysis of flux-sum intensity between *Z. mobilis *and *E. coli *was carried out with the three carbon sources, glucose, fructose, and sucrose, under anaerobic condition with defined minimal medium (Figure 2B) [50,51]. The flux-sum is defined as half of the summation of all consumption and generation fluxes around a particular metabolite under pseudo-steady state [50,51]. As the flux-sum is closely related to the turnover rate of metabolites, the metabolic state of the system can be elucidated through flux-sum analysis that examines the interconversion pattern of specific metabolites comprising of the network [51].

Metabolites, including some cofactors, amino acids, and others involved in central carbon metabolic pathways, were selected and categorized into a few groups to display the characteristic of each pathway. Flux-sum intensity of all the aforementioned metabolites, except for F6P in *Z. mobilis*, and for bDG6P, F6P and FDP in *E. coli *shows equal levels whether glucose or fructose was fed as a carbon source. The reason for the different flux-sum intensity of bDG6P, F6P, and FDP under two different carbon sources (i.e. glucose and fructose) is that glucose is metabolized to glucose-6-phosphate *via *glucokinase, and fructose is metabolized to glucose-6-phosphate *via *fructokinase and phosphoglucose isomerase. The strongest intensity value for the flux-sum was obtained from growth on sucrose because of its disaccharide chemical structure of glucose and fructose, resulting in having the effects of two carbon sources at the same time.

The different flux-sum intensity pattern of the metabolites in *Z. mobilis *and *E. coli *can be explained through the differences in the structure of their metabolic network. The metabolic network of *Z. mobilis *is small and even if some of the branch points are to be deleted, such as Pfk (phosphofructokinase) in glycolytic pathway, SucA (2-oxoglutarate dehydrogenase), and Mdh (malate dehydrogenase) in TCA cycle, there is little chance for the carbon flux to be distributed through other metabolic pathways (Figure 2A). *Z. mobilis *essentially has a linear like central metabolic pathway, including ED pathway and incomplete TCA cycle, and this allows for the metabolites in *Z. mobilis *to display flux-sum intensities of similar level. On the other hand, *E. coli *has a relatively large and robust metabolic network, which allows the uptake carbon flux to be redistributed through alternate pathways other than the central metabolic pathway. Thus, the variation of flux-sum intensity about metabolites in *E. coli *is larger than that of *Z. mobilis*. Flux-sum intensity of metabolites in glycolytic pathway in *E. coli *was found to be stronger than *Z. mobilis *under the three carbon sources. This was expected as *Z. mobilis *has an incomplete glycolytic pathway and the carbon flux of *Z. mobilis *flows through the ED pathway. For the same reason, *Z. mobilis *shows a stronger flux-sum intensity of metabolites in the ED pathway compared to that of *E. coli*. As *E. coli *possesses the pathway from acetyl-CoA to acetic acid through acetyl phosphate, which is absent in *Z. mobilis*, the acetyl-CoA pool of *E. coli *is converted to the acetic acid under anaerobic condition to generate ATP *via *substrate-level phosphorylation. Therefore, the flux-sum intensity of TCA cycle metabolites in *E. coli *is lower than that of *Z. mobilis*, which distributes its acetyl-CoA pool to the TCA cycle. Additionally, precursors of many amino acids that are generated through the TCA cycle subsequently display a relatively more intensive flux-sum value in *Z. mobilis *compared to *E. coli*.

### Analysis of maximum *in silico *yields of ethanol between *Z. mobilis *and *E. coli*

*Z. mobilis *has attracted attention for its high ethanol producing ability, and therefore many studies have been conducted regarding this topic [14,52,53]. Through in-depth researches, pyruvate decarboxylase in *Z. mobilis*, which converts pyruvate to acetaldehyde, has been shown to be one of the important reasons for the high ethanol producing capability in *Z. mobilis *[54]. Additionally, there are reports of genetically engineered *E. coli *possessing the pyruvate decarboxylase gene from *Z. mobilis *having increased ethanol production capability compared to the wild type strain [55-58]. Therefore, the role of pyruvate decarboxylase enzyme in ethanol production was examined using the *Z. mobilis *metabolic model ZmoMBEL601 by analysis of the maximum *in silico *yields of ethanol, and compared the results with analysis from the *E. coli *metabolic models EcoMBEL979 and iAF1260 [30,59].

Maximum *in silico *yields of ethanol in the *Z. mobilis *and *E. coli *metabolic models were 2 and 1.85 mol ethanol/mol glucose, respectively where EcoMBEL979 and *i*AF1260 showed the same yield. The result was in agreement with reports showing *Z. mobilis *is more capable of producing ethanol [56,60]. To evaluate the role of pyruvate decarboxylase about ethanol production in *Z. mobilis*, the simulation, where the pyruvate decarboxylase in *Z. mobilis *was removed from the *Z. mobilis *metabolic model, was performed by knocking out the respective metabolic reaction in the *Z. mobilis *metabolic model. The results of the simulation showed that the capacity for ethanol production dropped when pyruvate decarboxylase was removed from the *Z. mobilis *metabolic model (i.e. 0.03 mol ethanol/mol glucose from 2 mol ethanol/mol glucose). This outcome was similar to the reported experimental result of Seo *et al*. [61] that the pyruvate decarboxylase knockout mutant did not produce ethanol by fermentation process. Thus, it can be concluded that the pyruvate decarboxylase reaction is the essential part of producing ethanol in *Z. mobilis*. Next, the pyruvate decarboxylase reaction was introduced into the *E. coli *metabolic model to verify its role for ethanol production in *E. coli*. With the addition of the pyruvate decarboxylase reaction, the maximum *in silico *yield in the *E. coli *metabolic model improved to 2 mol from 1.85 mol ethanol/mol glucose. To further investigate the exact impact of pyruvate decarboxylase, other reactions of the *E. coli *metabolism, which can be utilized in the production of ethanol, but not found in *Z. mobilis*, were removed to determine its role in the production of ethanol. If the removal of the reactions resulted in a decrease in ethanol production, then the pyruvate decarboxylase reaction was concluded to not be the sole factor for the improved yield in *E. coli*. The significant differences of reactions between *Z. mobilis *and *E. coli *are that *E. coli *has only the phosphofructokinase reaction in glycolytic pathway, the 2-oxoglutarate dehydrogenase reaction and the malate dehydrogenase reaction in TCA cycle. Therefore, these reactions, which do not exist in *Z. mobilis*, were removed from the *E. coli *metabolic model. Despite the removal of all these reactions, the maximal *in silico *ethanol yields remained at 2 mol ethanol/mol glucose through alternative available metabolic pathways. Furthermore, the elimination of other reactions, such as glutamate dehydrogenase, ethanolamine ammonia-lyase, and acetaldehyde dehydrogenase, did not affect the maximum *in silico *yield of ethanol in *E. coli *metabolic model. Through these simulation results, it can be concluded that pyruvate decarboxylase is the main factor that allows *Z. mobilis *to have a greater capacity for ethanol production than *E. coli*. The presence of the other reactions were not seen to have direct correlation with the ethanol production capacity in *E. coli.*

The reaction above not found in *Z. mobilis*, were examined in their role in producing ethanol in *E. coli *using the wild type *E. coli *metabolic model, which lacks pyruvate decarboxylase. The reaction corresponding to the enzyme of transaldolase was removed, and resulted in the declined ethanol production yield, 1.78 mol ethanol/mol glucose. Because pentose phosphate pathway generates NADPH, the deletion of transaldolase reaction resulted in the decreased supply of reducing power. Therefore, it implies that the pentose phosphate pathway has an important role for producing ethanol in the *E. coli *wild type strain. It should be noted that EcoMBEL979 and *i*AF1260 produced the same results for this study.

### Expanding carbon source utilization of *Z. mobilis*

*Z. mobilis *cannot utilize pentose sugars due to incomplete metabolic pathways of pentose [16,39,62,63]. Because the feedstock holds a significant portion of cost in bioprocess, developing pentose sugar fermenting strains can reduce the cost by enabling strains to use cheap, renewable, lignocellulosic biomass. Several genes involved in pentose sugar metabolism, including those encoding xylose isomerase, xylulokinase, and transaldolase for utilizing xylose and arabinose isomerase, ribulokinase, ribulose-5-phosphate-4-epimerase, and transaldolase for utilizing arabinose, respectively, are absent in *Z. mobilis*. However, Zhang *et al*. [64] successfully introduced four genes (xylose isomerase, xylulokinase, transaldolase, and transketolase) into *Z. mobilis *for utilizing xylose as a carbon source. De Graaf *et al*. [63] also reported that *Z. mobilis *CP4 produces ethanol with xylose as a carbon source by introducing the same enzymes. Deanda *et al*. [65] reported that five genes (arabinose isomerase, ribulokinase, ribulose-5-phosphate-4-epimerase, transaldolase, transketolase) were necessary for *Z. mobilis *to utilize arabinose as a carbon source. According to these results, new pathways (three pathways for xylose and four pathways for arabinose; the transketolase gene was annotated and originally included in the metabolic model) were introduced in the metabolic model ZmoMBEL601 (Figure 2A; Additional file 1). Then, xylose and arabinose in the metabolic model were converted to xylulose 5-phophate *via *xylulose and ribulose 5-phosphate, respectively, and the fluxes through the pentose phosphate pathway were increased through producing more ribose-5-phosphate from ribulose 5-phosphate [63]. By utilizing the metabolic model, *in silico *simulations can be performed to predict the capability of *Z. mobilis *to metabolize carbon sources on anaerobic growth, and thereby allow for the expansion in the number of available carbon source [16,39,63-65].

For further application of engineered *Z. mobilis *metabolic model, the simulations were performed to investigate the maximum *in silico *yield of ethanol for three carbon sources (i.e. glucose, xylose, arabinose) in ZmoMBEL601 (Additional file 5A). Because of the difference in the substrates' carbon number, glucose showed a higher maximum *in silico *yield of ethanol and growth rate than those of xylose and arabinose. Additionally, to examine the relationship between biomass and ethanol production, single reaction knockout simulation was performed and the trade-off curves for each were plotted (additional file 5B). As the reactions in central metabolic pathway (i.e. glycolytic pathway, pentose phosphate pathway, and TCA cycle) have significant importance in the metabolism, the range of simulation (i.e. the place of knockout reaction) was limited to the central metabolic pathway. The results displayed 15 different cases in trade-off curves for glucose and 13 for arabinose and xylose. Out of these cases we focused on four of them: reactions which are essential to growth but not to ethanol production (i.e. type 2), reactions which are essential to ethanol production but not to growth (i.e. type 3), reactions which are essential to both growth and ethanol production (i.e. type 4), and reactions which are not essential to both growth and ethanol production but result the rapid decrease in ethanol production when deleted (i.e. type 7). In the case of type 2 reactions, the lists of reactions are the same for both xylose and arabinose, but glucose has an additional reaction in the list: ribose 5-phosphate isomerase. Alcohol dehydrogenase was the only reaction in the type 3 case and is essential for the ethanol production regardless of the carbon source. In the case of type 4 reactions, the reaction lists of each carbon were directly related to its initial utilization pathway: reactions in ED pathway for every carbon sources and additional reactions in pentose phosphate pathway for xylose and arabinose. Thus, both growth and ethanol production were impossible when these reactions are deleted. Pyruvate decarboxylase is a type 7 reaction and its deletion resulted in a drop but not complete elimination of the ethanol production rate.

### Strategies for succinic acid production in *Z. mobilis*

*Z. mobilis *has the potential for the overproduction of a chemical that is of industrial value by redirecting metabolic pathways upon gene knockout. One of the examples would be succinic acid, a four carbon dicarboxylic acid and intermediate of TCA cycle. Additionally, it is an important industrial product useful for pharmaceutical and chemical intermediates, additives in the food industry, fertilizers, solvents, and polymers [66,67]. The biotechnological overproduction of succinic acid in *Z. mobilis *was investigated through gene knockout simulation using constraints-based flux analysis implemented by constraining the flux value of the respective knockout reaction to zero. For single gene knockout simulation, satisfactory targets that produce succinic acid were not obtained, compared with previous knowledge on the production level of succinic acid. The best result of the single-gene knockout simulation for succinic acid production gave low yields of succinic acid only 0.15% of theoretical maximum yield for succinic acid production (2 mol succinic acid/mol glucose). Simulations of double gene knockout, however, resulted in the combination of pyruvate decarboxylase and D-lactate dehydrogenase as the best targets for succinic acid overproduction (Figure 3B), and the pairs of gene targets are presented in the additional file 6. Functional and physical relationship among reactions in metabolic network, utilized to discover potential combinatorial engineering targets, can be revealed by applying grouping reaction constraint to constraints-based flux analysis [68]. Hence, additional gene knockout simulations that contain grouping reaction constraints were performed, and obtained the same results. These analyses revealed that the inactivation of pyruvate decarboxylase essential for ethanol production redirected the metabolic fluxes towards lactic acid production, and the inactivation of D-lactate dehydrogenase redirected the metabolic fluxes toward succinic acid production in TCA cycle. These results agree well with the work of Seo *et al*. [61], which obtained a result of a 95% theoretical yield of succinic acid by performing the gene knockout for pyruvate decarboxylase and D-lactate hydrogenase. On the other hand, *E. coli *metabolic model did not show significant increment in the succinic acid production when the same target D-lactate dehydrogenase was knocked out; *E. coli *does not have pyruvate decarboxylase. This is because of the many other alternative pathways in *E. coli *which can complement and relieve the constraints of gene deletion. Therefore, *E. coli *requires its own specific gene target combinations, which are distinct from *Z. mobilis*. As the reconstructed ZmoMBEL601 was able to design gene knockout strategy for succinic acid production successfully, it could suggest the strategies for other biochemicals as well, such as lactic acid, fumaric acid, and malic acid.

## Conclusions

We have presented the genome-scale reconstruction and analysis of metabolic network in *Z. mobilis *ZM4. The metabolic model was constructed systematically *via *four steps; automatic building, manual curation, rational determination of the biomass composition, and completion of the metabolic model. The metabolic model reflects the physiological characteristics of *Z. mobilis*, including the ED pathway, incomplete pentose phosphate pathway, oxidative phosphorylation mechanisms, and high ethanol producing ability. The metabolic model was utilized to investigate the characteristics of ethanol production and was further characterized through the comparison with the *E. coli *metabolic model. The metabolic model allowed for the development of strategies for strain improvement, including the addition of several pathways to the metabolic model to allow for the metabolism of pentose sugar in *Z. mobilis *ZM4, strategies for ethanol and succinic acid production in *Z. mobilis *ZM4, and constraints-based flux analysis to give an accurate representation of phenotypes that match with reported data. As a consequence of analysis, validation, and application mentioned above, the genome-scale reconstructed metabolic model of *Z. mobilis *ZM4, ZmoMBEL601, is expected to be useful for systematical designing of strain development strategy for biotechnology applications.

## Materials and methods

### Reconstruction of metabolic model

Metabolic model of the *Z. mobilis *ZM4 was constructed by using the combined information from many different sources, including public databases, literatures, and experiments. The construction of the metabolic model was carried out with four distinct steps: First, automatic building of a draft metabolic model with data retrieved from databases; from genomes to metabolic pathways. Second, the draft metabolic model is manually curated through literature information. Third, rational determination of the biomass composition and maintenance requirements are done by experimental and literature data. Finally, validation of the metabolic model with the information obtained from previous steps and correctly modify with literatures and experimental results (Figure 1).

For efficient construction of the *Z. mobilis *ZM4 metabolic model, we used different softwares and databases, which include NCBI [23], CMR [24], KEGG [20], TCDB [26], TransportDB [27], ExPASy [25], BioCyc [21], BioSilico [28], MFAML [69], and MetaFluxNet [70].

### Constraints-based flux analysis

Constraints-based flux analysis is a method for studying metabolic networks with the assumption of a pseudo-steady state and constraints are imposed by mass balance of the metabolites [4,71]. This pseudo-steady state approximation is generally valid because the metabolites concentrations tend to reach to equilibrium much faster compared to genetic regulation [72]. This results in a stoichiometric model *S*_*ij *_• *v*_*j *_= *0*, in which *S*_*ij *_is a stoichiometric coefficient of a metabolite *i *in the *j*^th ^reaction and *v*_*j *_is the flux of the *j*^th ^reaction given in mmol/gDCW/h. Additional constraints can also be introduced to represent measured or imposed values for metabolites and are implemented as inequalities. Thus, fermentations were conducted to observe cellular behaviors and to measure key metabolites concentrations, which were used in constraints-based flux analysis (Figure 1; Additional file 7). With the uptake rate and the by-product secretion rate, we imposed upper and lower constraints in the *Z. mobilis *metabolic model (i.e. limit the flux on fumaric acid, acetic acid, malic acid, acetoin, and acetaldehyde production) for regulating the flux more realistically. The calculation of the maximum *in silico *yields of ethanol was done using the metabolic model of *Z. mobilis *(ZmoMBEL601) and *E. coli *(EcoMBEL979 and iAF1260). As the pyruvate dehydrogenase reaction in *E. coli *has low activity under anaerobic conditions [73], the pyruvate dehydrogenase reaction was inactivated in the *E. coli *metabolic models. Uptake rate of glucose is fixed to 10 mmol/gDCW/h, and reaction for oxygen uptaking was deleted to describe anaerobic condition in both genome-scale metabolic models. The NGAME (i.e. non-growth associated maintenance energy) values for both genome-scale metabolic models were eliminated. Ethanol production rate are normalized to the glucose uptake rate.

### Analysis of flux-sum intensity

The flux-sum is defined as the half of the summation of all consumption and generation rate around a particular metabolite under pseudo steady-states [50,51]. To understand the state of a cellular metabolism, the intensity of flux-sum for each metabolite was analyzed, as a good property for investigating the interconversion of metabolites. The flux-sum of metabolite *i *is formulated as $\frac{1}{2}\sum_{j}\left|S_{ij}\cdot v_{j}\right|$, where *S*_*ij *_is the stoichiometric coefficient of a metabolite *i *in the *j*^th ^reaction and *v*_*j *_is the flux of the *j*^th ^reaction. The *S*_*ij *_• *v*_*j *_means the absolute values of consumption or generation rate of metabolite *i *in the *j*^th ^reaction. The flux-sum of metabolite *i *is calculated by dividing the *S*_*ij *_• *v*_*j *_by one half. The flux-sum intensities of metabolites from central metabolism in *Z. mobilis *were analyzed to investigate the turnover rate of each metabolite using the ZmoMBEL601 model and compared to the flux-sum intensity of each metabolite in *E. coli *using the EcoMBEL974 model. The analysis of flux-sum intensity between both organisms was carried out with the three kinds of carbon sources including glucose, fructose, and sucrose.

### Media and cultivation

Frozen *Z. mobilis *ZM4 strain stock (with 70% glycerol) at -70°C was inoculated into a sealed tube containing 10 mL of ZM medium. ZM medium contains per liter: 50 g glucose, 2 g KH_2_PO_4_, 1 g (NH_4_)_2_SO_4_, 1 g MgSO_4_·7H_2_O, 10 g yeast extract. After incubating at 30°C for 12 hrs, the culture was transferred to a 500 mL sealed flask containing 100 mL ZM medium for subculture. Fermentation was carried out at 30°C in a 6.6 L bioreactor (BioFlo 3000, New Brunswick Scientific, Edison, NJ, USA) containing 2 L of ZM medium. The pH was controlled at 5.5 with 5 M NaOH. Anaerobic condition was achieved by 50 rpm agitation speed and flushing the bioreactor with oxygen-free CO_2 _gas (Kosock gas, Daejeon, Korea). In case of aerobic culture, the dissolved oxygen concentration was maintained above 40% of air saturation by supplying air at 1 vvm (air volume/working volume/minute) and by automatically controlling the agitation speed up to 1,000 rpm.

### Analytical procedures

The cell growth was monitored by measuring the absorbance at 600 nm using an Ultrospec 3000 spectrophotometer (Pharmacia Biotech., Uppsala, Sweden). Metabolites were analyzed by high-performance liquid chromatography (Varian ProStar 210, Palo Alto, CA, USA), equipped with an Aminex HPX-87H column (300 mm × 7.8 mm, Bio-Rad Laboratories, Herculules, CA, USA), UV/VIS (Varian ProStar 320, Palo Alto, CA, USA) and a refractive index (Shodex RI-71, Minato-Ku, Tokyo, Japan) detectors. The column was eluted isocratically at 50°C at a flow rate of 0.5 mL/min with 0.01 N H_2_SO_4_.

### Biomass composition

Biosynthetic pathways and composition of each molecules/metabolites of proteins, DNA, RNA, lipids, small molecules pool, and cell wall were formulated with literature data or reasonably assumed as described in additional file 4. Many types of data were collected not only from *Z. mobilis *ZM4 and various *Z. mobilis *strains (e.g., *Z. mobilis *CP4) but also from other species for this purpose, because it was not possible to describe all the formulation mechanism with only the data from *Z. mobilis *ZM4. The data was properly manipulated and the biomass composition and reaction of *Z. mobilis *ZM4 strain was determined (Additional file 4).

## Lists of abbreviations

The abbreviations are represented as follows: Acn: aconitate hydratase; Adh: alcohol dehydrogenase; AraA: L-arabinose isomerase; AraB: L-ribulokinase; Cit: citrate lyase; Cs: citrate synthase; Eda: 2-dehydro-3-deoxyphosphogluconate aldolase; Edd: phosphogluconate dehydratase; Eno: enolase; Fba: fructose-bisphosphate aldolase; Fum: fumarate hydratase; Glk: glucokinase; Icd: isocitrate dehydrogenase; Ldh: L-lactate dehydrogenase; Mdh: malate dehydrogenase; Me: Malic enzyme; Pdc: pyruvate decarboxylase; Pfk: 6-phosphofructokinase; Pgi: glucose-6-phosphate isomerase; Pgl: 6-phosphogluconolactonase; Ppc: phosphoenolpyruvate carboxylase; SacA: beta-fructofuranosidase; SacB: levansucrase; ScrK: fructokinase; Sdh: succinate dehydrogenase; Sgb: L-ribulose-5-phosphate 4-epimerase; Suc: succinyl-CoA synthetase; SucA: 2-oxoglutarate dehydrogenase; Tkt: transketolase; Xk: xylulokinase; Xyl: xylose isomerase; Zwf: glucose-6-phosphate 1-dehydrogenase; DCW: dry cell weight;.

## Competing interests

The authors declare that they have no competing interests.

## Authors' contributions

KYL, JMP, TYK, HSY, and SYL generated ideas; KYL, JMP, TYK, and SYL designed research; KYL, JMP, and SYL performed research; KYL and JMP performed analytical experiments; KYL, JMP, and SYL analyzed data; KYL, JMP, TYK, HSY, and SYL wrote the paper. All authors read and approved the final version of the manuscript.

## Supplementary Material

Additional file 1 List of metabolic reactions in the genome-scale metabolic model of *Zymomonas mobilis *ZM4 Click here for file

Additional file 2 List of metabolites in the genome-scale metabolic model of *Zymomonas mobilis *ZM4 Click here for file

Additional file 3 ORF (Open Reading Frame) coverage of reconstructed metabolic models Click here for file

Additional file 4 Biomass composition of *Zymomonas mobilis *ZM4 Click here for file

Additional file 5 **Trade-off curves of (A) engineered strain of *Zymomonas mobilis *for the utilization of xylose and arabinose and (B) single gene knockout mutants based on the engineered strain for glucose, xylose, and arabinose as a carbon source**. Click here for file

Additional file 6 Double gene knockout targets for succinic acid production identified by constraints-based flux analysis Click here for file

Additional file 7 Batch culture profile of *Zymomonas mobilis *ZM4 Click here for file
