# Supplementary material for: The genome-scale metabolic network analysis of Zymomonas mobilis ZM4 explains physiological features and suggests ethanol and succinic acid production strategies
Source: Microb Cell Fact. 2010 Nov 24;9:94. doi: 10.1186/1475-2859-9-94 (PMC3004842; doi:10.1186/1475-2859-9-94)
Supplement: Additional file 1 — List of metabolic reactions in the genome-scale metabolic model of Zymomonas mobilis ZM4 [file 1475-2859-9-94-S1.PDF]

Additional file 1. List of metabolic reactions in the genome-scale metabolic model of *Zymomonas mobilis* ZM4

Metabolism	Number	Gene Name	Enzyme	EC Number	Reaction	[1]Database	Ref.
**Carbohydrate Metabolism**							
Glycolysis / Gluconeogenesis	R001	ZMO1212	glucose-6-phosphate isomerase	5.3.1.9	G6P <-> bDG6P	KG	
	R002	ZMO1212	glucose-6-phosphate isomerase	5.3.1.9	G6P <-> F6P	KG	
	R003	ZMO1212	glucose-6-phosphate isomerase	5.3.1.9	bDG6P <-> F6P	KG	
	R004	ZMO0369	glucokinase	2.7.1.2	GLC + ATP <-> G6P + ADP	KG	
	R005	ZMO0889	aldose 1-epimerase	5.1.3.3	bDGLC <-> GLC	KG	
	R006	ZMO0482	fructose-1,6-bisphosphatase	3.1.3.11	FDP -> F6P + PI	KG	
	R007	ZMO0179	fructose-bisphosphate aldolase	4.1.2.13	FDP <-> T3P1 + T3P2	KG	
	R008	ZMO0465	triosephosphate isomerase	5.3.1.1	T3P1 <-> T3P2	KG	
	R009	ZMO0177	glyceraldehyde-3-phosphate dehydrogenase	1.2.1.12	T3P1 + PI + NAD <-> NADH + 13DPG	KG	
	R010	ZMO0178	phosphoglycerate kinase	2.7.2.3	13DPG + ADP <-> 3PG + ATP	KG	
	R011	ZMO1240	phosphoglycerate mutase	5.4.2.1	3PG <-> 2PG	KG	
	R012	ZMO1608	enolase	4.2.1.11	2PG <-> PEP	KG	
	R013	ZMO0152	pyruvate kinase	2.7.1.40	PEP + ADP -> PYR + ATP	KG	
	R014	ZMO1605 AND ZMO1606	pyruvate dehydrogenase E1 component subunit alpha AND beta	1.2.4.1	PYR + THPP -> 2HETHPP + CO2	KG	
	R015	ZMO1605 AND ZMO1606	pyruvate dehydrogenase E1 component subunit alpha AND beta	1.2.4.1	2HETHPP + LIPO -> ADLIPO + THPP	KG	
	R016	ZMO0510	pyruvate dehydrogenase E2 component	2.3.1.12	COA + ADLIPO -> DLIPO + ACCOA	KG	
	R017	ZMO0512	dihydrolipoamide dehydrogenase	1.8.1.4	DLIPO + NAD -> LIPO + NADH	KG	
	R018		pyruvate decarboxylase	4.1.1.1	PYR -> ACAL + CO2		Yang *et al* (2009)
	R019	ZMO1236 AND ZMO1596 or ZMO1722	alcohol dehydrogenase or S-(hydroxymethyl)glutathione dehydrogenase	1.1.1.1 or 1.1.1.284	ACAL + NADH <-> ETH + NAD	KG	
	R020		phosphoglyceromutase	5.4.2.2	G6P <-> G1P	BC	
	R021	ZMO0369	glucokinase	2.7.1.2	bDGLC + ATP -> bDG6P + ADP	KG	
Citrate cycle (TCA cycle)	R022	ZMO1963	citrate synthase	2.3.3.1	ACCOA + OA -> COA + CIT	KG	
	R023	ZMO0487	citrate lyase subunit beta	4.1.3.6	CIT <-> AC + OA	KG	
	R024	ZMO0543	aconitate hydratase 1	4.2.1.3	CIT <-> ICIT	KG	
	R025	ZMO0544	isocitrate dehydrogenase	1.1.1.42	ICIT + NADP <-> CO2 + NADPH + AKG	KG	
	R026	ZMO1307	fumarate hydratase, class I	4.2.1.2	FUM <-> MAL	KG	
	R027	ZMO0569	succinate dehydrogenase	1.3.99.1	FUM + FADH2 <-> SUCC + FAD	KG	
	R028	ZMO0567 AND ZMO1481	succinyl-CoA synthetase alpha AND beta subunit	6.2.1.5	SUCCOA + ADP + PI <-> ATP + COA + SUCC	KG	
Pentose phosphate pathway	R029	ZMO1649	gluconolactonase	3.1.1.17	GLCNDL -> GLCN	KG	
	R030	ZMO1757	gluconokinase	2.7.1.12	ATP + GLCN -> ADP + D6PGC	KG	
	R031	ZMO0997	2-dehydro-3-deoxyphosphogluconate aldolase AND 4-hydroxy-2-oxoglutarate aldolase	4.1.2.14 AND 4.1.3.16	2KD6PG -> T3P1 + PYR	KG	
	R032	ZMO0368	phosphogluconate dehydratase	4.2.1.12	D6PGC -> 2KD6PG	KG	
	R033	ZMO1478	6-phosphogluconolactonase	3.1.1.31	D6PGL -> D6PGC	KG	
	R034		6-phosphogluconate dehydrogenase	1.1.1.44	D6PGC + NADP -> NADPH + CO2 + RL5P	BC	
	R035	ZMO0367	glucose-6-phosphate 1-dehydrogenase	1.1.1.49	bDG6P + NADP <-> D6PGL + NADPH	KG	
	R036	ZMO0176	transketolase	2.2.1.1	X5P + E4P <-> F6P + T3P1	KG	
	R037	ZMO0176	transketolase	2.2.1.1	R5P + X5P <-> T3P1 + S7P	KG	
	R038	ZMO0018	ribulose-phosphate 3-epimerase	5.1.3.1	RL5P <-> X5P	KG	
	R039	ZMO1200	ribose 5-phosphate isomerase B	5.3.1.6	RL5P <-> R5P	KG	
	R040		D-Ribose 1,5-phosphomutase	5.4.2.2	R5P <-> R1P	BC	
	R041	ZMO0787	gluconate 2-dehydrogenase	1.1.1.215	GLUC + NADP <-> 2kGLUC + NADPH	KG	
Pentose and glucuronate interconversions	R042	ZMO0819	UDPglucose 6-dehydrogenase	1.1.1.22	UDPG + 2 NAD <-> UDPGLC + 2 NADH	KG	
	R043	ZMO1767	UTP-glucose-1-phosphate uridylyltransferase	2.7.7.9	UTP + G1P <-> PPI + UDPG	KG	
Fructose and mannose	R044	ZMO0788	D-sorbitol dehydrogenase (acceptor)	1.1.99.21	SOB + FADH2 -> SOT + FAD	KG	
	R045	ZMO0339	phosphomannomutase	5.4.2.8	MAN6P <-> MAN1P	KG	
	R046	ZMO1233	mannose-6-phosphate	5.3.1.8	MAN6P <-> F6P	KG	
	R047	ZMO0179	fructose-bisphosphate aldolase, class I	4.1.2.13	F1P <-> T3P1 + T3P2	KG	
	R048	ZMO1771	iron-containing alcohol dehydrogenase	1.1.1.-	S6P + NADP <-> SB1P + NADPH	KG	
Galactose metabolism	R049	ZMO0904	beta-galactosidase	3.2.1.23	LCTS -> GLAC + GLC	KG	
	R050	ZMO0375 or ZMO0942	beta-fructofuranosidase	3.2.1.26	RAF -> MELI + FRU	KG	
	R051	ZMO0375 or ZMO0942	beta-fructofuranosidase	3.2.1.26	ST -> G6GG + FRU	KG	
Ascorbate and aldarate metabolism	R052	ZMO1649	gluconolactonase	3.1.1.17	g14l <-> guln	KG	

Metabolism	Number	Gene Name	Enzyme	EC Number	Reaction	[1]Database	Ref.
Starch and sucrose metabolism	R053	ZMO0374	levansucrase	2.4.1.10	SUC -> GLC + LEVAN	KG	
	R054	ZMO0375 AND ZMO0942	beta-fructofuranosidase	3.2.1.26	SUC -> FRU + GLC	KG	
	R055	ZMO1719	fructokinase	2.7.1.4	ATP + FRU <-> ADP + F6P	KG	
	R056	ZMO0153	glucose-1-phosphate adenylyltransferase	2.7.7.27	ATP + G1P -> ADPGLC + PPI	KG	
	R057		Glycogen synthase	2.4.1.21	ADPGLC -> ADP + GLYCOGEN	BC	Ivi C Tsantili *et al* (2006)
	R058		Glycogen phosphorylase	2.4.1.1	GLYCOGEN + PI -> G1P		Ivi C Tsantili *et al* (2006)
	R059	ZMO0375 AND ZMO0942	beta-fructofuranosidase	3.2.1.26	SUC6P -> FRU + G6P	KG	
Amino sugar and nucleotide sugar metabolism	R060	ZMO0962	N-acetylglucosamine-6-phosphate deacetylase	3.5.1.25	GA6P + AC <-> NAGP	KG	
	R061	ZMO0498	bifunctional protein GlmU	2.7.7.23 AND 2.3.1.157	UTP + NAG1P <-> PPI + UDPNAG	KG	
	R062	ZMO0498	bifunctional protein GlmU	2.7.7.23 AND 2.3.1.157	ACCOA + GA1P -> COA + NAG1P	KG	
	R063	ZMO1002	phosphoglucosamine mutase	5.4.2.10	GA6P -> GA1P	KG	
	R064	ZMO0056	glucosamine-fructose-6-phosphate aminotransferase (isomerizing)	2.6.1.16	GLN + F6P <-> GLU + GA6P	KG	
	R065	ZMO1724	UDP-N-acetylglucosamine 1-carboxyvinyltransferase	2.5.1.7	PEP + UDPNAG -> UDPNAGEP + PI	KG	
	R066	ZMO0833	UDP-N-acetylmuramate dehydrogenase	1.1.1.158	UDPNAGEP + NADPH -> UDPNAM + NADP	KG	
	R067	ZMO1171	beta-N-acetylhexosaminidase	3.2.1.52	CTB -> 2 NAGA	KG	
	R068	ZMO0833	UDP-N-acetylmuramate dehydrogenase	1.1.1.158	UDPNAGEP + NADH -> UDPNAM + NAD	KG	
	R069	ZMO0941	UDP-glucuronate 5'-epimerase	5.1.3.12	UDPGLC -> UDPIDU	KG	
	R070	ZMO1294	N-acetylmuramic acid-6-phosphate etherase	4.2.-.-	ACMU6P <-> NAGP + LAC	KG	
Pyruvate metabolism	R071	ZMO0030 or ZMO0760 or ZMO1721	lactoylglutathione lyase	4.4.1.5	RGT + MTGYX <-> SLGT	KG	
	R072	ZMO0759	hydroxyacylglutathione	3.1.2.6	SLGT <-> RGT + LAC	KG	
	R073	ZMO0256 or ZMO1237	D-lactate dehydrogenase	1.1.1.28	PYR + NADH <-> NAD + LAC	KG	
	R074	ZMO1496	phosphoenolpyruvate carboxylase	4.1.1.31	PEP + CO2 -> OA + PI	KG	
	R075	ZMO1570	formate C-acetyltransferase	2.3.1.54	PYR + COA -> ACCOA + FORT	KG	
	R076	ZMO1955	malate dehydrogenase (oxaloacetate-decarboxylating)	1.1.1.38	MAL + NAD <-> CO2 + NADH + PYR	KG	
	R077	ZMO0583 AND ZMO0599 or ZMO0735 AND ZMO0736	acetyl-CoA carboxylase carboxyl transferase subunit alpha AND beta or biotin carboxylase AND acetyl-CoA carboxylase, biotin carboxyl carrier protein	6.4.1.2 or 6.3.4.14	ACCOA + ATP + CO2 -> MALCOA + ADP + PI	KG	
	R078	ZMO0152	pyruvate kinase	2.7.1.40	NDP + PEP -> NTP + PYR	KG	
Glyoxylate and dicarboxylate metabolism	R079	ZMO0497 or ZMO1805	phosphoglycolate phosphatase	3.1.3.18	2PG -> GLYCOLATE + PPI	KG	
	R080	ZMO1883	gyoxylate AND hydroxypyruvate reductase A	1.1.1.79 AND 1.1.1.81	GLYCOAL + NADP <-> GLYCOLATE + NADPH	KG	
	R081	ZMO1181	N-formylglutamate deformylase	3.5.1.68	FORGLU -> FORT + GLU	KG	
	R082	ZMO0997	2-dehydro-3-deoxyphosphogluconate aldolase AND 4-hydroxy-2-oxoglutarate aldolase	4.1.2.14 AND 4.1.3.16	PYR + GLX <-> HYDROXYAKG	KG	
Propanoate metabolism	R083	ZMO1570	formate C-acetyltransferase	2.3.1.54	OBUT + COA <-> PPACOA + FORT	KG	
Butanoate metabolism	R084	ZMO0687 AND ZMO1139 AND ZMO1140	acetolactate synthase I/II/III large subunit AND acetolactate synthase I/III small subunit	2.2.1.6	TPP + PYR -> HTPP + CO2	KG	
	R085	ZMO1754	SUCC-semialdehyde dehydrogenase (NADP+)	1.2.1.16	SUCCSAL + NAD -> SUCC + NADH	KG	
	R086	ZMO1754	SUCC-semialdehyde dehydrogenase (NADP+)	1.2.1.16	SUCCSAL + NADP -> SUCC + NADPH	KG	
	R087	ZMO1771	iron-containing alcohol dehydrogenase	1.1.1.-	BUTAL + NADH <-> 1BOH + NAD	KG	
	R088	ZMO1771	iron-containing alcohol dehydrogenase	1.1.1.-	BUTAL + NADPH <-> 1BOH + NADP	KG	
C5-Branched dibasic acid metabolism	R089	ZMO0687 AND ZMO1139 AND ZMO1140	acetolactate synthase I/II/III large subunit AND acetolactate synthase I/III small subunit	2.2.1.6	2 PYR -> ACLAC + CO2	KG	
	R090		acetolactate decarboxylase	4.1.1.5	ACLAC -> ACTN + CO2		L. O. Ingram *et al* (1991)
	R091	ZMO0567 AND ZMO1481	succinyl-CoA synthetase alpha AND beta subunit	6.2.1.5	ATP + ITCN + COA <-> ADP + PI + ITCNCOA	KG	

Metabolism	Number	Gene Name	Enzyme	EC Number	Reaction	[1]Database	Ref.
Inositol phosphate	R092	ZMO0329	myo-inositol-1(or 4)-monophosphatase	3.1.3.25	I3P -> INSTOL + PPI	KG	
	R093		phosphatidylinositol synthase	2.7.8.11	CDPDG + INSTOL -> CMP + PINSTOL	BC	
	R094	ZMO0329	myo-inositol-1(or 4)-monophosphatase	3.1.3.25	I4P -> INSTOL + PPI	KG	
	R095	ZMO0329	myo-inositol-1(or 4)-monophosphatase	3.1.3.25	IP -> INSTOL + PPI	KG	
	R096	ZMO0061	4-phytase AND acid phosphatase	3.1.3.26 AND 3.1.3.2	IHKP -> IPKP + PI	KG	
**Energy Metabolism**							
Energy Metabolism	R097	ZMO0956	Ubiquinol-cytochrome-c reductase	1.10.2.2	QH2 <-> Q + 4 HEXT	KG	
	R098	ZMO1507	Inorganic pyrophosphatase	3.6.1.1	PPI -> 2 PI	KG	
	R099	ZMO0712	Polyphosphate kinase	2.7.4.1	ATP + PPI <-> ADP + PPPI	KG	
	R100	ZMO0238	atpH;ATP synthase delta subunit	3.6.3.14	ATP <-> ADP + PI + 2 HEXT	KG	
	R101	ZMO1753	ferredoxin-NADP reductase	1.18.1.2	RFEDX + NADP <-> OFRDX + NADPH	KG	
	R102	ZMO0569	succinate dehydrogenase	1.3.99.1	FUM + QH2 <-> SUCC + Q	KG	Seo *et al* (2005)
	R103				QH2 + 2 FAD -> Q + 2 FADH2 + 2 HEXT		adding reaction
	R104				NADH + Q -> NAD + QH2 + 2 HEXT		adding reaction
	R105			1.6.1.1	NADPH + NAD <-> NADP + NADH	BC	
	R106				NADH + 0.5 O2 -> NAD		Kim *et al* (1995)
Methane metabolism	R107	ZMO0918	catalase	1.11.1.6	H2O2 + METHANOL -> FALD	KG	
	R108	ZMO1747	methylenetetrahydrofolate reductase [NAD(P)H];	1.5.1.20	METTHF + FADH2 <-> MTHF + FAD	KG	
	R109	ZMO1722	S-(hydroxymethyl)glutathione dehydrogenase AND alcohol dehydrogenase	1.1.1.284 AND 1.1.1.1	HMGT + NAD -> FMGT + NADH	KG	
Nitrogen metabolism	R110	ZMO1133	carbonic anhydrase	4.2.1.1	H2CO3 <-> CO2	KG	
	R111	ZMO1116 AND ZMO1117	glutamate synthase (NADPH/NADH) small AND large chain	1.4.1.13 AND 1.4.1.14	GLN + AKG + NADPH -> 2 GLU + NADP	KG	
	R112	ZMO0493	glutamine synthetase	6.3.1.2	ATP + GLU + NH3 <-> ADP + PI + GLN	KG	
	R113	ZMO1823 AND ZMO1824 AND ZMO1825	nitrogenase iron protein NifH AND nitrogenase molybdenum-iron protein alpha AND beta chain	1.18.6.1	8 RFEDX + 16 ATP + N2 -> 8 OFRDX + 16 PI + 16 ADP + 2 NH3	KG	
	R114	ZMO1116 AND ZMO1117	glutamate synthase (NADPH/NADH) small AND large chain	1.4.1.13 AND 1.4.1.14	GLN + AKG + NADH -> 2 GLU + NAD	KG	
	R115	ZMO1207	nitrilase	3.5.5.1	RCN -> RCO2 + NH3	KG	
Sulfur metabolism	R116	ZMO0004 AND ZMO0005	sulfate adenylyltransferase subunit 1 AND 2	2.7.7.4	ATP + SLF -> PPI + APS	KG	
	R117	ZMO0003	nylylsulfate kinase	2.7.1.25	ATP + APS <-> ADP + PAPS	KG	
	R118	ZMO0007	phosphoadenosine phosphosulfate reductase	1.8.4.8	PAPS + RTHIO -> PAP + H2SO3 + OTHIO	KG	
	R119	ZMO0008 AND ZMO0009	sulfite reductase (NADPH) hemoprotein alpha AND beta-component	1.8.1.2	H2SO3 + 3 NADPH -> H2S + 3 NADP	KG	
	R120	ZMO0225	homoserine O-acetyltransferase	2.3.1.31	ACCOA + HSER <-> COA + OAHSER	KG	
	R121	ZMO0327	cystathionine beta-lyase	4.4.1.8	LLCT -> HCYS + PYR + NH3	KG	
	R122		phosphoadenylate 3'-nucleotidase	3.1.3.7	PAP -> AMP + PI		Yang *et al* (2009)
	R123				CYS + OAHSER -> LLCT + AC		adding reaction
**Lipid Metabolism**							
Fatty acid biosynthesis	R124	ZMO1223	fabD; malonyl CoA-acyl carrier protein transacylase	2.3.1.39	MALCOA + ACP <-> MALACP + COA	KG	
	R125	ZMO1278	fabF; 3-oxoacyl-(acyl-carrier-protein) synthase	2.3.1.41	ACACP + COA <-> ACCOA + ACP	KG	
	R126	Dodecanoyl-[acyl-carrier protein] synthesis (lumped reaction)	Lauric acid (12:0)		ACACP + 5 MALACP + 10 NADPH -> 10 NADP + C120ACP + 5 CO2 + 5 ACP	KG	
	R127	Myristoyl-[acyl-carrier protein] synthesis (lumped	Myristic acid (14:0)		ACACP + 6 MALACP + 12 NADPH -> 12 NADP + C140ACP + 6 CO2 + 6 ACP	KG	
	R128	Tetradecanoyl-[acyl-carrier protein] synthesis (lumped reaction)	Myristoleic acid (14:1)		ACACP + 6 MALACP + 11 NADPH -> 11 NADP + C141ACP + 6 CO2 + 6 ACP	KG	
	R129	Hexadecanoyl-[acyl-carrier protein] synthesis (lumped reaction)	Palmitic acid (16:0)		ACACP + 7 MALACP + 14 NADPH -> 14 NADP + C160ACP + 7 CO2 + 7 ACP	KG	
	R130	Palmitoyl-[acyl-carrier protein] synthesis (lumped reaction)	Palmitoleic acid (16:1)		ACACP + 7 MALACP + 13 NADPH -> 13 NADP + C161ACP + 7 CO2 + 7 ACP	KG	

Metabolism	Number	Gene Name	Enzyme	EC Number	Reaction	[1]Database	Ref.
	R131	Stearoyl-[acyl-carrier protein] synthesis (lumped reaction)	Stearic acid (18:0)		ACACP + 8 MALACP + 16 NADPH -> 16 NADP + C180ACP + 8 CO2 + 8 ACP	KG	
	R132	Oleoyl-[acyl-carrier protein] synthesis (lumped reaction)	Vaccenic acid (18:1)		ACACP + 8 MALACP + 15 NADPH -> 15 NADP + C181ACP + 8 CO2 + 8 ACP	KG	
	R133	Octadecanoyl-[acyl-carrier protein] synthesis (lumped reaction)			C181ACP -> C190ACP	KG	
Glycero phopholipid metabolism	R134	ZMO1905	glycerol-3-phosphate dehydrogenase (NAD(P)+)	1.1.1.94	NADPH + T3P2 <-> GL3P + NADP	KG	
	R135	Phopholipid systhesis (lumped reacion)	glycerol-3-phosphate acyltransferase	2.3.1.15	GL3P + 0.007 C120ACP + 0.42 C140ACP + 0.007 C141ACP + 0.254 C160ACP + 0.175 C161ACP + 0.013 C180ACP + 0.112 C181ACP -> AGL3P + ACP	KG	
	R136	Phopholipid systhesis (lumped reacion)	1-acyl-sn-glycerol-3-phosphate acyltransferase	2.3.1.51	AGL3P + 0.007 C120ACP + 0.42 C140ACP + 0.007 C141ACP + 0.254 C160ACP + 0.175 C161ACP + 0.013 C180ACP + 0.112 C181ACP -> PA + ACP	KG	
	R137	ZMO1151	phosphatidate	2.7.7.41	PA + CTP <-> CDPDG + PPI	KG	
	R138	ZMO1159	phosphatidylserine synthase	2.7.8.8	CDPDG + SER -> CMP + PS	KG	
	R139	ZMO0096	CDP-diacylglycerol-glycerol-3-phosphate 3-phosphatidyltransferase	2.7.8.5	CDPDG + GL3P <-> CMP + PGP	KG	
	R140	ZMO0314	cardiolipin synthase	2.7.8.-	CDPDG + PG <-> CMP + CL	KG	
	R141		phosphatidylglycerophosphatase	3.1.3.27	PGP -> PI + PG	BC	
	R142	ZMO1160	phosphatidylserine	4.1.1.65	PS -> PE + CO2	KG	
	R143	ZMO0776	phosphatidylethanolamine N-methyltransferase	2.1.1.17	SAM + PE -> SAH + PNME	KG	
	R144				SAM + PNME -> SAH + PNDME		Robert A. Moreau *et al* (1995)
	R145				SAM + PNDME -> SAH + PC		Robert A. Moreau *et al* (1995)
	R146	ZMO0314	cardiolipin synthase	2.7.8.-		KG	
	R147	ZMO0170	glycerophosphoryl diester phosphodiesterase	3.1.4.46	G3PC -> CHO + GL3P	KG	
	R148	ZMO0170	glycerophosphoryl diester phosphodiesterase	3.1.4.46	G3PE -> ETHA + GL3P	KG	
	R149	ZMO1195	glycerol-3-phosphate acyltransferase	2.3.1.15	GL3P + ACYCOA <-> AGL3P + COA	KG	
	R150	ZMO0419	1-acyl-sn-glycerol-3-phosphate acyltransferase	2.3.1.51	AGL3P + ACYCOA -> PA + COA	KG	
	R151		aldehyde dehydrogenase	1.2.1.3	DGLYCERATE + NADPH <-> GLAL + NADP		Robert A. Moreau *et al* (1995)
Sphingolipid metabolism	R152	ZMO0972	ceramide glucosyltransferase	2.4.1.80	UDPG + ACSPG -> UDP + GLUCM	KG	
	R153	ZMO0904	beta-galactosidase	3.2.1.23	bGALbGLCM -> GLUCM + GLAC	KG	
**Nucelotide Metabolism**							
Purine metabolism	R154	ZMO1519	ribose-phosphate pyrophosphokinase	2.7.6.1	R5P + ATP <-> PRPP + AMP	KG	
	R155	ZMO1557	amidophosphoribosyltransferase	2.4.2.14	PRPP + GLN -> PPI + GLU + PRAM	KG	
	R156		AMP pyrophosphorylase	2.4.2.7	AD + PRPP -> PPI + AMP		adding reaction
	R157	ZMO0299	phosphoribosylamine-glycine ligase	6.3.4.13	PRAM + ATP + GLY <-> ADP + PI + GAR	KG	
	R158	ZMO0708	phosphoribosylglycinamide formyltransferase 1	2.1.2.2	GAR + FTHF -> THF + FGAR	KG	
	R159	ZMO0820	phosphoribosylformylglycinamidine synthase	6.3.5.3	FGAR + ATP + GLN -> GLU + ADP + PI + FGAM	KG	
	R160	ZMO0709	phosphoribosylformylglycinamidine cyclo-ligase	6.3.3.1	FGAM + ATP -> ADP + PI + AIR	KG	
	R161	ZMO1421	5-(carboxyamino)imidazole ribonucleotide synthase	6.3.4.18	AIR + CO2 + ATP <-> NCAIR + ADP + PI	KG	
	R162	ZMO1420	5-(carboxyamino)imidazole ribonucleotide mutase	5.4.99.18	NCAIR <-> CAIR	KG	
	R163	ZMO1052	phosphoribosylaminoimidazole-succinocarboxamide synthase	6.3.2.6	CAIR + ATP + ASP <-> ADP + PI + SAICAR	KG	
	R164	ZMO0662	adenyloSUCC lyase	4.3.2.2	SAICAR <-> FUM + AICAR	KG	
	R165	ZMO0027	phosphoribosylaminoimidazolecarboxamide formyltransferase AND IMP cyclohydrolase	2.1.2.3 AND 3.5.4.10	AICAR + FTHF <-> THF + PRFICA	KG	
	R166	ZMO0027	phosphoribosylaminoimidazolecarboxamide formyltransferase AND IMP cyclohydrolase	2.1.2.3 AND 3.5.4.10	PRFICA <-> IMP	KG	
	R167	ZMO0433	guanylate kinase	2.7.4.8	GMP + ATP <-> GDP + ADP	KG	
	R168	ZMO0443	ribonucleoside-diphosphate	1.17.4.1	GDP + RTHIO -> DGDP + OTHIO	KG	
	R169	ZMO1025	ribonucleoside-triphosphate reductase	1.17.4.2	GTP + RTHIO -> DGTP + OTHIO	KG	
	R170	ZMO0433	guanylate kinase	2.7.4.8	DGMP + ATP <-> DGDP + ADP	KG	
	R171	ZMO0842 or	dGTPase	3.1.5.1	DGTP -> DG + 3 PI	KG	
	R172	ZMO0985	5'-nucleotidase	3.1.3.5	DGMP <-> DG + PI	KG	
	R173	ZMO0985	5'-nucleotidase	3.1.3.5	GMP -> PI + GSN	KG	
	R174	ZMO0656	xanthine phosphoribosyltransferase	2.4.2.22	GN + PRPP -> PPI + GMP	KG	

Metabolism	Number	Gene Name	Enzyme	EC Number	Reaction	[1]Database	Ref.
	R175	ZMO1267 or ZMO1855	GMP synthase (glutamine-hydrolysing)	6.3.5.2	ATP + XMP + GLN -> AMP + PPI + GMP + GLU	KG	
	R176	ZMO0985	5'-nucleotidase	3.1.3.5	XMP -> PI + XTSN	KG	
	R177	ZMO0656	xanthine phosphoribosyltransferase	2.4.2.22	XAN + PRPP -> XMP + PPI	KG	
	R178	ZMO0939	guanine deaminase	3.5.4.3	GN -> XAN + NH3	KG	
	R179	ZMO1321	IMP dehydrogenase	1.1.1.205	IMP + NAD -> NADH + XMP	KG	
	R180	ZMO0985	5'-nucleotidase	3.1.3.5	IMP -> PI + INS	KG	
	R181	ZMO1687	adenyloSUCC synthase	6.3.4.4	GTP + IMP + ASP <-> GDP + PI + ASUC	KG	
	R182	ZMO0662	adenyloSUCC lyase	4.3.2.2	ASUC <-> FUM + AMP	KG	
	R183	ZMO0655	adenosine deaminase	3.5.4.4	ADN -> INS + NH3	KG	
	R184	ZMO0985	5'-nucleotidase	3.1.3.5	AMP -> PI + ADN	KG	
	R185		inosine phosphorylase	2.4.2.1	AD + R1P <-> PI + ADN	BC	
	R186	ZMO0538	adenylate kinase	2.7.4.3	ATP + AMP <-> 2 ADP	KG	
	R187	ZMO1025	ribonucleoside-triphosphate reductase	1.17.4.2	ATP + RTHIO -> DATP + OTHIO	KG	
	R188	ZMO0443 AND ZMO1039	ribonucleoside-diphosphate reductase alpha AND beta chain	1.17.4.1	ADP + RTHIO -> DADP + OTHIO	KG	
	R189	ZMO0538	adenylate kinase	2.7.4.3	DAMP + ATP <-> ADP + DADP	KG	
	R190	ZMO0655 or ZMO0971	adenosine deaminase	3.5.4.4	DA -> DIN + NH3	KG	
	R191	ZMO0985	5'-nucleotidase	3.1.3.5	DAMP <-> DA + PI	KG	
	R192	ZMO1687	adenyloSUCC synthase	6.3.4.4	IMP + GTP + ASP -> GDP + PI + ASUC	KG	
	R193	ZMO1267 or ZMO1855	GMP synthase (glutamine-hydrolysing)	6.3.5.2	XMP + ATP + GLN -> GLU + AMP + PPI + GMP	KG	
	R194	ZMO1041	ADP-ribose pyrophosphatase	3.6.1.13	ARIB -> AMP + R5P	KG	
	R195	ZMO1095	nucleoside-triphosphate pyrophosphatase	3.6.1.19	ITP -> IMP + PPI	KG	
	R196	ZMO1095	nucleoside-triphosphate pyrophosphatase	3.6.1.19	GTP -> GMP + PPI	KG	
	R197	ZMO0086	Guanosine 3',5'-bis(diphosphate) 3'-pyrophosphohydrolase	3.1.7.2	ppGpp <-> GDP + PPI	KG	
	R198	ZMO0403	exopolyphosphatase	3.6.1.11	pppGpp <-> ppGpp + PI	KG	
	R199	ZMO0152	pyruvate kinase	2.7.1.40	GTP + PYR <-> GDP + PEP	KG	
	R200	ZMO0152	pyruvate kinase	2.7.1.40	DGDP + PEP -> DGTP + PYR	KG	
	R201	ZMO1095	nucleoside-triphosphate pyrophosphatase	3.6.1.19	DGTP -> DGMP + PPI	KG	
	R202	ZMO1267 or ZMO1855	GMP synthase (glutamine-hydrolysing)	6.3.5.2	ATP + XMP + NH3 -> AMP + PPI + GMP	KG	
	R203	ZMO1095	nucleoside-triphosphate pyrophosphatase	3.6.1.19	XTP -> XMP + PPI	KG	
	R204	ZMO0152	pyruvate kinase	2.7.1.40	DATP + PYR <-> DADP + PEP	KG	
Pyrimidine metabolism	R205	ZMO0792 or ZMO1689	dihydroorotase	3.5.2.3	CAASP <-> DOROA	KG	
	R206	ZMO0791	aspartate carbamoyltransferase catalytic subunit	2.1.3.2	CAP + ASP -> CAASP + PI	KG	
	R207	ZMO1617 AND ZMO1618	carbamoyl-phosphate synthase large AND small subunit	6.3.5.5	2 ATP + GLN + CO2 -> 2 ADP + PI + GLU + CAP	KG	
	R208	ZMO1025	ribonucleoside-triphosphate reductase	1.17.4.2	UTP + RTHIO -> OTHIO + DUTP	KG	
	R209		nucleoside diphosphokinase	2.7.4.6	DCDP + ATP <-> DCTP + ADP	BC	
	R210	ZMO0120	dihydroorotate oxidase	1.3.3.1	DOROA + Q <-> QH2 + OROA	KG	
	R211	ZMO1707	orotate phosphoribosyltransferase	2.4.2.10	OROA + PRPP <-> PPI + OMP	KG	
	R212	ZMO0587	orotidine-5'-phosphate decarboxylase	4.1.1.23	OMP -> CO2 + UMP	KG	
	R213	ZMO1797	cytidylate kinase	2.7.4.14	UMP + ATP <-> ADP + UDP	KG	
	R214		nucleoside diphosphokinase	2.7.4.6	UDP + ATP <-> UTP + ADP	BC	
	R215		nucleoside diphosphokinase	2.7.4.6	CDP + ATP <-> CTP + ADP	BC	
	R216	ZMO0462	CTP synthase	6.3.4.2	UTP + GLN + ATP -> GLU + CTP + ADP + PI	KG	
	R217	ZMO1797	cytidylate kinase	2.7.4.14	CMP + ATP <-> ADP + CDP	KG	
	R218	ZMO1025	ribonucleoside-triphosphate reductase	1.17.4.2	CTP + RTHIO -> DCTP + OTHIO	KG	
	R219	ZMO1025	ribonucleoside-diphosphate reductase	1.17.4.1	CDP + RTHIO -> DCDP + OTHIO	KG	
	R220	ZMO1797	cytidylate kinase	2.7.4.14	DCMP + ATP <-> ADP + DCDP	KG	
	R221	ZMO1095	nucleoside-triphosphate pyrophosphatase	3.6.1.19	DUTP -> PPI + DUMP	KG	
	R222	ZMO1090	dTMP kinase	2.7.4.9	DUMP + ATP <-> DUDP + ADP	KG	
	R223	ZMO0443 AND ZMO1039	ribonucleoside-diphosphate reductase alpha AND beta chain	1.17.4.1	UDP + RTHIO -> DUDP + OTHIO	KG	
	R224	ZMO1755	thymidylate synthase	2.1.1.45	DUMP + METTHF -> DHF + DTMP	KG	
	R225	ZMO1090	dTMP kinase	2.7.4.9	DTMP + ATP <-> ADP + DTDP	KG	
	R226		nucleoside diphosphokinase	2.7.4.6	DTDP + ATP <-> DTTP + ADP	BC	
	R227	ZMO0985	5'-nucleotidase	3.1.3.5	UMP <-> PI + URI	KG	
	R228	ZMO0864	cytidine deaminase	3.5.4.5	CYTD -> URI + NH3	KG	
	R229	ZMO0985	5'-nucleotidase	3.1.3.5	CMP <-> CYTD + PI	KG	
	R230	ZMO0985	5'-nucleotidase	3.1.3.5	DCMP <-> DC + PI	KG	
	R231	ZMO0864	cytidine deaminase	3.5.4.5	DC -> NH3 + DU	KG	
	R232	ZMO0552	thymidine kinase	2.7.1.21	DU + ATP -> DUMP + ADP	KG	
	R233	ZMO0985	5'-nucleotidase	3.1.3.5	DTMP -> DT + PI	KG	
	R234	ZMO0552	thymidine kinase	2.7.1.21	DT + ATP -> ADP + DTMP	KG	
	R235	ZMO1142	thioredoxin reductase (NADPH)	1.8.1.9	OTHIO + NADPH -> RTHIO + NADP	KG	
	R236	ZMO0863	dCTP deaminase	3.5.4.13	CTP -> UTP + NH3	KG	
	R237	ZMO0462	CTP synthase	6.3.4.2	ATP + UTP + NH3 -> ADP + PI + CTP	KG	
	R238	ZMO1095	nucleoside-triphosphate pyrophosphatase	3.6.1.19	UTP -> UMP + PPI	KG	
	R239	ZMO0863	deoxycytidine triphosphate deaminase	3.5.4.13	DCTP -> DUTP + NH3	KG	

Metabolism	Number	Gene Name	Enzyme	EC Number	Reaction	[1]Database	Ref.
**Amino Acid Metabolism**							
Alanine, aspartate and glutamate	R240	ZMO0144	L-aspartate oxidase	1.4.3.16	ASP + O2 -> OA + NH3 + H2O2	KG	
	R241	ZMO0342	aspartate aminotransferase	2.6.1.1	ASP + AKG <-> OA + GLU	KG	
	R242	ZMO1682	aspartate 4-decarboxylase	4.1.1.12	ASP -> ALA + CO2	KG	
	R243		asparagine synthetase	6.3.1.1	ASP + ATP + NH3 -> ASN + AMP + PPI		adding reaction
	R244	ZMO0371 or ZMO1158	aspartate racemase	5.1.1.13	ASP -> DASP	KG	
	R245		alanine transaminase	2.6.1.2	PYR + GLU <-> AKG + ALA		adding reaction
	R246		glutamic dehydrogenase	1.4.1.3	AKG + NH3 + NADPH <-> GLU + NADP		adding reaction
Glycine, serine and threonine metabolism	R247	ZMO0059	glycerate kinase	2.7.1.31	ADP + 3PG <-> ATP + DGLYCERATE	KG	
	R248	ZMO1685	D-3-phosphoglycerate dehydrogenase	1.1.1.95	3PG + NAD -> PHP + NADH	KG	
	R249	ZMO1684	phosphoserine aminotransferase	2.6.1.52	PHP + GLU -> 3PSER + AKG	KG	
	R250	ZMO1137	phosphoserine phosphatase	3.1.3.3	3PSER -> SER + PI	KG	
	R251	ZMO0189	L-serine dehydratase	4.3.1.17	SER <-> PYR + NH3	KG	
	R252	ZMO1201	glycine	2.1.2.1	THF + SER -> METTHF + GLY	KG	
	R253	ZMO1198	5-aminolevulinate synthase	2.3.1.37	SUCCOA + GLY -> ALAV + COA + CO2	KG	
	R254	ZMO1347	threonine aldolase	4.1.2.5	THR <-> GLY + ACAL	KG	
	R255	ZMO1891	threonine synthase	4.2.3.1	PHSER -> THR + PI	KG	
	R256	ZMO1600	homoserine kinase type II	2.7.1.39	ATP + HSER -> ADP + PHSER	KG	
	R257	ZMO0483	homoserine dehydrogenase	1.1.1.3	HSER + NAD <-> ASPSA + NADH	KG	
	R258	ZMO0483	homoserine dehydrogenase	1.1.1.3	HSER + NADP <-> ASPSA + NADPH	KG	
	R259	ZMO1407	aspartate-semialdehyde dehydrogenase	1.2.1.11	BASP + NADPH -> ASPSA + PI + NADP	KG	
	R260	ZMO1653	aspartate kinase	2.7.2.4	ATP + ASP -> ADP + BASP	KG	
	R261	ZMO1883	gyoxylate AND hydroxypyruvate reductase A	1.1.1.79 AND 1.1.1.81	DGLY + NAD <-> HPYR + NADH	KG	
	R262	ZMO1883	gyoxylate AND hydroxypyruvate reductase A	1.1.1.79 AND 1.1.1.81	DGLY + NADP <-> HPYR + NADPH	KG	
	R263	ZMO1347	threonine aldolase	4.1.2.5	ATHR <-> GLY + ETH	KG	
Cysteine and methionine metabolism	R264	ZMO1730	serine O-acetyltransferase	2.3.1.30	ACCOA + SER <-> COA + ASER	KG	
	R265	ZMO0748 or ZMO0821	cysteine synthase A	2.5.1.47	ASER + H2S -> CYS + AC	KG	
	R266	ZMO0327	cystathionine beta-lyase	4.4.1.8	H2S + PYR + NH3 -> CYS	KG	
	R267	ZMO0182	denosylhomocysteinase	3.3.1.1	SAH <-> HCYS + ADN	KG	
	R268	ZMO1745	5-methyltetrahydrofolate-homocysteine methyltransferase	2.1.1.13	MTHF + HCYS <-> THF + MET	KG	
	R269	ZMO0273	S-adenosylmethionine	2.5.1.6	ATP + MET -> PI + PPI + SAM	KG	
	R270		adenosylmethionine decarboxylase	4.1.1.50	SAM <-> DSAM + CO2	BC	
	R271		spermidine synthase	2.5.1.16	PTRC + DSAM -> SPMD + 5MTA	BC	
	R272		methylthioadenosine nucleosidase	3.2.2.16	5MTA -> AD + 5MTR	BC	
	R273		methylthioribose kinase	2.7.1.100	5MTR + ATP -> 5MTRP + ADP	BC	
	R274		aldose-ketose-isomerase	5.3.1.23	5MTRP <-> 5MTR1P	BC	
	R275		methylthioribulose 1-phosphate dehydratase	4.2.1.109	5MTR1P -> DKMPP		adding reaction
	R276		acireductone synthase	3.1.3.77	DKMPP -> FORT + KMB		adding reaction
	R277				KMB + GLN -> GLU + MET		adding reaction
	R278	ZMO0189	L-serine dehydratase	4.3.1.17	SER -> 2AA	KG	
	R279	ZMO0748 or ZMO0821	cysteine synthase A	2.5.1.47	ASER + HO3S2 + RTHIO -> CYS + H2SO3 + OTHIO + AC	KG	
	R280	ZMO0327	cystathionine beta-lyase	4.4.1.8	CYST -> PYR + NH3 + TCYS	KG	
	R281	ZMO0342	aspartate aminotransferase	2.6.1.1	CYSTEATE + AKG <-> 3SPYR + GLU	KG	
	R282	ZMO0342	aspartate aminotransferase	2.6.1.1	CYS + AKG <-> MPYR + GLU	KG	
	R283	ZMO0342	aspartate aminotransferase	2.6.1.1	3SLALA + AKG -> 3SFPYR + GLU	KG	
	R284	ZMO1682	aspartate 4-decarboxylase	4.1.1.12	3SLALA -> ALA + SO2	KG	
	R285	ZMO1000	5-methyltetrahydropteroyltriglutamate-homocysteine methyltransferase	2.1.1.14	MTGLU + HCYS -> TGLU + MET	KG	
	R286	ZMO0676	methionine-gamma-lyase	4.4.1.11	MET -> MTE + NH3 + OBUT	KG	
	R287	ZMO0937	aromatic-amino-acid transaminase	2.6.1.57	KMB + GLU -> MET + AKG	KG	
Valine, leucine and isoleucine biosynthesis	R288	ZMO1275	threonine dehydratase	4.3.1.19	THR -> OBUT + NH3	KG	
	R289	ZMO0687 AND ZMO1139 AND ZMO1140	acetolactate synthase I/II/III large AND small subunit	2.2.1.6	OBUT + HTPP -> TPP + ABUT	KG	
	R290	ZMO0687 AND ZMO1139 AND ZMO1140	acetolactate synthase I/II/III large AND small subunit	2.2.1.6	ABUT <-> HMOPENT	KG	
	R291	ZMO1792	dihydroxy-acid dehydratase	4.2.1.9	DHMB -> MOBA	KG	
	R292	ZMO0687 AND ZMO1139 AND ZMO1140	acetolactate synthase I/II/III large AND small subunit	2.2.1.6	HTPP + PYR -> ACLAC + TPP	KG	

Metabolism	Number	Gene Name	Enzyme	EC Number	Reaction	[1]Database	Ref.
	R293	ZMO0903	2-isopropylmalate synthase	2.3.3.13	ACCOA + MOBA -> IPPMAL + COA	KG	
	R294	ZMO0913	branched-chain amino acid aminotransferase	2.6.1.42	MOPENA + GLU -> ILE + AKG	KG	
	R295	ZMO0913	branched-chain amino acid aminotransferase	2.6.1.42	MOBA + GLU -> VAL + AKG	KG	
	R296	ZMO0677	3-isopropylmalate dehydrogenase	1.1.1.85	IPPMAL + NAD -> OICAP + NADH	KG	
	R297	ZMO0913	branched-chain amino acid aminotransferase	2.6.1.42	OICAP + GLU <-> LEU + AKG	KG	
	R298	ZMO0903	2-isopropylmalate synthase	2.3.3.13	ACCOA + MOBA -> IPPMAL + COA	KG	
	R299	ZMO0677	3-isopropylmalate dehydrogenase	1.1.1.85	E3MM + NAD -> OBUT + CO2 + NADH	KG	
	R300	ZMO0105 AND ZMO0106	3-isopropylmalate AND (R)-2-methylmalate dehydratase large AND small subunit	4.2.1.33 AND 4.2.1.35	2MM -> E3MM	KG	
	R301	ZMO0105 AND ZMO0106	3-isopropylmalate AND (R)-2-methylmalate dehydratase large AND small subunit	4.2.1.33 AND 4.2.1.35	R2MM -> 2MM	KG	
	R302	ZMO0687 AND ZMO1139 AND ZMO1140	acetolactate synthase I/II/III large AND small subunit	2.2.1.6	HTPP + PYR -> ACLAC + TPP	KG	
	R303	ZMO0687 AND ZMO1139 AND ZMO1140	acetolactate synthase I/II/III large AND small subunit	2.2.1.6	OBUT + HTPP -> TPP + ABUT	KG	
	R304	ZMO0687 AND ZMO1139 AND ZMO1140	acetolactate synthase I/II/III large AND small subunit	2.2.1.6	ABUT <-> HMOPENT	KG	
	R305	ZMO1141	ketol-acid reductoisomerase	1.1.1.86	ACLAC <-> HMOBA	KG	
	R306	ZMO1141	ketol-acid reductoisomerase	1.1.1.86	HMOPENT + NADPH <-> DHMP + NADP	KG	
	R307	ZMO1141	ketol-acid reductoisomerase	1.1.1.86	HMOBA + NADPH <-> DHMB + NADP	KG	
	R308	ZMO1792	dihydroxy-acid dehydratase	4.2.1.9	DHMP -> MOPENA	KG	
	R309	ZMO1792	dihydroxy-acid dehydratase	4.2.1.9	DHMB -> MOBA	KG	
	R310	ZMO0913	branched-chain amino acid aminotransferase	2.6.1.42	MOBA + GLU -> VAL + AKG	KG	
	R311	ZMO0105 AND ZMO0106	3-isopropylmalate AND (R)-2-methylmalate dehydratase large AND small subunit	4.2.1.33 AND 4.2.1.35	IPPMAL <-> IPPMALE	KG	
Lysine biosynthesis	R312	ZMO0720 or ZMO1853	dihydrodipicolinate synthase	4.2.1.52	ASPSA + PYR -> D23PIC	KG	
	R313	ZMO0707	dihydrodipicolinate reductase	1.3.1.26	D23PIC + NADPH -> PIP26DX + NADP	KG	
	R314	ZMO0431	2,3,4,5-tetrahydropyridine-2-carboxylate N-	2.3.1.117	SUCCOA + PIP26DX -> COA + NS2A6O	KG	
	R315	ZMO0408	acetylornithine AND N-succinyldiaminopimelate aminotransferase	2.6.1.11 AND 2.6.1.17	NS26DP + AKG <-> NS2A6O + GLU	KG	
	R316	ZMO1632	succinyl-diaminopimelate desuccinylase	3.5.1.18	NS26DP -> SUCC + D26PIM	KG	
	R317	ZMO1072	diaminopimelate epimerase	5.1.1.7	D26PIM <-> MDAP	KG	
	R318	ZMO1768	diaminopimelate decarboxylase	4.1.1.20	MDAP -> LYS + CO2	KG	
	R319	ZMO0826	UDP-N-acetylmuramoylalanyl-D-glutamate-2,6-diaminopimelate ligase	6.3.2.13	UDPNAMAG + ATP + MDAP -> UNAGD + ADP + PI	KG	
	R320	ZMO0827	UDP-N-acetylmuramoylalanyl-D-glutamyl-2,6-diaminopimelate-D-alanyl-D-alanine ligase	6.3.2.10	UNAGD + ATP + AA -> UNAGDA + ADP + PI	KG	
	R321	ZMO1835	homocitrate synthase	2.3.3.14	ACCOA + AKG -> HCT + COA	KG	
	R322	ZMO1036	argininoSUCC synthase	6.3.4.5	ATP + CITR + ASP <-> AMP + PPI + ARGSUCC	KG	
	R323	ZMO1770	argininoSUCC lyase	4.3.2.1	ARGSUCC <-> FUM + ARG	KG	
	R324	ZMO0707	dihydrodipicolinate reductase	1.3.1.26	D23PIC + NADH -> PIP26DX + NAD	KG	
Arginine and proline metabolism	R325	ZMO0432	arginase	3.5.3.1	ARG -> ORN + UREA	KG	
	R326	ZMO0408	acetylornithine AND N-succinyldiaminopimelate aminotransferase	2.6.1.11 AND 2.6.1.17	NAGLUSAL + GLU <-> AKG + NAARON	KG	
	R327	ZMO0804	N-acetyl-gamma-glutamyl-phosphate reductase	1.2.1.38	NAGLUYP + NADPH <-> NADP + PI + NAGLUSAL	KG	
	R328	ZMO1494	acetylglutamate kinase	2.7.2.8	NAGLU + ATP -> ADP + NAGLUYP	KG	
	R329	ZMO0923	glutamate N-acetyltransferase or amino-acid N-acetyltransferase	2.3.1.35 AND 2.3.1.1	GLU + ACCOA -> COA + NAGLU	KG	
	R330	ZMO0206	glutamate 5-kinase	2.7.2.11	ATP + GLU -> ADP + GLUP	KG	
	R331	ZMO1661	glutamate-5-semialdehyde dehydrogenase	1.2.1.41	GLUP + NADPH -> GLUGSAL + PI + NADP	KG	
	R332	ZMO1020	ornithine decarboxylase	4.1.1.17	ORN -> PTRC + CO2	KG	
	R333	ZMO0311	pyrroline-5-carboxylate	1.5.1.2	PRO + NADP <-> PYCA + NADPH	KG	
	R334	ZMO0657	arginine N-succinyltransferase	2.3.1.109	SUCCOA + ARG -> COA + SUCCARG	KG	
	R335	ZMO1975	ornithine cyclodeaminase	4.3.1.12	ORN <-> PRO + NH3	KG	
	R336	ZMO0311	pyrroline-5-carboxylate	1.5.1.2	PRO + NAD <-> PYCA + NADH	KG	
	R337	ZMO1272	succinylglutamic semialdehyde dehydrogenase	1.2.1.71	SUCCGLU5SAL + NAD -> SUCCGLU + NADH	KG	
	R338	ZMO0311	pyrroline-5-carboxylate	1.5.1.2	PYRRHCAR + NADH -> HPRO + NAD	KG	
	R339	ZMO0311	pyrroline-5-carboxylate	1.5.1.2	PYRRHCAR + NADPH -> HPRO + NADP	KG	
	R340	ZMO0342	aspartate aminotransferase	2.6.1.1	E4HGLU + AKG -> HYDROXYAKG + GLU	KG	
	R341	ZMO1369	agmatine deiminase	3.5.3.12	AGMAT -> NCPTRC + NH3	KG	
	R342	ZMO1172	succinylarginine dihydrolase	3.5.3.23	SUCCARG -> SUCCORN + CO2 + 2 NH3	KG	

Metabolism	Number	Gene Name	Enzyme	EC Number	Reaction	[1]Database	Ref.
	R343	ZMO1370	N-carbamoylputrescine amidase	3.5.1.53	NCPTRC -> PTRC + CO2 + NH3	KG	
	R344	ZMO0409	ornithine transcarbamylase	2.1.3.3	CAP + ORN <-> CITR + PI	KG	
	R345	Lumped reaction			ORN + AKG <-> PYCA + GLU	KG	
	R346	ZMO0923	ornithine transacetylase	2.3.1.35	NAARON + GLU <-> ORN + NAGLU	KG	
Histidine metabolism	R347	ZMO1550 AND ZMO1686	ATP phosphoribosyltransferase AND regulatory subunit	2.4.2.17	ATP + PRPP <-> PRBATP + PPI	KG	
	R348	ZMO1499	phosphoribosyl-ATP pyrophosphohydrolase	3.6.1.31	PRBATP -> PRBAMP + PPI	KG	
	R349	ZMO1178	phosphoribosyl-AMP cyclohydrolase	3.5.4.19	PRBAMP -> PRFP	KG	
	R350	ZMO1501	phosphoribosylformimino-5-aminoimidazole carboxamide ribotide isomerase	5.3.1.16	PRFP -> PRLP	KG	
	R351	ZMO1500	cyclase HisF	4.1.3.-	PRLP + GLN -> AICAR + GLU + DIMGP	KG	
	R352	ZMO1503	imidazoleglycerol-phosphate dehydratase	4.2.1.19	DIMGP -> IMACP	KG	
	R353	ZMO0002 or ZMO0421 or ZMO0560 AND ZMO0562	histidinol-phosphate aminotransferase	2.6.1.9	IMACP + GLU -> HISOLP + AKG	KG	
	R354		histidinol-phosphatase	3.1.3.15	HISOLP -> HISOL + PI	BC	
	R355	ZMO1551	histidinol dehydrogenase	1.1.1.23	HISOL + NAD -> HISAL + NADH	KG	
	R356	ZMO1551	histidinol dehydrogenase	1.1.1.23	HISAL + NAD -> HIS + NADH	KG	
Tyrosine metabolism	R357	ZMO1236 AND ZMO1596 or ZMO1722	alcohol dehydrogenase or S-(hydroxymethyl)glutathione dehydrogenase	1.1.1.1 or 1.1.1.284	DHPEG + NAD <-> DHMA + NADH	KG	
Tryptophan metabolism	R358	ZMO1360	indolepyruvate decarboxylase	4.1.1.74	IDPYR -> IDACAL + CO2	KG	
	R359	ZMO1207	nitrilase	3.5.5.1	IDACTN -> IAC + NH3	KG	
Phenylalanine, tyrosine and tryptophan biosynthesis	R360	ZMO0187	3-deoxy-7-phosphoheptulonate synthase	2.5.1.54	PEP + E4P -> 3DDAH7P + PI	KG	
	R361	ZMO0593	3-dehydroquinate synthase	4.2.3.4	3DDAH7P -> DOT + PI	KG	
	R362	ZMO0737	3-dehydroquinate dehydratase II	4.2.1.10	DOT <-> DHSK	KG	
	R363	ZMO0041	shikimate 5-dehydrogenase	1.1.1.25	SME + NADP <-> DHSK + NADPH	KG	
	R364	ZMO0594	shikimate kinase	2.7.1.71	ATP + SME -> ADP + SME5P	KG	
	R365	ZMO1796	3-phosphoshikimate 1-carboxyvinyltransferase	2.5.1.19	PEP + SME5P <-> PI + 3PSME	KG	
	R366	ZMO1693	chorismate synthase	4.2.3.5	3PSME -> CHOR + PI	KG	
	R367	ZMO0584	tryptophan synthase alpha chain	4.2.1.20	INDOLE + T3P1 <-> IGP	KG	
	R368	ZMO0585	tryptophan synthase beta chain	4.2.1.20	SER + INDOLE -> TRP	KG	
	R369	ZMO0584 AND ZMO0585	tryptophan synthase alpha AND beta chain	4.2.1.20	SER + IGP <-> TRP + T3P1	KG	
	R370	ZMO0545	indole-3-glycerol phosphate synthase	4.1.1.48	CPAD5P -> CO2 + IGP	KG	
	R371	ZMO0586	phosphoribosylanthranilate isomerase	5.3.1.24	NPRAN -> CPAD5P	KG	
	R372	ZMO0200	anthranilate phosphoribosyltransferase	2.4.2.18	AN + PRPP -> PPI + NPRAN	KG	
	R373	ZMO0201 AND ZMO0468	anthranilate synthase component Ⅰ AND II	4.1.3.27	CHOR + NH3 -> AN + PYR	KG	
	R374	ZMO0201 AND ZMO0468	anthranilate synthase component Ⅰ AND II	4.1.3.27	CHOR + GLN -> AN + PYR + GLU	KG	
	R375	ZMO0563	chorismate mutase	5.4.99.5	CHOR <-> PHEN	KG	
	R376	ZMO1678	prephenate dehydratase	4.2.1.51	PHEN <-> PHPYR + CO2	KG	
	R377	ZMO0937	aromatic-amino-acid transaminase	2.6.1.57	PHE + AKG <-> PHPYR + GLU	KG	
	R378	ZMO0342	aspartate aminotransferase	2.6.1.1	TYR + AKG <-> HPHPYR + GLU	KG	
	R379	ZMO0937	aromatic-amino-acid transaminase	2.6.1.57	ASP + PHEN -> OA + AG	KG	
	R380	ZMO1678	prephenate dehydratase	4.2.1.51	AG -> PHE + CO2	KG	
	R381	ZMO0420	cyclohexadienyl dehydrogenase	1.3.1.43	AG + NAD -> TYR + CO2 + NADH	KG	
	R382	ZMO0420	cyclohexadienyl dehydrogenase	1.3.1.43	AG + NADP -> TYR + CO2 + NADPH	KG	
**Metabolism of Other Amino Acids**							
Selenoamino acid metabolism	R383	ZMO0327	cystathionine beta-lyase	4.4.1.8	SLLCT -> SHCYS + NH3 + PYR	KG	
	R384	ZMO0182	adenosylhomocysteinase	3.3.1.1	ADSHCYS -> ADN + SHCYS	KG	
	R385	ZMO0273	S-adenosylmethionine	2.5.1.6	ATP + SMET -> PI + PPI + ADSMET	KG	
	R386	ZMO0676	methionine-gamma-lyase	4.4.1.11	SMET -> MTSEL + NH3 + OBUT	KG	
	R387	ZMO0748 or ZMO0821	cysteine synthase A	2.5.1.47	ASER + SELD -> SCYS + AC	KG	
	R388	ZMO0004 AND ZMO0005	sulfate adenylyltransferase subunit 1 AND 2	2.7.7.4	ATP + SELNT <-> PPI + ADSELNT	KG	
	R389	ZMO0003	adenylylsulfate kinase	2.7.1.25	ATP + ADSELNT -> ADP + 3PADSELNT	KG	
Cyanoamino acid metabolism	R390	ZMO1207	nitrilase	3.5.5.1	APRCN -> ALA + NH3	KG	
	R391	ZMO1207	nitrilase	3.5.5.1	ACYABUT -> GLU + NH3	KG	
	R392	ZMO1388	gamma-glutamyltranspeptidase	2.3.2.2	CYAALA + GLU -> GLUCYALA	KG	
	R393	ZMO1388	gamma-glutamyltranspeptidase	2.3.2.2	APRCN + GLU -> GLUAPRCN	KG	
	R394	ZMO1683	L-asparaginase	3.5.1.1	ASN -> ASP + NH3	KG	

Metabolism	Number	Gene Name	Enzyme	EC Number	Reaction	[1]Database	Ref.
D-Glutamine and D-glutamate metabolism	R395	ZMO1197	glutamate racemase	5.1.1.3	GLU <-> DGLU	KG	
	R396	ZMO0829	UDP-N-acetylmuramoylalanine--D-glutamate ligase	6.3.2.9	UDPNAMA + DGLU + ATP -> UDPNAMAG + ADP + PI	KG	
	R397	ZMO0832	UDP-N-acetylmuramate--alanine ligase	6.3.2.8	UDPNAM + ALA + ATP -> ADP + PI + UDPNAMA	KG	
D-Alanine metabolism	R398	ZMO1592	alanine racemase	5.1.1.1	ALA <-> DALA	KG	
	R399	ZMO0834	D-alanine-D-alanine ligase	6.3.2.4	ATP + 2 DALA -> ADP + PI + AA	KG	
Glutathione metabolism	R400	ZMO1556	glutamate-cysteine ligase	6.3.2.2	ATP + GLU + CYS -> ADP + PI + GC	KG	
	R401	ZMO1345 or ZMO1776	aminopeptidase	3.4.11.2	GC -> CYS + GLY	BC	
	R402	ZMO1913	glutathione synthase	6.3.2.3	ATP + GC + GLY -> ADP + PI + RGT	KG	
	R403	ZMO1211	glutathione reductase (NADPH)	1.8.1.7	OGT + NADPH <-> 2 RGT + NADP	KG	
	R404	ZMO0806	leucyl aminopeptidase	3.4.11.1	CYSGLY -> CYS + GLY	KG	
	R405	ZMO1388	gamma-glutamyltranspeptidase	2.3.2.2	RGT -> CYSGLY + GLU	KG	
	R406	ZMO0544	isocitrate dehydrogenase	1.1.1.42	ICIT + NADP -> AKG + CO2 + NADPH	KG	
	R407	ZMO0935 or ZMO1118	glutathione S-transferase	2.5.1.18	RX + RGT -> HAL + RSGSH	KG	
	R408	ZMO1388	gamma-glutamyltranspeptidase	2.3.2.2	RSGSH -> RSCYSGLY + GLU	KG	
	R409	ZMO1345 or ZMO1776	aminopeptidase N	3.4.11.2	RSCYSGLY -> RSCYS + GLY	KG	
**Glycan Biosynthesis and Metabolism**							
Peptidoglycan biosynthesis	R410	ZMO0828	phospho-N-acetylmuramoyl-pentapeptide-transferase	2.7.8.13	UNAGDA + UDCPP -> UMP + PI + UAGMDA	KG	
	R411	ZMO0831	UDP-N-acetylglucosamine--N acetylmuramyl-(pentapeptide) pyrophosphoryl-undecaprenol N-acetylglucosamine transferase	2.4.1.227	UAGMDA + UDPNAG -> UDP + UAAGMDA	KG	
	R412	Peptigoglycan synthesis (lumped reaction)			UAAGMDA -> UDCPDP + PEPTIDO	KG	
	R413	ZMO1115	undecaprenyl-diphosphatase	3.6.1.27	UDCPDP -> UDCPP + PI	KG	
	R414	Peptigoglycan synthesis (lumped reaction)			PPEPTIDO + DALA -> PEPTIDO + DALAxt	KG	
**Metabolism of Cofactors and Vitamins**							
Thiamine metabolism	R415	ZMO0172	thiamine biosynthesis protein ThiC		AIR -> AHM	KG	
	R416	ZMO0332 or ZMO1425	thiamine-phosphate pyrophosphorylase	2.5.1.3	THZP + AHMPP -> THMP + PPI	KG	
	R417	ZMO1553	thiamine-monophosphate kinase	2.7.4.16	THMP + ATP <-> TPP + ADP	KG	
	R418	ZMO1003	phosphomethylpyrimidine kinase	2.7.4.7	ATP + AMPMPM -> ADP + AHMPP	KG	
	R419	ZMO0172	thiamine biosynthesis protein ThiC		C15815 + DX5P + TYR -> THZP	KG	
	R420	ZMO0172	thiamine biosynthesis protein ThiC		C15815 + TYR + IMGLY -> THZP	KG	
	R421	ZMO0013	nucleoside-triphosphatase	3.6.1.15	THMPP -> THMP + PI	KG	
Riboflavin metabolism	R422	ZMO0474 or ZMO1698	GTP cyclohydrolase II or 3,4-dihydroxy 2-butanone 4-phosphate synthase	3.5.4.25 or 4.1.99.12	GTP -> FORT + D6RP5P + PPI	KG	
	R423	ZMO0476	diaminohydroxyphosphoribosylaminopyrimidine deaminase or 5-amino-6-(5-phosphoribosylamino)uracil reductase	3.5.4.26 or 1.1.1.193	D6RP5P -> A6RP5P + NH3	KG	
	R424	ZMO0476	diaminohydroxyphosphoribosylaminopyrimidine deaminase or 5-amino-6-(5-phosphoribosylamino)uracil reductase	3.5.4.26 or 1.1.1.193	A6RP5P + NADPH -> A6RP5P2 + NADP	KG	
	R425		Hydrolases	3.1.3.-	A6RP5P2 -> A6RP + PI		adding reaction
	R426	ZMO0474 or ZMO1698	GTP cyclohydrolase II or 3,4-dihydroxy 2-butanone 4-phosphate synthase	3.5.4.25 or 4.1.99.12	RL5P -> DB4P + FORT	KG	
	R427	ZMO0473	riboflavin synthase beta chain	2.5.1.-	DB4P + A6RP -> D8RL + PI	KG	
	R428	ZMO0475	riboflavin synthase alpha chain	2.5.1.9	2 D8RL -> RIBFLV + A6RP	KG	
	R429	ZMO0322	riboflavin kinase or FMN adenylyltransferase	2.7.1.26 or 2.7.7.2	RIBFLV + ATP -> ADP + FMN	KG	
	R430	ZMO0322	riboflavin kinase or FMN adenylyltransferase	2.7.1.26 or 2.7.7.2	FMN + ATP -> PPI + FAD	KG	
	R431	ZMO0061	4-phytase or acid phosphatase	3.1.3.26 or 3.1.3.2	FMN -> RIBFLV + PI	KG	
Vitamin B6 metabolism	R432	ZMO0851	pyridoxamine 5'-phosphate oxidase	1.4.3.5	PL + O2 + NH3 <-> PDLA + H2O2	KG	
	R433	ZMO0851	pyridoxamine 5'-phosphate oxidase	1.4.3.5	PDLA5P + O2 <-> PL5P + H2O2 + NH3	KG	
	R434	ZMO0851	pyridoxamine 5'-phosphate oxidase	1.4.3.5	PYRDX + O2 <-> PL + H2O2	KG	
	R435	ZMO0851	pyridoxamine 5'-phosphate oxidase	1.4.3.5	P5P + O2 <-> PL5P + H2O2	KG	
	R436	ZMO1684	phosphoserine aminotransferase	2.6.1.52	OHB + GLU <-> PHT + AKG	KG	
	R437	ZMO1891	threonine synthase	4.2.3.1	PHT -> 4HLT + PI	KG	
	R438	ZMO1708	pyridoxine 5-phosphate synthase	2.6.99.2	AOPP + DX5P -> P5P + PI	KG	

Metabolism	Number	Gene Name	Enzyme	EC Number	Reaction	[1]Database	Ref.
	R439	ZMO1313	4-hydroxythreonine-4-phosphate dehydrogenase	1.1.1.262	PHT + NAD -> AOPOB + NADH	KG	
Nicotinate and nicotinamide metabolism	R440	ZMO1329	NAD+ kinase	2.7.1.23	ATP + NAD -> ADP + NADP	KG	
	R441		NAD+ pyrophosphorylase	2.7.7.1 or 2.7.7.18	NAMN + ATP <-> PPI + NAAD	BC	
	R442	ZMO1662	nicotinate-nucleotide adenylyltransferase	2.7.7.18	NMN + ATP <-> PPI + NAD	KG	
	R443	ZMO0899	NAD+ synthase	6.3.1.5	ATP + NAAD + NH3 -> AMP + PPI + NAD	KG	
	R444	ZMO1870	nicotinate-nucleotide pyrophosphorylase (carboxylating)	2.4.2.19	QA + PRPP -> NAMN + CO2 + PPI	KG	
	R445	ZMO1871	quinolinate synthase	2.5.1.72	ISUCC + T3P2 -> PI + QA	KG	
	R446	ZMO1329	NAD+ kinase	2.7.1.23	NAD + ATP -> ADP + NADP	KG	
	R447	ZMO0985	5'-nucleotidase	3.1.3.5	NMN -> PI + RNAM	KG	
	R448	ZMO0985	5'-nucleotidase	3.1.3.5	NAMN -> PI + NAMNS	KG	
	R449	ZMO0144	L-aspartate oxidase	1.4.3.16	ASP + O2 -> ISUCC + H2O2	KG	
Pantothenate and CoA biosynthesis	R450	ZMO1141	ketol-acid reductoisomerase	1.1.1.86	ACLAC + NADPH <-> MOBA + NADP	KG	
	R451	ZMO1952 or ZMO1970	3-methyl-2-oxobutanoate hydroxymethyltransferase	2.1.2.11	MOBA + METTHF -> AKP + THF	KG	
	R452		2-oxopantoate reductase	1.1.1.169	AKP + NADPH -> NADP + PANT	BC	
	R453	ZMO1971	pantoate--beta-alanine ligase	6.3.2.1	PANT + bALA + ATP -> AMP + PPI + PNTO	KG	
	R454		aspartate alpha-decarboxylase	4.1.1.11	ASP -> CO2 + bALA		adding reaction
	R455	ZMO1867	type III pantothenate kinase	2.7.1.33	PNTO + ATP -> ADP + 4PPNTO	KG	
	R456	ZMO1190	phosphopantothenoylcysteine decarboxylase or phosphopantothenate--cysteine ligase	4.1.1.36 or 6.3.2.5	4PPNTO + CTP + CYS -> CMP + PPI + 4PPNCYS	KG	
	R457	ZMO1190	phosphopantothenoylcysteine decarboxylase or phosphopantothenate--cysteine ligase	4.1.1.36 or 6.3.2.5	4PPNCYS -> CO2 + 4PPNTE	KG	
	R458	ZMO1709	holo-[acyl-carrier protein] synthase	2.7.8.7	COA -> PAP + ACP	KG	
	R459	ZMO0854	pantetheine-phosphate adenylyltransferase	2.7.7.3	4PPNTE + ATP -> PPI + DPCOA	KG	
	R460	ZMO0040	dephospho-CoA kinase	2.7.1.24	DPCOA + ATP -> ADP + COA	KG	
	R461	ZMO1190	phosphopantothenoylcysteine decarboxylase or phosphopantothenate--cysteine ligase	4.1.1.36 or 6.3.2.5	ATP + 4PPNTO + CYS -> AMP + PPI + 4PPNCYS	KG	
	R462	ZMO1867	type III pantothenate kinase	2.7.1.33	ATP + PNCYS -> ADP + 4PPNCYS	KG	
	R463	ZMO1867	type III pantothenate kinase	2.7.1.33	ATP + PTT -> ADP + 4PPNTE	KG	
Biotin metabolism	R464	ZMO1917	8-amino-7-oxononanoate synthase	2.3.1.47	ALA + CHCOA <-> CO2 + COA + AONA	KG	
	R465	ZMO1918	adenosylmethionine-8-amino-7-oxononanoate aminotransferase	2.6.1.62	SAM + AONA <-> SAMOB + DANNA	KG	
	R466	ZMO1915	dethiobiotin synthetase	6.3.3.3	CO2 + DANNA + ATP <-> DTB + PI + ADP	KG	
	R467	ZMO0094	biotin synthetase	2.8.1.6	DTB + S + 2 SAM -> BT + 2 MET + 2 DA	KG	
	R468	ZMO1868	BirA family transcriptional regulator, biotin operon repressor or biotin-[acetyl-CoA-carboxylase] ligase	6.3.4.15	ATP + BT -> PPI + B5AMP	KG	
Folate biosynthesis	R469	ZMO0938	alkaline phosphatase	3.1.3.1	AHTD -> DHP + 3 PI	KG	
	R470	ZMO1059	dihydroneopterin aldolase	4.1.2.25	DHP -> AHHMP + GLAL	KG	
	R471	ZMO1647	2-amino-4-hydroxy-6-hydroxymethyldihydropteridine pyrophosphokinase	2.7.6.3	AHHMP + ATP -> AMP + AHHMD	KG	
	R472	ZMO1006	dihydropteroate synthase	2.5.1.15	AHHMP + PABA -> DHPT	KG	
	R473	ZMO1006	dihydropteroate synthase	2.5.1.15	AHHMD + PABA -> PPI + DHPT	KG	
	R474	ZMO0582	dihydrofolate synthase or folylpolyglutamate synthase	6.3.2.12 or 6.3.2.17	DHPT + ATP + GLU -> ADP + PI + DHF	KG	
	R475	ZMO0113 AND ZMO0114	para-aminobenzoate synthetase component I AND Ⅱ	2.6.1.85	CHOR + GLN -> ADCHOR + GLU	KG	
	R476	ZMO0321	dihydrofolate reductase	1.5.1.3	DHF + NADPH <-> NADP + THF	KG	
	R477		aminodeoxychorismate lyase	4.1.3.38	ADCHOR -> PYR + PABA	BC	
	R478	ZMO1229	GTP cyclohydrolase I	3.5.4.16	GTP -> FAPNTP	KG	
	R479	ZMO1229	GTP cyclohydrolase I	3.5.4.16	FAPNTP -> DAPMNTP + FORT	KG	
	R480	ZMO1229	GTP cyclohydrolase I	3.5.4.16	DAPMNTP -> AHTD	KG	
	R481	ZMO1229	GTP cyclohydrolase I	3.5.4.16	DATPTHOPAOPM -> AHTD	KG	
	R482	ZMO0818	6-pyruvoyl tetrahydrobiopterin synthase	4.2.3.12	AHTD -> PYTHP + PPPI	KG	
	R483	ZMO0321	dihydrofolate reductase	1.5.1.3	DHF + NAD <-> FL + NADH	KG	
	R484	ZMO0321	dihydrofolate reductase	1.5.1.3	DHF + NADP <-> FL + NADPH	KG	
	R485	ZMO0321	dihydrofolate reductase	1.5.1.3	FL + 2 NADH -> THF + 2 NAD	KG	
	R486	ZMO0321	dihydrofolate reductase	1.5.1.3	FL + 2 NADPH -> THF + 2 NADP	KG	
One carbon pool by folate	R487	ZMO0914	methylenetetrahydrofolate dehydrogenase (NADP+) or methenyltetrahydrofolate cyclohydrolase	1.5.1.5 or 3.5.4.9	FTHF <-> METHF	KG	
	R488	ZMO0914	methylenetetrahydrofolate dehydrogenase (NADP+) or methenyltetrahydrofolate cyclohydrolase	1.5.1.5 or 3.5.4.9	METHF + NADPH <-> METTHF + NADP	KG	
	R489	ZMO0454	formate--tetrahydrofolate ligase	6.3.4.3	THF + FORT + ATP -> ADP + PI + FTHF	KG	
	R490	ZMO0321	dihydrofolate reductase	1.5.1.3	DHF + NADH -> THF + NAD	KG	
	R491	ZMO0708	phosphoribosylglycinamide formyltransferase 1	2.1.2.2	GAR + METHF -> FGAR + THF	KG	
	R492	ZMO0215	5-formyltetrahydrofolate cyclo-ligase	6.3.3.2	ATP + FTHF -> ADP + PI + METHF	KG	

Metabolism	Number	Gene Name	Enzyme	EC Number	Reaction	[1]Database	Ref.
Porphyrin and chlorophyll metabolism	R493	ZMO1903	hydroxymethylbilane synthase	2.5.1.61	4 PBG -> HMB + 4 NH3	KG	
	R494	ZMO1902	uroporphyrinogen-III synthase	4.2.1.75	HMB -> UPRG	KG	
	R495	ZMO0006 or ZMO1271	uroporphyrin-III C-methyltransferase or precorrin-2 dehydrogenase or sirohydrochlorin ferrochelatase	2.1.1.107 or 1.3.1.76 or 4.99.1.4	2 SAM + UPRG -> 2 SAH + PC2	KG	
	R496	ZMO1998	uroporphyrinogen decarboxylase	4.1.1.37	UPRG -> 4 CO2 + CPP	KG	
	R497	ZMO0951	coproporphyrinogen III oxidase	1.3.3.3	O2 + CPP -> 2 CO2 + PPHG	KG	
	R498	ZMO0303	ferrochelatase	4.99.1.1	PPIX -> PTH	KG	
	R499	ZMO1879	porphobilinogen synthase	4.2.1.24	2 ALAV -> PBG	KG	
	R500	ZMO0006 or ZMO1271	uroporphyrin-III C-methyltransferase or precorrin-2 dehydrogenase or sirohydrochlorin ferrochelatase	2.1.1.107 or 1.3.1.76 or 4.99.1.4	SHCL + Fe -> SHEME	KG	
	R501	ZMO1998	uroporphyrinogen decarboxylase	4.1.1.37	UPRG -> CPP + 4 CO2	KG	
	R502	ZMO0006 or ZMO1271	uroporphyrin-III C-methyltransferase or precorrin-2 dehydrogenase or sirohydrochlorin ferrochelatase	2.1.1.107 or 1.3.1.76 or 4.99.1.4	PC2 + NAD -> SHCL + NADH	KG	
	R503	ZMO0012 or ZMO1364	oxygen-independent coproporphyrinogen III oxidase	1.3.99.22	CPP + 2 SAM -> PPHG + 2 CO2 + 2 MET + 2 DA	KG	
Ubiquinone and other terpenoid-quinone biosynthesis	R504	ZMO1419	4-hydroxybenzoate octaprenyltransferase	2.5.1.-	4HBZ + OPP -> O4HBZ + PPI	KG	
	R505		Octaprenyl-hydroxybenzoate decarboxylase	4.1.1.-	O4HBZ -> CO2 + 2OPPP		adding reaction
	R506	ZMO1189	ubiquinone biosynthesis protein		2OPPP + O2 + NADPH -> 2O6H + NADP	KG	
	R507	ZMO1654	3-demethylubiquinone-9 3-methyltransferase	2.1.1.- or 2.1.1.64	2O6H + SAM -> 2OPMP + SAH	KG	
	R508	ZMO1703	2-octaprenyl-6-methoxyphenol hydroxylase	1.14.13.-	2OPMP + O2 + NADPH -> 2OPMB + NADP	KG	
	R509	ZMO1188	ubiquinone or menaquinone biosynthesis methyltransferase	2.1.1.-	2OPMB + SAM -> 2OPMMB + SAH	KG	
	R510	ZMO1669	ubiquinone biosynthesis monooxygenase Coq7	1.14.13.-	2OPMMB + O2 + NADPH -> 2OMHMB + NADP	KG	
	R511	ZMO1654	3-demethylubiquinone-9 3-methyltransferase	2.1.1.- or 2.1.1.64	2OMHMB + SAM -> QH2 + SAH	KG	
	R512	ZMO1419	4-hydroxybenzoate octaprenyltransferase	2.5.1.-	PPPP + 4HB -> H3PPBZ + PPI	KG	
	R513	ZMO1189	ubiquinone biosynthesis protein		PPPH + O2 + NADPH -> PP6HPH + NADP	KG	
	R514	ZMO1654	3-demethylubiquinone-9 3-methyltransferase	2.1.1.- or 2.1.1.64	PP6HPH + SAM -> PP6MPH + SAH	KG	
	R515	ZMO1703	2-octaprenyl-6-methoxyphenol hydroxylase	1.14.13.-	PP6MPH + O2 -> PP6M14BQ	KG	
	R516	ZMO1188	ubiquinone or menaquinone biosynthesis methyltransferase	2.1.1.-	PP6M14BQ + SAM -> PP3M6M14BQ + SAH	KG	
	R517	ZMO1669	ubiquinone biosynthesis monooxygenase Coq7	1.14.13.-	PP3M6M14BQ + O2 + NADPH -> PP3M5H6M14BQ + NADP	KG	
	R518	ZMO1654	3-demethylubiquinone-9 3-methyltransferase	2.1.1.- or 2.1.1.64	PP3M5H6M14BQ + SAM -> Q + SAH	KG	
	R519	ZMO1188	ubiquinone or menaquinone biosynthesis methyltransferase	2.1.1.-	DMQ + SAM -> MQ + SAH	KG	
	R520	ZMO1188	ubiquinone or menaquinone biosynthesis methyltransferase	2.1.1.-	2P14NQ + SAM -> PQ + SAH	KG	
**Biosynthesis of Polyketides and Terpenoids**							
Biosynthesis of siderophore group nonribosomal peptides	R521	ZMO0758 or ZMO1887	enterobactin isochorismatase	3.3.2.1	ICHOR -> DHDHBZ + PYR	KG	
Terpenoid backbone biosynthesis	R522	ZMO1234 or ZMO1598	1-deoxy-D-xylulose-5-phosphate synthase	2.2.1.7	PYR + T3P1 -> DX5P + CO2	KG	
	R523	ZMO1150	1-deoxy-D-xylulose-5-phosphate reductoisomerase	1.1.1.267	DX5P + NADPH -> MDE4P + NADP	KG	
	R524	ZMO1128	2-C-methyl-D-erythritol 4-phosphate cytidylyltransferase or 2-C-methyl-D-erythritol 2,4-cyclodiphosphate synthase	2.7.7.60 or 4.6.1.12	MDE4P + CTP -> CDPMDE + PPI	KG	
	R525	ZMO1182	4-diphosphocytidyl-2-C-methyl-D-erythritol kinase	2.7.1.148	CDPMDE + ATP -> 2PCDPMDE + ADP	KG	
	R526	ZMO1128	2-C-methyl-D-erythritol 4-phosphate cytidylyltransferase or 2-C-methyl-D-erythritol 2,4-cyclodiphosphate synthase	2.7.7.60 or 4.6.1.12	2PCDPMDE -> MDECPP + CMP	KG	
	R527				MDECPP + NADH -> NAD + HMB4PP		adding reaction
	R528	ZMO0875	4-hydroxy-3-methylbut-2-enyl diphosphate reductase	1.17.1.2	HMB4PP + NADH -> NAD + IPPP	KG	
	R529		isopentenyl-diphosphate Delta-isomerase	5.3.3.2	IPPP -> DMPP		adding reaction
	R530	ZMO0855	geranyltranstransferase	2.5.1.10	DMPP + IPPP -> GPP + PPI	KG	
	R531	ZMO0855	geranyltranstransferase	2.5.1.10	GPP + IPPP -> FPP + PPI	KG	
	R532		squalene synthase	2.5.1.21	2 FPP + NADPH -> SQL + 2 PPI + NADP		M. A. Hermans *et al* (1991)
	R533		Isomerases	5.4.99.17	SQL -> 5 HOPENE		M. A. Hermans *et al* (1991)
	R534		Isomerases	5.4.99.17	SQL -> HOPANOL		M. A. Hermans *et al* (1991)
	R535				HOPENE -> THBH		M. A. Hermans *et al* (1991)

Metabolism	Number	Gene Name	Enzyme	EC Number	Reaction	[1]Database	Ref.
	R536				THBH + UDPNAG -> THBHGA + THBHET		M. A. Hermans *et al* (1991)
	R537				THBHGA <-> THBHET		M. A. Hermans *et al* (1991)
	R538	ZMO0180	(E)-4-hydroxy-3-methylbut-2-enyl-diphosphate synthase	1.17.7.1	MDECPP + 2 RFD -> HMB4PP + 2 OFD	KG	
	R539	ZMO0875	4-hydroxy-3-methylbut-2-enyl diphosphate reductase	1.17.1.2	HMB4PP + NADPH -> NADP + IPPP	KG	
	R540	ZMO0875	4-hydroxy-3-methylbut-2-enyl diphosphate reductase	1.17.1.2	HMB4PP + NADH -> NAD + DMPP	KG	
	R541	ZMO0875	4-hydroxy-3-methylbut-2-enyl diphosphate reductase	1.17.1.2	HMB4PP + NADPH -> NADP + DMPP	KG	
	R542	ZMO1152	undecaprenyl pyrophosphate synthetase	2.5.1.31	FPP + IPP -> GGPP + PPI	KG	
	R543	ZMO1152	undecaprenyl pyrophosphate synthetase	2.5.1.31	GGPP + 7 IPP -> UDCPP + 7 PPI	KG	
	R544	ZMO0564	octaprenyl-diphosphate synthase	2.5.1.-	HEPPP + IPP -> OPPPI + PPI	KG	
**Biosynthesis of Other Secondary Metabolites**							
Streptomycin biosynthesis	R545		myo-inositol-1-phosphate synthase	5.5.1.4	G6P -> I3P		adding reaction
Novobiocin biosynthesis	R546	ZMO0420	cyclohexadienyl dehydrogenase	1.3.1.43	PHEN + NAD <-> HPHPYR + CO2 + NADH	KG	
**Xenobiotics Biodegradation and Metabolism**							
	R547	ZMO1351 or ZMO1992	carboxymethylenebutenolidase	3.1.1.45	DCLCMBO -> DCLOHE	KG	
	R548	ZMO1351 or ZMO1992	carboxymethylenebutenolidase	3.1.1.45	CMBO -> 2MAC	KG	
	R549	ZMO0061 or ZMO0130	4-phytase or acid phosphatase (class A)	3.1.3.26 or 3.1.3.2	NPHP -> NPH + PI	KG	
	R550	ZMO1236 or ZMO1596 or ZMO1722	alcohol dehydrogenase or S-(hydroxymethyl)glutathione dehydrogenase	1.1.1.1 or 1.1.1.284	CLPOL + NAD -> CLAALD + NADH	KG	
	R551	ZMO0456	vanillate monooxygenase	1.14.13.82	VAN + O2 + NADH -> DHBZ + NAD + FALD	KG	
	R552	ZMO0893	esterase or lipase	3.1.1.-	SULLAC -> HSO3 + 2MAC	KG	
	R553	ZMO1823 AND ZMO1824 AND ZMO1825	nitrogenase iron protein NifH AND nitrogenase molybdenum-iron protein alpha AND beta chain	1.18.6.1	HC2H + RFD + ATP -> C2H4 + OFD + ADP + PI	KG	
	R554	ZMO1771		1.1.1.-	EO + COA + NAD -> ACCOA + NADH	KG	
	R555	ZMO1351 or ZMO1992	carboxymethylenebutenolidase	3.1.1.45	CLCMBO -> CLMAC	KG	
	R556	ZMO1351 or ZMO1992	carboxymethylenebutenolidase	3.1.1.45	PAMN -> ACAC	KG	
	R557	ZMO1207	nitrilase	3.5.5.1	BZNIT -> BZ + NH3	KG	
	R558	ZMO0053	3-oxoadipate enol-lactonase	3.1.1.24	ODHFAC -> OAP	KG	
	R559	ZMO1236 or ZMO1596 or ZMO1722	alcohol dehydrogenase or S-(hydroxymethyl)glutathione dehydrogenase	1.1.1.1 or 1.1.1.284	HMNAPTH + NAD -> NAPTHAH + NADH	KG	
	R560	ZMO1236 or ZMO1596 or ZMO1722	alcohol dehydrogenase or S-(hydroxymethyl)glutathione dehydrogenase	1.1.1.1 or 1.1.1.284	NAPTHM + NAD -> NAPTHAH + NADH	KG	
	R561	ZMO1351 or ZMO1992	carboxymethylenebutenolidase	3.1.1.45	FMUCLAC -> 2MAC + HFA	KG	
**Membrane Transport**							
	R562				NH3xt + HEXT <-> NH3	TP, TC	
	R563				Kxt + HEXT <-> K	TP, TC	
	R564				SLFxt + ATP -> SLF + ADP + PI	TP, TC	
	R565				PIxt + ATP -> ADP + 2 PI	TP, TC	
	R566				SUCCxt + HEXT <-> SUCC	TP, TC	
	R567				FUMxt + HEXT <-> FUM	TP, TC	
	R568				MALxt + HEXT <-> MAL	TP, TC	
	R569				NAxt <-> NA + HEXT	TP, TC	
	R570				CO2xt <-> CO2	TP, TC	
	R571	ZMO0013	nucleoside-triphosphatase	3.6.1.15	ATP -> ADP + PI	KG, TP, TC	
	R572				SOBxt <-> SOB + HEXT	TP, TC	
	R573				SOT <-> SOTxt + HEXT	TP, TC	
	R574				AC <-> ACxt + HEXT	TP, TC	
	R575				LAC <-> LACxt + HEXT	TP, TC	
	R576				FORT <-> FORTxt	TP, TC	
	R577				GLCxt -> GLC	TP, TC	
	R578				LEVAN <-> LEVANxt	TP, TC	
	R579				ACTN <-> ACTNxt + HEXT	TP, TC	
	R580				NADxt -> NMNxt + AMPxt	TP, TC	
	R581				NMNxt -> NMN	TP, TC	
	R582				NMNxt -> R5P + NAM	TP, TC	
	R583				NACxt -> NAC	TP, TC	
	R584				ETH -> ETHxt + HEXT	TP, TC	
	R585				O2xt <-> O2	TP, TC	
	R586				SUCxt -> SUC	TP, TC	
	R587				ACALxt <-> ACAL + HEXT	TP, TC	
	R588				GLxt <-> GL	TP, TC	
	R589				DHACTxt <-> DHACT	TP, TC	
	R590				PNTOxt + HEXT <-> PNTO	TP, TC	
	R591				FRUxt <-> FRU	TP, TC	
	R592		DNA		0.869 DATP + 0.75 DCTP + 0.869 DTTP + 0.75 DGTP + 4.4 ATP -> 4.4 ADP + 4.4 PI + 3.237 PPI + DNA		Seo *et al* (2005)
	R593		RNA		0.667 ATP + 0.929 GTP + 0.718 CTP + 0.786 UTP -> 1.24 ADP + 1.24 PI + RNA + 3.1 PPI		Neidhardt *et al* (1990)

Metabolism	Number	Gene Name	Enzyme	EC Number	Reaction	[1]Database	Ref.
	R594		Protein		2.144 ALA + 0.317 ARG + 0.435 ASN + 0.435 ASP + 0.037 CYS + 0.308 GLN + 0.307 GLU + 1.903 GLY + 0.146 HIS + 0.672 ILE + 0.672 LEU + 0.447 LYS + 0.145 MET + 0.019 PHE + 0.391 PRO + 0.383 SER + 0.415 THR + 0.093 TRP + 0.122 TYR + 1.057 VAL + 44.92 ATP -> 44.92 ADP + 44.92 PI + PROTEIN		A. A. De Graaf *et al* (1999)
	R595		Phospholipid		0.118 CL + 0.655 PE + 0.054 PG + 0.141 PINSTOL + 0.25 PC -> PHOSPHOLIPID		Robert A. Moreau *et al* (1995)
	R596		Phospholipid		GL3P + 0.1 C140ACP + 0.1 C160ACP + 0.02 C161ACP + 0.78 C181ACP -> AGL3P + ACP		Robert A. Moreau *et al* (1995)
	R597		Phospholipid		AGL3P + 0.1 C140ACP + 0.1 C160ACP + 0.02 C161ACP + 0.78 C181ACP -> PA + ACP		Robert A. Moreau *et al* (1995)
	R598		Hopanoids		0.11 THBH + 0.692 THBHGA + 0.579 THBHET + 0.07 HOPANOL + 0.024 HOPENE -> HOPANOIDS		M. A. Hermans *et al* (1991)
	R599		TAGs		1.235 GL3P + 0.408 C120ACP + 0.445 C140ACP + 0.222 C141ACP + 0.556 C160ACP + 0.593 C161ACP + 0.111 C180ACP + 1.112 C181ACP + 0.259 C190ACP -> TAG + 3.705 ACP + 1.235 PI		V. C. Carey and L. O. Ingram (1983)
	R600		Smallpool		0.167 NAD + 0.149 NADP + 0.145 COA + 0.01 ACP + 1.26 PTRC + 0.765 SPMD + 0.249 THF + 0.243 FMN + 0.141 FAD -> SMALL_MOLECULES		J Swings and J De Ley (1977) and A. A. De Graaf *et al* (1999)
	R601		Biomass		0.195 RNA + 0.027 DNA + 0.605 PROTEIN + 0.053 PHOSPHOLIPID + 0.025 PEPTIDO + 0.025 GLYCOGEN + 0.038 SMALL_MOLECULES + 16.45 ATP + 0.028 HOPANOIDS + 0.004 TAG -> Biomass + 16.45 ADP + 16.45 PI		J Swings and J De Ley (1977), A. A. De Graaf *et al* (1999), and Robert A. Moreau *et al* (1997)

These reactions are inserted to metabolize pentose sugar

Xylose			xylose isomerase	5.3.1.5.	XYL <-> XYLU		Zhang M *et al* (1995)
			xylulokinase	2.7.1.17	ATP + XYLU <-> ADP + X5P		Zhang M *et al* (1995)
Arabinose			arabinose isomerase	5.3.1.4	ARA <-> RIB		Deanda K *et al* (1996)
			ribulokinase	2.7.1.16	ATP + RIB <-> ADP + LRL5P		Deanda K *et al* (1996)
			ribulose-5-phosphate-4-epimerase	5.1.3.4	LRL5P <-> X5P		Deanda K *et al* (1996)
			transaldolase	2.2.1.2	S7P + T3P1 <-> E4P + F6P		Zhang M *et al* (1995) and Deanda K *et al* (1996)

[1] KG: KEGG, TP: TransportDB, TC: TCDB, BC: BioCyc

References

Yang *et al*., Improved genome annotation for *Zymomonas mobilis*. *Nat Biotechnol.* October 2009; 27(10): 893–894.

Tsantili IC *et al*., Quantifying the metabolic capabilities of engineered *Zymomonas mobilis* using linear programming analysis. *Microb Cell Fact.* March 2007; 6:8.

L. O. Ingram *et al*., Ethanol production by *Escherichia coli* stains co-expressing *Zymomonas PDC* and *ADH* gene. USA Patent 5000000, 1991.

Seo *et al*., The genome sequence of the ethanologenic bacterium *Zymomonas mobilis* ZM4. *Nat Biotechnol.* December 2005; 23(1):63-68

Kim *et al*., A novel aerobic respiratory chain-linked NADH oxidase system in *Zymomonas mobilis*. *J Bacteriol*. June 1995; 177(17):5176-5178.

Robert A. Moreau *et al*, Analysis of Intact Hopanoids and Other Lipids from the Bacterium *Zymomonas Mobilis* by High-Performance Liquid Chromatography. *Anal Biochem*. January 1995; 224(1): 293-301.

M. A. Hermans *et al*, Content and composition of hopanoids in *Zymomonas mobilis* under various growth conditions. *J Bacteriol*. September 1991; 173(17): 5592-5595.

Neidhardt FC *et al*., Physiology of the bacterial cell: a molecular approach. Sinauer Associates, Sunderland, Mass 1990

A. A. De Graaf *et al*, Metabolic state of *Zymomonas mobilis* in glucose-, fructose-, and xylose-fed continuous cultures as analysed by ^{13}C- and ^{31}P-NMR spectroscopy. *Arch Microbiol*. May 1999; 171(6): 371-385.

V. C. Carey and L. O. Ingram, Lipid composition of *Zymomonas mobilis* : effects of ethanol and glucose. *J Bacteriol.* June 1983; 154(3): 1291-1300.

J Swings and J De Ley, The Biology of *Zymomonas. Bacteriol Rev*. March 1977; 41(1): 1–46.

Zhang M *et al*., Metabolic engineering of a pentose metabolism pathway in ethanologenic *Zymomonas mobilis*. *Science*. January 1995; 267:240-243

Deanda K *et al*., Development of an arabinose-fermenting *Zymomonas mobilis* strain by metabolic pathway engineering. *Appl Environ Microbiol*. December 1996; 62(12):4465-4470.
