# Supplementary material for: The genome-scale metabolic network analysis of Zymomonas mobilis ZM4 explains physiological features and suggests ethanol and succinic acid production strategies
Source: Microb Cell Fact. 2010 Nov 24;9:94. doi: 10.1186/1475-2859-9-94 (PMC3004842; doi:10.1186/1475-2859-9-94)
Supplement: Additional file 2 — List of metabolites in the genome-scale metabolic model of Zymomonas mobilis ZM4 [file 1475-2859-9-94-S2.PDF]

Additional file 2. List of metabolites in the genome-scale metabolic model of *Zymomonas mobilis* ZM4

Abbreviation	[1]Metabolite
13DPG	1,3-bis-Phosphoglycerate
1BOH	1-Butanol
2AA	2-Aminoacrylate
2HETHPP	2-(alpha-Hydroxyethyl)thiamine diphosphate
2KD6PG	2-keto-3-deoxy-6-phospho-gluconate
2kGLUC	2-Keto-D-gluconic acid; 2-Dehydro-D-gluconate; 2-Dehydro-D-gluconic acid
2MAC	2-Maleylacetate
2MM	2-Methylmaleate
2O6H	2-Octaprenol 6-hydroxyphenol
2OMHMB	2-Octaprenyl-3-methyl-5-hydroxy-6-methoxy-1,4-benzoquinone
2OPMB	2-Octaprenyl-6-methoxy-1,4-benzoquinone
2OPMMB	2-Octaprenyl-3-methyl-6-methoxy-1,4-benzoquinone
2OPMP	2-Octaprenyl-6-methoxyphenol
2OPPP	2-Octaprenylphenol
2P14NQ	2-Phytyl-1,4-naphthoquinone
2PCDPMDE	2-Phospho-4-(cytidine 5'-diphospho)-2-C-methyl-D-erythritol
2PG	2-Phospho-D-glycerate
3DDAH7P	2-Dehydro-3-deoxy-D-arabino-heptonate 7-phosphate
3PADSELNT	3'-Phosphoadenylylselenate
3PG	3-Phospho-D-glycerate
3PSER	3-Phosphoserine
3PSME	5-O-(1-Carboxyvinyl)-3-phosphoshikimate
3SFPYR	3-Sulfinylpyruvate
3SLALA	3-Sulfino-L-alanine
3SPYR	3-Sulfopyruvate
4HB	4-Hydroxybenzoate
4HBZ	4-Hydroxybenzoate
4HLT	4-Hydroxy-L-threonine
4PPNCYS	(R)-4'-Phosphopantothenoyl-L-cysteine
4PPNTE	Pantetheine 4'-phosphate
4PPNTO	D-4'-Phosphopantothenate
5MTA	5'-Methylthioadenosine
5MTR	5-Methylthio-D-ribose
5MTR1P	5-Methylthio-5-deoxy-D-ribulose 1-phosphate
5MTRP	S5-Methyl-5-thio-D-ribose 1-phosphate
A6RP	5-Amino-6-ribitylamino-2,4 (1H, 3H)-pyrimidinedione
A6RP5P	5-Amino-6-(5'-phosphoribosylamino)uracil
A6RP5P2	5-Amino-6-(5'-phosphoribitylamino)uracil
AA	D-Alanyl-D-alanine
ABUT	(S)-2-Aceto-2-hydroxybutanoate
AC	Acetate
ACAC	Acetoacetate
ACACP	Acetyl-ACP
ACAL	Acetaldehyde
ACALxt	external Acetaldehyde
ACCOA	Acetyl-CoA
ACLAC	2-Acetolactate
ACMU6P	N-Acetylmuramic acid 6-phosphate
ACP	Acyl-carrier protein
ACSPG	N-Acylsphingosine
ACTN	Acetoin
ACTNxt	external Acetoin
ACxt	external Acetate
ACYABUT	gamma-Amino-gamma-cyanobutanoate
ACYCOA	Acyl-CoA
AD	Adenine
ADCHOR	4-amino-4-deoxychorismate
ADLIPO	6-S-Acetyldihydrolipoamide
ADN	Adenosine
ADP	ADP
ADPGLC	ADPglucose
ADSELNT	Adenylylselenate
ADSHCYS	Se-Adenosylselenohomocysteine
ADSMET	Se-Adenosylselenomethionine
AG	L-Arogenate
AGL3P	Acyl-sn-glycerol 3-phosphate
AGMAT	Agmatine
AHHMD	2-Amino-7,8-dihydro-4-hydroxy-6-(diphosphooxymethyl)pteridine
AHHMP	2-Amino-4-hydroxy-6-hydroxymethyl-7,8-dihydropteridine
AHM	4-Amino-5-hydroxymethyl-2-methylpyrimidine
AHMPP	4-Amino-5-hydroxymethyl-2-methylpyrimidine-pyrophosphate
AHTD	2-Amino-4-hydroxy-6-(erythro-1,2,3-trihydroxypropyl)-dihydropteridine triphosphate
AICAR	1-(5'-Phosphoribosyl)-5-amino-4-imidazolecarboxamide
AIR	Aminoimidazole ribotide
AKG	2-Oxoglutarate
AKP	a-Ketopantoate
ALA	L-Alanine; Alanine
ALAV	D-Aminolevulinate
AMP	AMP
AMPMPM	4-Amino-2-methyl-5-phosphomethylpyrimidine

Abbreviation	[1]Metabolite
AMPxt	external AMP
AN	Anthranilate
AONA	8-amino-7-oxononanoate
AOPOB	2-Amino-3-oxo-4-phosphonooxybutyrate
AOPP	3-Amino-2-oxopropyl phosphate
APRCN	alpha-Aminopropiononitrile
APS	Adenylylsulfate
ARG	L-Arginine
ARGSUCC	N-(L-Arginino)succinate
ARIB	ADPribose; ADP-ribose
ASER	O-Acetyl-L-serine
ASN	L-Asparagine
ASP	L-Aspartate
ASPSA	L-Aspartate 4-semialdehyde
ASUC	N6-(1,2-Dicarboxyethyl)-AMP
ATHR	L-Allothreonine
ATP	ATP
B5AMP	Biotinyl-5'-AMP
bALA	beta-Alanine
BASP	4-Phospho-L-aspartate
bDG6P	beta-D-Glucose 6-phosphate
bDGLC	beta-D-Glucose
bGALbGLCM	beta-D-Galactosyl-1,4-beta-D-glucosylceramide or Lactosylceramide
Biomass	Biomass
BT	Biotin
BUTAL	Butanal
BZ	Benzoate
BZNIT	Benzonitrile
C120ACP	Dodecanoyl-[acyl-carrier protein]
C140ACP	Myristoyl-[acyl-carrier protein]
C141ACP	cis-tetradec-7-enoyl-[acyl-carrier protein] (n-C14:1)
C15815	Thiamine biosynthesis intermediate 6
C160ACP	Hexadecanoyl-[acyl-carrier protein]
C161ACP	cis-hexadec-9-enoyl-[acyl-carrier protein] (n-C16:1)
C180ACP	Octadecanoyl-ACP (n-C18:0ACP)
C181ACP	cis-octadec-11-enoyl-[acyl-carrier protein] (n-C18:1)
C190ACP	Nonadecanoyl-[acyl-carrier protein]
C2H4	Ethylene
CAASP	N-Carbamoyl-L-aspartate
CAIR	1-(5-Phospho-D-ribosyl)-5-amino-4-imidazolecarboxylate
CAP	Carbamoyl phosphate
CDP	CDP
CDPDG	CDP-diacylglycerol
CDPMDE	4-(Cytidine 5'-diphospho)-2-C-methyl-D-erythritol
CHCOA	6-carboxyhexanoyl-CoA; Pimeloyl-CoA
CHO	Choline
CHOR	Chorismate
CIT	Citrate
CITR	L-Citrulline
CL	Cardiolipin (biomass component)
CLAALD	trans-3-Chloroallyl aldehyde
CLCMBO	2,5-Dichloro-carboxymethylenebut-2-en-4-olide
CLMAC	2-Chloromaleylacetate
CLPOL	trans-3-Chloro-2-propene-1-o
CMBO	trans-4-Carboxymethylenebut-2-en-4-olide
CMP	CMP
CO2	CO2
CO2xt	external CO2
COA	CoA; coenzyme A
CPAD5P	1-(2-Carboxyphenylamino)-1-deoxy-D-ribulose 5-phosphate
CPP	Coproporphyrinogen III
CTB	Chitobiose
CTP	CTP
CYAALA	3-Cyano-L-alanine
CYS	L-Cysteine
CYSGLY	Cys-Gly
CYST	L-Cystine
CYSTEATE	L-Cysteate
CYTD	Cytidine
D23PIC	2,3-Dihydro dipicolinate
D26PIM	L,l-2,6-Diamino pimelate
D6PGC	6-Phospho-D-gluconate
D6PGL	D-Glucono-1,5-lactone 6-phosphate
D6RP5P	2,5-Diamino-6-hydroxy-4-(5'-phosphoribosylamino)-pyrimidine
D8RL	6,7-Dimethyl-8-(1-D-ribityl)lumazine
DA	Deoxyadenosine
DADP	dADP
DALA	D-alanine
DALAxt	external DALA
DAMP	dAMP
DANNA	7,8-Diaminononanoate
DAPMNTP	2,5-Diaminopyrimidine nucleoside triphosphate

Abbreviation	[1]Metabolite
DASP	D-Aspartate
DATP	dATP
DATPTHOPAOPM	2,5-Diamino-6-(5'-triphosphoryl-3',4'-trihydroxy-2'-oxopentyl)-amino-4-oxopyrimidine
DB4P	L-3,4-Dihydroxy-2-butanone 4-phosphate
DC	Deoxycytidine
DCDP	dCDP
DCLCMBO	2,5-Dichloro-carboxymethylenebut-2-en-4-olide
DCLOHE	2,5-Dichloro-4-oxohex-2-enedioate
DCMP	dCMP
DCTP	dCTP
DG	Deoxyguanosine
DGDP	dGDP
DGLU	D-Glutamate
DGLY	D-glycerate
DGLYCERATE	D-Glycerate
DGMP	dGMP
DGTP	dGTP
DHACT	Dihydroxyacetone
DHACTxt	external Dihydroxyacetone
DHBZ	3,4-Dihydroxybenzoate
DHDHBZ	DHDHBZ: (2S,3S)-2,3-Dihydro-2,3-dihydroxybenzoate
DHF	Dihydrofolate
DHMA	3,4-Dihydroxymandelaldehyde
DHMB	(R)-2,3-Dihydroxy-3-methylbutanoate
DHMP	(R)-2,3-dihydroxy-3-methylpentanoate
DHP	2-Amino-4-hydroxy-6-(D-erythro-1,2,3-trihydroxypropyl)-7,8-dihydropteridine
DHPEG	3,4-Dihydroxyphenylethyleneglycol
DHPT	Dihydropteroate
DHSK	3-Dehydroshikimate
DIMGP	D-erythro-1-(Imidazol-4-yl)glycerol 3-phosphate
DIN	Deoxyinosine
DKMPP	2,3-Diketo-5-methylthiopentyl-1-phosphate
DLIPO	Dihydrolipoamide
DMPP	Dimethylallyl diphosphate; Dimethylallyl PPI
DMQ	2-Demethylmenaquinone
DNA	DNA (biomass component)
DOROA	(S)-Dihydroorotate
DOT	3-Dehydroquinate
DPCOA	Dephospho-CoA
DSAM	Decarboxylated adenosylmethionine
DT	Thymidine
DTB	Dethiobiotin
DTDP	dTDP
DTMP	dTMP
DTTP	dTTP
DU	Deoxyuridine
DUDP	dUDP
DUMP	dUMP
DUTP	dUTP
DX5P	1-Deoxy-D-xylulose 5-phosphate
E3MM	D-erythro-3-Methylmalate
E4HGLU	L-erythro-4-Hydroxyglutamate
E4P	D-Erythrose 4-phosphate
EO	Ethylene oxide
ETH	Ethanol
ETHA	Ethanolamine
ETHxt	external Ethanol
F1P	D-Fructose 1-phosphate
F6P	beta-D-Fructose 6-phosphate
FAD	Flavin adenine dinucleotide; FAD
FADH2	FADH2
FALD	Formaldehyde
FAPNTP	Formamidopyrimidine nucleoside triphosphate
FDP	beta-D-Fructose 1,6-bisphosphate
Fe	Fe2+
FGAM	2-(Formamido)-N1-(5'-phosphoribosyl)acetamidine
FGAR	5'-Phosphoribosyl-N-formylglycinamide
FL	Folate
FMGT	S-Formylglutathione
FMN	FMN; Riboflavin-5-phosphate; Flavin mononucleotide
FMUCLAC	5-Fluoromuconolactone
FORGLU	N-Formyl-L-glutamate
FORT	Formate
FORTxt	external Formate
FPP	trans,trans-Farnesyl diphosphate; trans,trans-Farnesyl PPI
FRU	D-Fructose
FRUxt	external Fructose
FTHF	10-Formyltetrahydrofolate
FUM	Fumarate
FUMxt	external Fumarate
g14l	L-Gulono-1,4-lactone
G1P	D-Glucose 1-phosphate

Abbreviation	[1]Metabolite
G3PC	sn-glycero-3-Phosphocholine
G3PE	sn-glycero-3-Phosphoethanolamine
G6GG	D-Gal alpha 1->6D-Gal alpha 1->6D-Glucose
G6P	alpha-D-Glucose 6-phosphate
GA1P	D-Glucosamine 1-phosphate
GA6P	D-Glucosamine 6-phosphate
GAR	5'-Phosphoribosylglycinamide
GC	L-g-Glutamylcysteine
GDP	GDP
GGPP	Geranylgeranyl diphosphate; trans,trans,cis-Geranylgeranyl PPI
GL	Glycerol
GL3P	sn-Glycerol 3-phosphate
GLAC	D-Galactose
GLAL	Glycolaldehyde
GLC	alpha-D-Glucose
GLCN	Gluconate
GLCNDL	Glucono 1,5-lactone
GLCxt	external Glucose
GLN	L-Glutamine
GLU	L-Glutamate
GLUAPRCN	gamma-Glutamyl-beta-aminopropiononitrile
GLUC	D-Gluconate; D-Gluconic acid; D-gluco-Hexonic acid
GLUCM	Glucosylceramide
GLUCYALA	gamma-Glutamyl-beta-cyanoalanine
GLUGSAL	L-Glutamate 5-semialdehyde
GLUP	alpha-D-Glutamyl phosphate
GLX	Glyoxylate
GLxt	external Glycerol
GLY	Glycine
GLYCOAL	Glycolaldehyde
GLYCOGEN	Glycogen
GLYCOLATE	Glycolate
GMP	GMP
GN	Guanine
GPP	Geranyl diphosphate; Geranyl PPI
GSN	Guanosine
GTP	GTP
guln	L-Gulonate
H2CO3	Carbonic acid
H2O2	H2O2
H2S	Hydrogen sulfide
H2SO3	Sulfite
H3PPBZ	4-Hydroxy-3-polyprenylbenzoate
HAL	Halide
HC2H	Acetylene
HCT	(R)-2-Hydroxybutane-1,2,4-tricarboxylate; Homocitrate; Homocitric acid
HCYS	L-Homocysteine
HEPPP	all-trans-Heptaprenyl diphosphate
HEXT	external hydrogen
HFA	Hydrofluoric acid
HIS	L-Histidine
HISAL	L-Histidinal
HISOL	L-Histidinol
HISOLP	L-Histidinol phosphate
HMB	Hydroxymethylbilane
HMB4PP	1-Hydroxy-2-methyl-2-butenyl 4-diphosphate
HMGT	S-(Hydroxymethyl)glutathione
HMNAPTH	1-Hydroxymethylnaphthalene
HMOBA	3-Methyl-2-oxobutanoic acid
HMOPENT	(R)-3-Hydroxy-3-methyl-2-oxopentanoate
HO3S2	Thiosulfate
HOPANOIDS	HOPANOIDS
HOPANOL	HOPANOL
HOPENE	HOPENE
HPHPYR	para-Hydroxy phenyl pyruvate
HPRO	trans-4-Hydroxy-L-proline
HPYR	Hydroxypyruvate
HSER	L-Homoserine
HSO3	Bisulfite
HTPP	2-(alpha-Hydroxyethyl)thiamine diphosphate
HYDROXYAKG	D-4-Hydroxy-2-oxoglutarate
I3P	1D-myo-Inositol 3-phosphate
I4P	myo-Inositol 4-phosphate
IAC	Indole-3-acetate
ICHOR	Isochorismate
ICIT	Isocitrate
IDACAL	Indole-3-acetaldehyde
IDACTN	3-Indoleacetonitrile
IDPYR	Indolepyruvate
IGP	Indoleglycerol phosphate
IHKP	myo-Inositol hexakisphosphate
ILE	L-Isoleucine

Abbreviation	[1]Metabolite
IMACP	3-(Imidazol-4-yl)-2-oxopropyl phosphate
IMGLY	Iminoglycine
IMP	IMP
INDOLE	Indole
INS	Inosine
INSTOL	Inositol
IP	inositol phosphate
IPKP	Inositol 1,2,3,5,6-pentakisphosphate
IPP	Isopentenyl diphosphate
IPPMAL	2-Isopropylmalate
IPPMALE	2-Isopropylmaleate
IPPP	Isopentenyl diphosphate
ISUCC	a-Iminosuccinate
ITCN	Itaconate
ITCNCOA	Itaconyl-CoA
ITP	ITP
K	potassium
KMB	a- keto-g-methiobutyrate
Kxt	external Potassium
LAC	(R)-Lactate, D-Lactate
LACxt	external Lactate
LCTS	Lactose
LEU	L-Leucine
LEVAN	LEVAN
LEVANxt	external LEVAN
LIPO	Lipoamide
LLCT	L-Cystathionine
LYS	L-Lysine
MAL	(S)-Malate
MALACP	Malonyl-[acyl-carrier protein]
MALCOA	Malonyl-CoA
MALxt	external Malate
MAN1P	alpha-D-Mannose 1-phosphate
MAN6P	D-Mannose 6-phosphate
MDAP	meso-2,6-Diaminoheptanedioate
MDE4P	2-C-Methyl-D-erythritol 4-phosphate
MDECPP	2-C-Methyl-D-erythritol 2,4-cyclodiphosphate
MELI	Melibiose
MET	L-Methionine
METHANOL	Methanol
METHF	5,10-Methenyltetrahydrofolate
METTHF	5,10-Methylenetetrahydrofolate
MOBA	3-Methyl-2-oxobutanoic acid
MOPENA	(S)-3-Methyl-2-oxopentanoic acid
MPYR	Mercaptopyruvate
MQ	Menaquinone
MTE	Methanethiol
MTGLU	5-Methyltetrahydropteroyltri-L-glutamate
MTGYX	Methylglyoxal
MTHF	5-Methyltetrahydrofolate
MTSEL	Methaneselenol
N2	Nitorgen
NA	Sodium
NAAD	Deamido-NAD+
NAARON	N-a-Acetyl ornithine
NAC	Nicotinate
NACxt	external Nicotinate
NAD	NAD+; Nicotinamide adenine dinucleotide
NADH	NADH
NADP	NADP+; Nicotinamide adenine dinucleotide phosphate
NADPH	NADPH
NADxt	external NAD+
NAG1P	N-Acetylglucosamine 1-phosphate
NAGA	N-Acetyl-D-glucosamine
NAGLU	N-Acetyl-L-glutamate
NAGLUSAL	N-Acetyl glutamate semialdehyde
NAGLUYP	N-Acetyl glutamyl -phosphate
NAGP	N-Acetylglucosamine 6-phosphate
NAM	Nicotinamide
NAMN	Nicotinate D-ribonucleotide
NAMNS	Nicotinate D-ribonucleoside
NAPTHAH	1-Naphthaldehyde
NAPTHM	(2-Naphthyl)methanol
NAxt	external Sodium
NCAIR	5'-Phosphoribosyl-5-carboxyaminoimidazole
NCPTRC	N-Carbamoylputrescine
NDP	Nucleoside diphosphate
NH3	NH3
NH3xt	external Ammonia
NMN	Nicotinamide D-ribonucleotide
NMNxt	external nicotinamide D-ribonulceotide
NPH	4-Nitrophenol

Abbreviation	[1]Metabolite
NPHP	4-Nitrophenyl phosphat
NPRAN	N-(5-Phospho-D-ribosyl)anthranilate
NS26DP	N-Succinyl-l,l-2,6-diaminopimelate
NS2A6O	N-Succinyl-2-amino-6-ketopimelate
NTP	Nucleoside triphosphate
O2	Oxygen
O2xt	external Oxygen
O4HBZ	3-Octaprenyl-4-hydroxybenzoate
OA	Oxaloacetate
OAHSER	O-Acetyl-L-homoserine
OAP	3-Oxoadipate
OBUT	2-Oxobutanoate
ODHFAC	2-Oxo-2,3-dihydrofuran-5-acetate
OFD	Oxidized ferredoxin
OFRDX	Oxidized ferredoxin
OGT	Oxidized glutathione
OHB	3-Hydroxy-4-phospho-hydroxy-alpha-ketobutyrate
OICAP	3-Carboxy-4-methyl-2-oxopentanoate
OMP	Orotidine 5'-phosphate
OPP	all-trans-Octaprenyl diphosphate
OPPPI	all-trans-Octaprenyl PPI
ORN	L-Ornithine
OROA	Orotate
OTHIO	Oxidized thioredoxin
P5P	Pyridoxine 5'-phosphate; Pyridoxine phosphate
PA	Phosphatidate; 1,2-Diacyl-sn-glycerol 3-phosphate
PABA	4-Aminobenzoate
PAMN	Protoanemonin
PANT	(R)-pantoate
PAP	Adenosine 3',5'-bisphosphate
PAPS	3'-Phosphoadenylyl sulfate
PBG	Porphobilinogen
PC	Phosphatidylcholine
PC2	Percorrin 2
PDLA	Pyridoxamine
PDLA5P	Pyridoxamine-5-phosphate
PE	Phosphatidylethanolamine
PEP	Phosphoenolpyruvate
PEPTIDO	Peptidoglycan (biomass component)
PG	Phosphatidylglycerol
PGP	Phosphatidylglycerophosphate
PHE	L-Phenylalanine
PHEN	Prephenate
PHOSPHOLIPID	Phospholipids (biomass component)
PHP	3-Phosphonooxypyruvate
PHPYR	Phenylpyruvate
PHSER	O-Phospho-L-homoserine
PHT	O-Phospho-4-hydroxy-L-threonine
PI	Orthophosphate
PINSTOL	Phosphatidylinositol
PIP26DX	Delta-piperidine-2,6-dicarboxylate
PIxt	external Orthophosphate
PL	Pyridoxal
PL5P	Pyridoxal 5'-phosphate
PNCYS	N-((R)-Pantothenoyl)-L-cysteine
PNDME	Phosphatidyl-N-dimethylethanolamine
PNME	phosphatidyl-N-methylethanolamine
PNTO	(R)-Pantothenate; Pantothenate
PNTOxt	external Pantothenate
PP3M5H6M14BQ	2-Polyprenyl-3-methyl-5-hydroxy-6-methoxy-1,4-benzoquinone
PP3M6M14BQ	2-Polyprenyl-3-methyl-6-methoxy-1,4-benzoquinone
PP6HPH	2-Polyprenyl-6-hydroxyphenol
PP6M14BQ	2-Polyprenyl-6-methoxy-1,4-benzoquinone
PP6MPH	2-Polyprenyl-6-methoxyphenol
PPACOA	Propanoyl-CoA; Propionyl-CoA
PPEPTIDO	Peptidoglycan precursor
ppGpp	Guanosine 3',5'-bis(diphosphate)
PPHG	Protoporphyrinogen IX
PPI	Pyrophosphate; Diphosphate
PPIX	Protoporphyrin IX; Protoporphyrin
pppGpp	Guanosine 3'-diphosphate 5'-triphosphate
PPPH	2-Polyprenylphenol
PPPI	Inorganic triphosphate
PPPP	all-trans-Polyprenyl PPI
PQ	Phylloquinone
PRAM	5-Phosphoribosylamine
PRBAMP	N1-(5-Phospho-D-ribosyl)-AMP
PRBATP	N1-(5-Phospho-D-ribosyl)-ATP
PRFICA	1-(5'-Phosphoribosyl)-5-formamido-4-imidazolecarboxamide
PRFP	5-(5-Phospho-D-ribosylaminoformimino)-1-(5-phosphoribosyl)-imidazole-4-carboxamide
PRLP	N-(5'-Phospho-D-1'-ribulosylformimino)-5-amino-1-(5''-phospho-D-ribosyl)-4-imidazolecarboxamide

Abbreviation	[1]Metabolite
PRO	L-Proline
PROTEIN	protein
PRPP	5-Phospho-alpha-D-ribose 1-diphosphate; 5-Phosphoribosyl diphosphate; PRPP
PS	Phosphatidylserine
PTH	Protoheme
PTRC	Putrescine
PTT	Pantetheine
PYCA	1-Pyrroline-5-carboxylate
PYR	Pyruvate
PYRDX	Pyridoxine
PYRRHCAR	L-1-Pyrroline-3-hydroxy-5-carboxylate
PYTHP	6-Pyruvoyltetrahydropterin
Q	Ubiquinone
QA	Quinolinate; Pyridine-2,3-dicarboxylate
QH2	Ubiquinol
R1P	D-Ribose 1-phosphate
R2MM	(R)-2-Methylmalate
R5P	D-Ribose 5-phosphate
RAF	Raffinose
RCN	Nitrile
RCO2	Carboxylate
RFD	Reduced ferredoxin
RFEDX	Reduced ferredoxin
RGT	Glutathione
RIBFLV	Riboflavin
RL5P	D-Ribulose 5-phosphate
RNA	RNA
RNAM	N-Ribosylnicotinamide
RSCYS	R-S-Cysteinylglycine
RSCYSGLY	R-S-Cysteinylglycine
RSGSH	R-S-Glutathione
RTHIO	Thioredoxin
RX	Organic halide
S	Sulfur
S6P	Sorbitol 6-phosphate
S7P	Sedoheptulose 7-phosphate
SAH	S-Adenosyl-L-homocysteine
SAICAR	1-(5'-Phosphoribosyl)-5-amino-4-(N-succinocarboxamide)-imidazole
SAM	S-Adenosyl-L-methionine
SAMOB	S-adenosyl-4-methylthio-2-oxobutanoate
SB1P	Sorbose 1-phosphate
SCYS	Selenocysteine
SELD	Selenide
SELNT	Selenate
SER	L-Serine
SHCL	Sirohydrochlorin
SHCYS	Selenohomocysteine
SHEME	Siroheme
SLF	Sulfate
SLFxt	external Sulfate
SLGT	(R)-S-Lactoylglutathione
SLLCT	Selenocystathionine
SMALL_MOLECULES	SMALL_MOLECULES
SME	Shikimate
SME5P	Shikimate-5-phosphate
SMET	Selenomethionine
SO2	sulfur dioxide
SOB	Sorbose
SOBxt	external sorbose
SOT	D-Sorbitol
SOTxt	external Sorbitol
SPMD	Spermidine
SQL	Squalene
ST	Stachyose
SUC	Sucrose
SUC6P	Sucrose 6-phosphate
SUCC	Succinate
SUCCARG	N2-Succinyl-L-arginine
SUCCGLU	N-Succinyl-L-glutamate
SUCCGLU5SAL	N-Succinyl-L-glutamate 5-semialdehyde
SUCCOA	Succinyl-CoA
SUCCORN	N2-Succinyl-L-ornithine
SUCCSAL	Succinate semialdehyde
SUCCxt	external Succcinate
SUCxt	external Sucrose
SULLAC	4-Sulfolactone
T3P1	Glyceraldehyde 3-phosphate
T3P2	Dihydroxyacetone phosphate or glycerone phosphate
TAG	Triacylglycerol
TCYS	Thiocysteine
TGLU	Tetrahydropteroyltri-L-glutamate
THBH	Tetrahydroxybacteriohopanetetrol

Abbreviation	[1]Metabolite
THBHET	Tetrahydroxybacteriohopane-ether
THBHGA	Tetrahydroxybacteriohopane-glucosamine
THF	Tetrahydrofolate
THMP	Thiamin monophosphate
THMPP	Thiamin diphosphate
THPP	Thiamine diphosphate
THR	L-Threonine
THZP	4-Methyl-5-(beta-hydroxyethyl)thiazole phosphate
TPP	Thiamine-pyrophosphate
TRP	L-Tryptophan
TYR	L-Tyrosine
UAAGMDA	Undecaprenyl-diphospho-N-acetylmuramoyl-(N-acetylglucosamine)-L-alanyl-D-glutamyl-meso-2,6-diaminopimeloyl-D-alanyl-D-alanine
UAGMDA	Undecaprenyl-diphospho-N-acetylmuramoyl-L-alanyl-D-glutamyl-meso-2,6-diaminopimeloyl-D-alanyl-D-alanine
UDCPDP	Undecaprenyl diphosphate
UDCPP	Undecaprenyl diphosphate; di-trans,poly-cis-Undecaprenyl PPI ; all-trans,poly-cis-Undecaprenyl PPI
UDP	UDP
UDPG	UDP-glucose; UDPglucose; UDP-D-glucose; Uridine diphosphate glucose; UDP-alpha-D-glucose
UDPGLC	UDPglucuronate
UDPIDU	UDP-L-iduronate
UDPNAG	UDP-N-acetyl-D-glucosamine
UDPNAGEP	UDP-N-acetyl-3-(1-carboxyvinyl)-D-glucosamine
UDPNAM	UDP-N-acetylmuramate
UDPNAMA	UDP-N-acetylmuramoyl-L-alanine
UDPNAMAG	UDP-N-acetylmuramoyl-L-alanyl-D-glutamate
UMP	UMP
UNAGD	UDP-N-acetylmuramoyl-L-alanyl-D-gamma-glutamyl-meso-2,6-diaminopimelate
UNAGDA	UDP-N-acetylmuramoyl-L-alanyl-D-glutamyl-meso-2,6-diaminopimeloyl-D-alanyl-D-alanine
UPRG	Uroporphyrinogen III
UREA	Urea
URI	Uridine
UTP	UTP
VAL	L-Valine
VAN	Vanillate
X5P	D-Xylulose-5-phosphate
XAN	Xanthine
XMP	Xanthosine 5'-phosphate
XTP	XTP
XTSN	Xanthosine
**These metabolites are inserted to metabolize pentose sugars.**	
XYL	D-Xylose
XYLU	D-Xylulose
ARA	L-Arabinose
RIB	L-Ribulose
LRL5P	L-Ribulose 5-phosphate

1. The source of each metabolite is same as the reaction that they belong.
