# Supplementary material for: The genome-scale metabolic network analysis of Zymomonas mobilis ZM4 explains physiological features and suggests ethanol and succinic acid production strategies
Source: Microb Cell Fact. 2010 Nov 24;9:94. doi: 10.1186/1475-2859-9-94 (PMC3004842; doi:10.1186/1475-2859-9-94)
Supplement: Additional file 3 — ORF (Open Reading Frame) coverage of reconstructed metabolic models [file 1475-2859-9-94-S3.PDF]

Additional file 3. ORF (Open Reading Frame) coverage of reconstructed metabolic models

Organism	[1]Model	Coverage (%)	Ref
Escherichia coli	**iAF1260**	**28.0**	**Feist *et al* (2007)**
Saccharomyces cerevisiae	**iND750**	**12.0**	**Duarte *et al* (2004)**
	**iMM904**	**14.4**	**Mo *et al* (2009)**
Aspergillus nidulans	**iHD666**	**7.0**	**David *et al* (2008)**
Aspergillus niger		**6.1**	**David *et al* (2003)**
Arabidopsis thaliana	**AraGEM**	**5.2**	**de Oliveira Dal'Molin *et al* (2010)**
Mus musculus		**3.0**	**Selvarasu *et al* (2010)**
Homo sapiens	**Recon 1**	**6.3**	**Duarte *et al* (2007)**
Acinetobacter baumannii	**AbyMBEL891**	**17.3**	**Kim *et al* (2010)**
Helicobacter pylori	**iIT341**	**26.7**	**Schilling *et al* (2008)**

1. There are metabolic models which do not have its own name.

References

Feist *et al*., A genome-scale metabolic reconstruction for *Escherichia coli* K-12 MG1655 that accounts for 1260 ORFs and thermodynamic information. *Mol Syst Biol*. 2007; 3:121
Duarte *et al*., Reconstruction and validation of *Saccharomyces cerevisiae* iND750, a fully compartmentalized genome-scale metabolic model. *Genome Res*. 2004; 14:1298-1309.
Mo *et al*., Connecting extracellular metabolomic measurements to intracellular flux states in *yeast*. *BMC Syst Biol*. 2009; 3:37.
David *et al*., Analysis of *Aspergillus nidulans* metabolism at the genome-scale. *BMC Genomics*. 2008; 9:163.
David *et al*., Reconstruction of the central carbon metabolism of *Aspergillus niger*. *Eur J Biochem*. 2003; 270:4243-4253.
de Oliveira Dal'Molin *et al*., AraGEM, a genome-scale reconstruction of the primary metabolic network in *Arabidopsis*. *Plant Physiol*. 2010; 152:579-589.
Selvarasu *et al*., Genome-scale modeling and *in silico* analysis of mouse cell metabolic network. *Mol Biosyst*. 2010; 6:152-161.
Duarte *et al*., Reconstruction and validation of *Saccharomyces cerevisiae* iND750, a fully compartmentalized genome-scale metabolic model. *Genome Res*. 2004; 14:1298-1309.
Kim *et al*., Genome-scale metabolic network analysis and drug targeting of multi-drug resistant pathogen *Acinetobacter baumannii* AYE. *Mol. BioSyst*. 2010; 6, 339-348
Schilling *et al*., Genome-Scale Metabolic Model of *Helicobacter pylori* 26695. *J Bac*. August 2002; 184(16): 4582-4593
