# Supplementary material for: The genome-scale metabolic network analysis of Zymomonas mobilis ZM4 explains physiological features and suggests ethanol and succinic acid production strategies
Source: Microb Cell Fact. 2010 Nov 24;9:94. doi: 10.1186/1475-2859-9-94 (PMC3004842; doi:10.1186/1475-2859-9-94)
Supplement: Additional file 4 — Biomass composition of Zymomonas mobilis ZM4 [file 1475-2859-9-94-S4.PDF]

Additional file 4. Biomass composition of *Zymomonas mobilis* ZM4

Macromolecular composition

Component	Composition (g/gDCW)	Reference
**Protoplast**	0.912	
Protein	0.605	J Swings and J De Ley (1977), A. A. De Graaf *et al* (1999)
DNA	0.027	J Swings and J De Ley (1977), A. A. De Graaf *et al* (1999)
RNA	0.195	J Swings and J De Ley (1977), A. A. De Graaf *et al* (1999)
Lipids	0.085	J Swings and J De Ley (1977), A. A. De Graaf *et al* (1999)
Phospholipids	0.053	Robert A. Moreau *et al* (1997)
Hopanoids	0.028	Robert A. Moreau *et al* (1997)
Non-polar (TAGs)	0.004	Robert A. Moreau *et al* (1997)
Small molecules	0.038	J Swings and J De Ley (1977), A. A. De Graaf *et al* (1999)
**Cell wall**	0.050	J Swings and J De Ley (1977), A. A. De Graaf *et al* (1999)
Peptidoglycan	0.025	J Swings and J De Ley (1977), A. A. De Graaf *et al* (1999)
Glycogen	0.025	J Swings and J De Ley (1977), A. A. De Graaf *et al* (1999)
SUM	1	

* Data are from J Swings and J De Ley (1977), A. A. De Graaf *et al* (1999), and Robert A. Moreau *et al* (1997)

Protein composition

Amino acid	% protein (g/g)	MW[a], g/mol	mmol/g protein
Alanine	15.243	71.094	2.144
Arginine	4.958	156.203	0.317
Asparagine	4.965	114.119	0.435
Aspartate	5.002	115.104	0.435
Cysteine	0.381	103.160	0.037
Glutamate	3.941	128.146	0.308
Glutamine	3.968	129.131	0.307
Glycine	10.86	57.067	1.903
Histidine	2.001	137.156	0.146
Isoleucine	7.611	113.175	0.672
Leucine	7.611	113.175	0.672
Lysine	5.724	128.189	0.447
Methionine	1.901	131.214	0.145
Phenylalanine	0.286	147.192	0.019
Proline	3.802	97.132	0.391
Serine	3.338	87.093	0.383
Threonine	4.196	101.120	0.415
Tryptophan	1.734	186.228	0.093
Tyrosine	1.994	163.191	0.122
Valine	10.482	99.148	1.057
Energy requirement for polymerisation (ATP):			44.92

* Water is substracted from MW to account for water excretion during peptide bond formation.

* Data on protein composition of *Z. mobilis* are from A. A. De Graaf *et al* (1999)

DNA composition

Nucleotide	mol/mol DNA	MW[a], g/mol	mmol/g DNA
dAMP	0.268	313.200	0.869
dCMP	0.232	289.200	0.750
dTMP	0.268	304.200	0.869
dGMP	0.232	329.200	0.750
Energy requirement for polymerisation (ATP):			4.40

* The molecular weight is the weight of the nucleotide monophosphate substracted one water molecule, which is lost during esterification.

* The ratio of the nucleic acids in the DNA was derived from the G/C-content according to the genome sequence (Seo *et al*, 2005): 46.33%

RNA composition

Nucleotide	mol/mol RNA			MW[a], g/mol	mol/mol RNA	mmol/g RNA
	mRNA 5%	rRNA 81%	tRNA 14%			
AMP	0.268	0.215	0.199	329.200	0.215	0.667
GMP	0.232	0.301	0.318	345.200	0.300	0.929
CMP	0.232	0.221	0.292	305.200	0.231	0.718
UMP	0.268	0.264	0.191	306.200	0.254	0.786
Energy requirement for polymerisation (ATP):						1.24

* The molecular weight is the weight of the nucleotide monophosphate substracted one water molecule, which is lost during esterification.

* It was assumed that RNA is consisted of 5% mRNA, 81% rRNA, and 14% tRNA which was taken from *E.coli* (5%, 80%, 15%) and corrleated with tRNA and rRNA gene from TIGR database which has 2.42% and 0.42% respectively.

* The nucleotide composition of mRNA was taken as for genomic DNA. The nucleotide composition of rRNA and tRNA was calculated from the sequences of rRNA and tRNA gene in TIGR database.

Phospholipids composition

Component	g/g phospholipids	mmol/g phospholipids
Cardiolipin(CL)	0.169	0.118
Phosphatidylethanolamine(PE)	0.478	0.655
Phosphatidylglycerol(PG)	0.041	0.054
Phosphatidylinositol(PINSTOL)	0.120	0.141
phosphatidylcholine(PC)	0.193	0.250

* The composition of phospholipids was taken from Robert A. Moreau *et al* (1995), ATCC 29191 strain.

Molecular weights of phospholipids components

Constituent	MW, g/mol	
	number of fatty acids residues	total
Cardiolipin	4	1427.732
Phosphatidylethanolamine	2	728.902
Phosphatidylglycerol	2	759.913
Phosphatidylinositol	2	847.974
phosphatidylcholine	2	771.990

Fatty acids composition in phospholipids

Fatty acid	MW[a], g/mol	mmol/g total fatty acids	mol/mol total fatty acids
Lauric acid (C12)	200.318	0.000	0.000
Myristic acid (C14)	228.371	0.365	0.100
Myristoleic acid (C14:1)	226.355	0.000	0.000
Palmitic acid (C16)	256.424	0.365	0.100
Palmitoleic acid (C16:1)	254.408	0.073	0.020
Stearic acid (C18)	284.477	0.000	0.000
Vaccenic acid (C18:1)	282.461	2.848	0.780
Nonadecanoic acid (C19:0)	296.490	0.000	0.000
Average molecular weight:	274	SUM:	1.00

* Molecular weight without a proton

* The composition of fatty acids tails in phospholipids was taken from V. C. Carey and L. O. Ingram (1983), ATCC 10988 strain.

Hopanoids composition

Component	g/g hopanoids	MW[a], g/mol	mmol/g hopanoids
Tetrahydroxybacteriohopanetetrol (THBH)	0.060	546.875	0.110
Tetrahydroxybacteriohopane-glucosamine (THBH-GA)	0.490	708.546	0.692
Tetrahydroxybacteriohopane-ether (THBH-ET)	0.410	708.546	0.579
Diplopterol	0.030	428.733	0.070
Hopene	0.010	410.718	0.024

* The composition of hopanoids was taken from M. A. Hermans *et al* (1991) ATCC 29191.

Triacylglycerols (TAGs) composition

TAG components	mol/mol TAG	mmol/g TAG
Glycerol-3-phosphate	1.000	1.235
Lauric acid (C12)	0.330	0.408
Myristic acid (C14)	0.360	0.445
Myristoleic acid (C14:1)	0.180	0.222
Palmitic acid (C16)	0.450	0.556
Palmitoleic acid (C16:1)	0.480	0.593
Stearic acid (C18)	0.090	0.111
Vaccenic acid acid (C18:1)	0.900	1.112
Nonadecanoic acid (C19:0)	0.210	0.259
Average molecular weight:	809.6	

* Triacylglycerols (TAGs) are composed of glycerol core and three fatty acids residues.

* The composition TAGs were taken from V. C. Carey and L. O. Ingram (1983), ATCC 10988 strain.

Fatty acids composition in TAG

Fatty acid	MW[a], g/mol	mmol/g total fatty acids	mol/mol total fatty acids
Lauric acid (C12)	200.318	0.429	0.110
Myristic acid (C14)	228.371	0.468	0.120
Myristoleic acid (C14:1)	226.355	0.234	0.060
Palmitic acid (C16)	256.424	0.585	0.150
Palmitoleic acid (C16:1)	254.408	0.624	0.160
Stearic acid (C18)	284.477	0.117	0.030
Vaccenic acid (C18:1)	282.461	1.171	0.300
Nonadecanoic acid (C19:0)	296.490	0.273	0.070
Average molecular weight:	256	SUM:	1.00

* The composition of fatty acids tails in TAGs was taken from V. C. Carey and L. O. Ingram (1983), ATCC 10988 strain.

Small molecules composition

Molecule	g/g pool of small molecules	mmol/g pool of small molecules
NAD	0.111	0.167
NADP	0.111	0.149
COA	0.111	0.145
ACP[a]	0.111	0.010
PTRC	0.111	1.260
SPMD	0.111	0.765
THF	0.111	0.249
FMN	0.111	0.243
FAD	0.111	0.141

[a] molecular mass of recombinant *E. coli* acyl carrier protein (Sigma-Aldrich)

* For simplification, it was assumed that the selected small molecules are equally represented (w/w) in the pool.

References

J Swings and J De Ley, The Biology of *Zymomonas. Bacteriol Rev*. March 1977; 41(1): 1–46.

A. A. De Graaf *et al*, Metabolic state of *Zymomonas mobilis* in glucose-, fructose-, and xylose-fed continuous cultures as analysed by ^{13}C- and ^{31}P-NMR spectroscopy. *Arch Microbiol*. May 1999; 171(6): 371-385.

Seo JS *et al*, The Genome Sequence of the Ethanologenic Bacterium *Zymomonas mobilis* ZM4. *Nature Biotech*. January 2005; 23(1): 63-68.

Robert A. Moreau *et al*, Analysis of Intact Hopanoids and Other Lipids from the Bacterium *Zymomonas Mobilis* by High-Performance Liquid Chromatography. *Anal Biochem*. January 1995; 224(1): 293-301.

Robert A. Moreau *et al*, The effect of ethanol and oxygen on the growth of *Zymomonas mobilis* and the levels of hopanoids and other membrane lipids. *Curr Microbiol*. August 1997; 35(2): 124-128.

V. C. Carey and L. O. Ingram, Lipid composition of *Zymomonas mobilis*: effects of ethanol and glucose. *J Bacteriol*. June 1983; 154(3): 1291-1300.

M. A. Hermans *et al*, Content and composition of hopanoids in *Zymomonas mobilis* under various growth conditions. *J Bacteriol*. September 1991; 173(17): 5592-5595.

E. A. Dawes and P. J . Large, Effect of starvation on the viability and cellular constituents of *Zymomonas anaerobia* and *Zymomonas mobilis. J Gen Microbiol*. January 1970; 60: 31-42.

A. Lazdunski and J. P. Belaich, Uncoupling in bacterial growth: ATP pool variation in *Zymomonas mobilis* cells in relation to different uncoupling conditions of growth. *J Gen Microbiol*. April 1972; 70:187-197.
