# Supplementary material for: The genome-scale metabolic network analysis of Zymomonas mobilis ZM4 explains physiological features and suggests ethanol and succinic acid production strategies
Source: Microb Cell Fact. 2010 Nov 24;9:94. doi: 10.1186/1475-2859-9-94 (PMC3004842; doi:10.1186/1475-2859-9-94)
Supplement: Additional file 5 — Trade-off curves of (A) engineered strain of Zymomonas mobilis for the utilization of xylose and arabinose and (B) single gene knockout mutants based on the engineered strain for glucose, xylose, and arabinose as a carbon source. [file 1475-2859-9-94-S5.PDF]

Additional file 5. Trade-off curves of (A) engineered strain of *Zymomonas mobilis* for the utilization of xylose and arabinose and (B) single gene knock out mutants based on the engineered strain for glucose, xylose, and arabinose as a carbon source. Uptake rate of each carbon source is fixed to 10 mmol/gDCW/h, and reaction for oxygen uptaking was deleted to describe anaerobic condition. NGAME (i.e. non-growth associated maintenance energy) value was eliminated

Additional figure 5 (A). Trade-off curves of engineered strain of *Z. mobilis* for glucose, xylose, and arabinose as a carbon source.

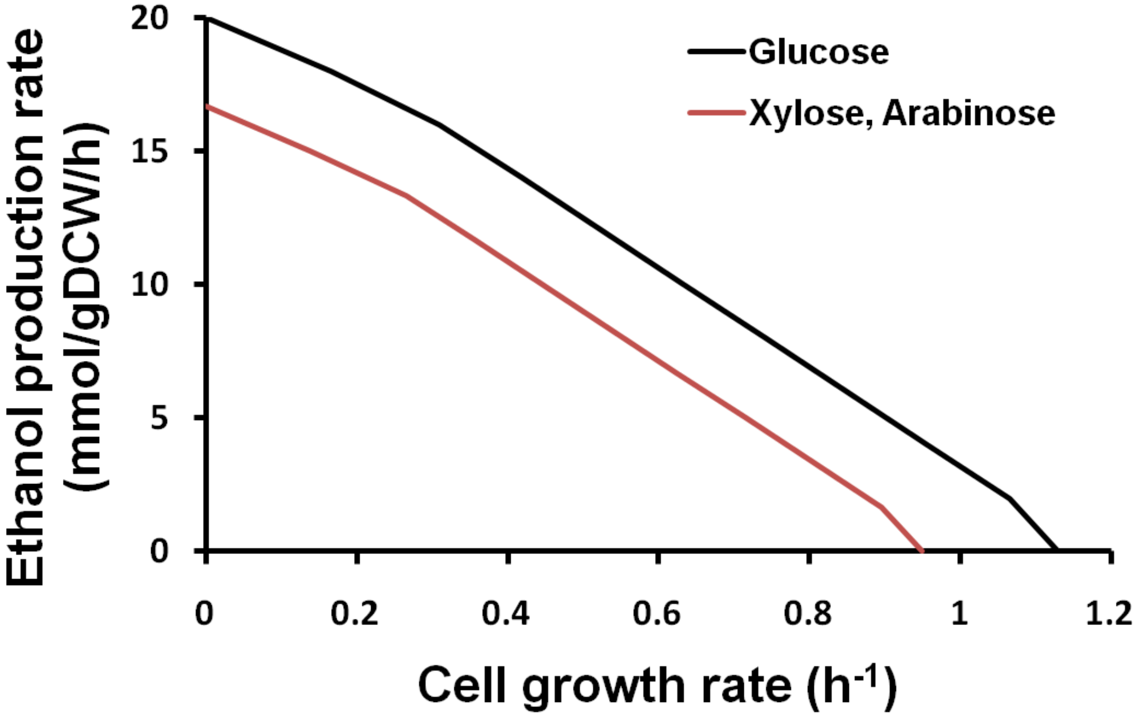

Additional figure 5 (B). Trade-off curves of single gene knockout mutants based on the engineered strain of *Z. mobilis* for glucose, xylose, and arabinose as a carbon source. The black and red lines indicate the trade-off curves of the engineered strain of *Z. mobilis* and the mutants by the deletion of each reaction in central metabolism listed below for each carbon source, respectively.

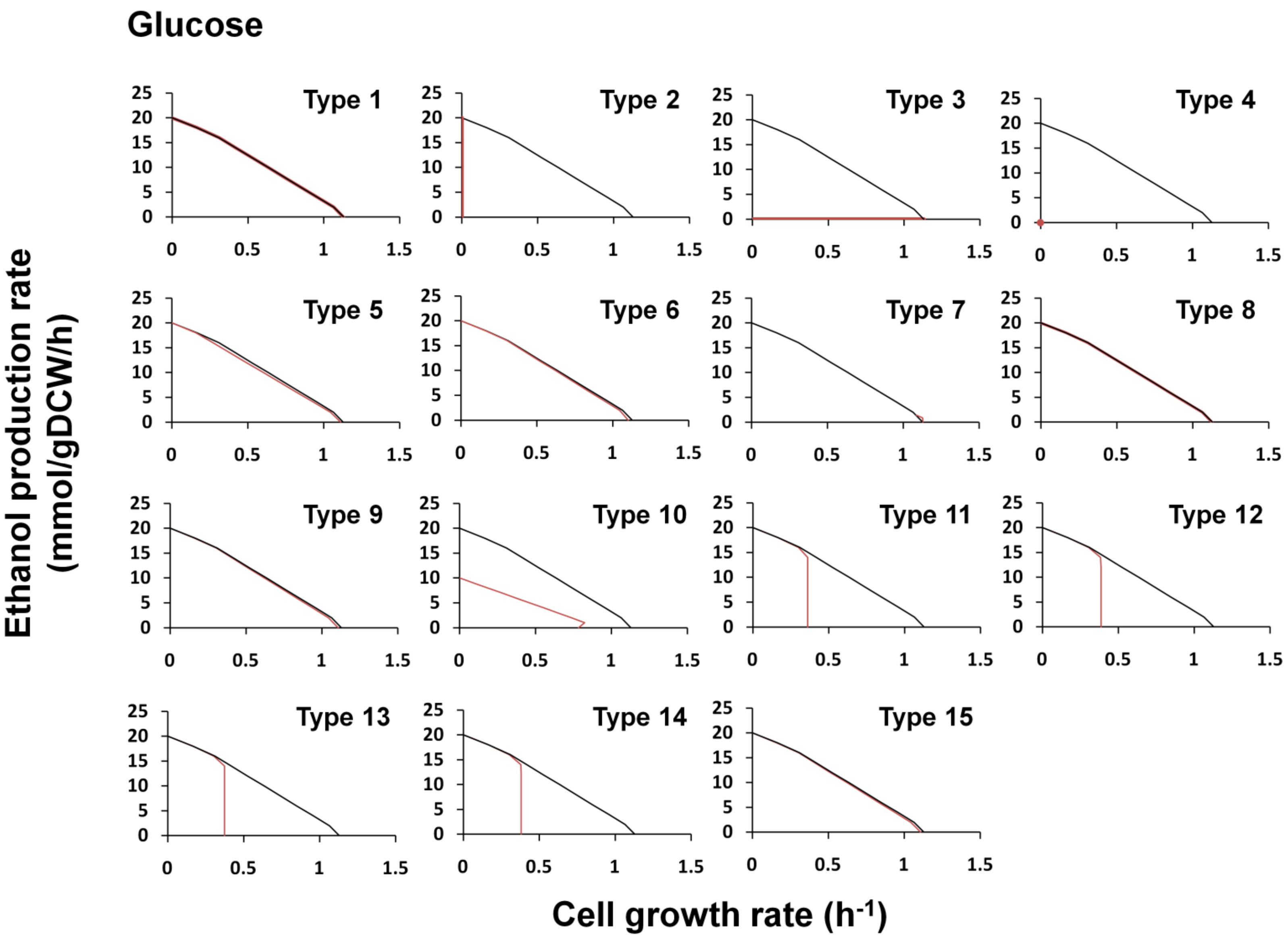

Type 1: R001, R002, R003, R004, R005, R006, R007, R021, R025, R027, R029, R030, R041, R042, R043, R044, R049, R050, R051, R053, R056, R057, R058, R060, R061, R062, R047, R048

Type 2: R008, R009, R010, R011, R012, R020, R022, R024, R028, R039, R040, R052, R055, R045, R046

Type 3: R019

Type 4: R033, R035

Type 5: R013

Type 6: R014, R015, R016, R017

Type 7: R018

Type 8: R023

Type 9: R026

Type 10: R031, R032

Type 11: R034

Type 12: R036

Type 13: R037, R059

Type 14: R038

Type 15: R054

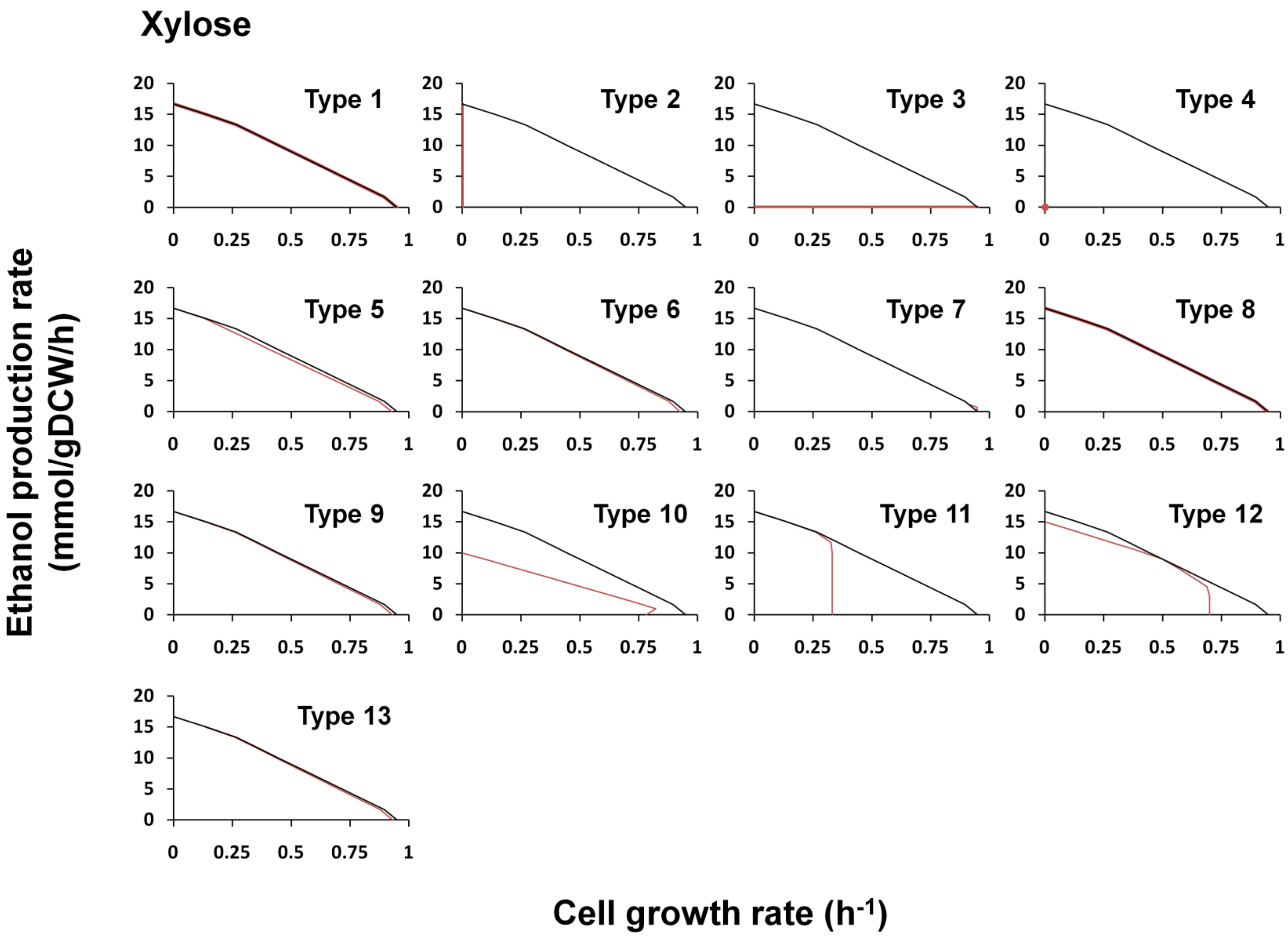
Xylose
Type 1
Type 2
Type 3
Type 4
Type 5
Type 6
Type 7
Type 8
Type 9
Type 10
Type 11
Type 12
Type 13
0
5
10
15
20
0
0.25
0.5
0.75
1
Ethanol production rate (mmol/gDCW/h)
Cell growth rate (h-1)

Type 1: R001, R002, R003, R004, R005, R006, R007, R021, R025, R027, R029, R030, R041, R042, R043, R044, R049, R050, R051, R053, R056, R060, R061, R062, R047, R048

Type 2: R008, R009, R010, R011, R012, R020, R022, R024, R028, R040, R052, R055, R045, R046

Type 3: R019

Type 4: R033, R035, R036, R037, R039, R057, R058, R059

Type 5: R013

Type 6: R014, R015, R016, R017

Type 7: R018

Type 8: R023

Type 9: R026

Type 10: R031, R032

Type 11: R034

Type 12: R038

Type 13: R054

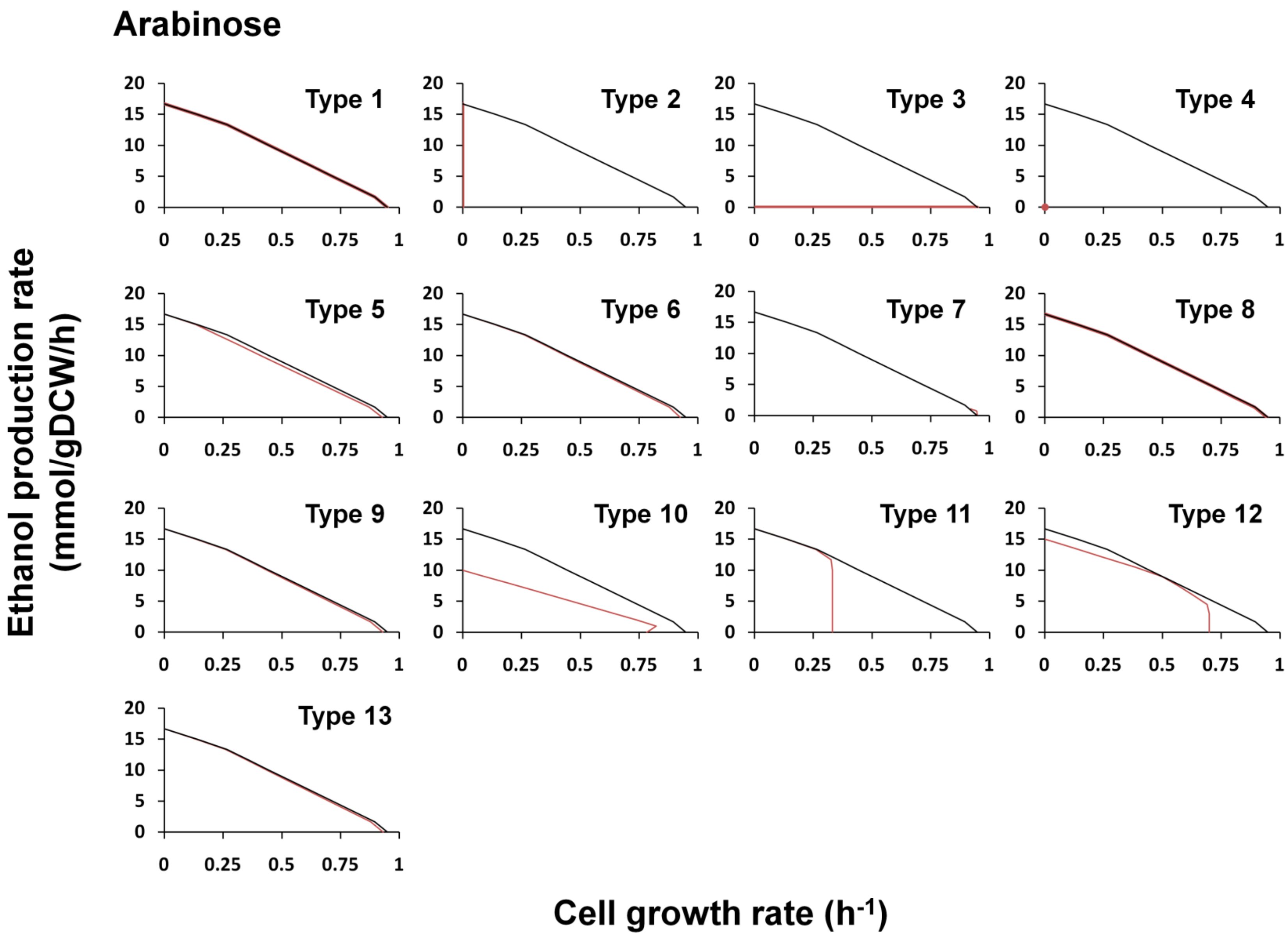
Arabinose
Type 1
Type 2
Type 3
Type 4
Type 5
Type 6
Type 7
Type 8
Type 9
Type 10
Type 11
Type 12
Type 13
20
15
10
5
0
0
0.25
0.5
0.75
1
Ethanol production rate (mmol/gDCW/h)
Cell growth rate (h^{-1})

Type 1: R001, R002, R003, R004, R005, R006, R007, R021, R025, R027, R029, R030, R041, R042, R043, R044, R049, R050, R051, R053, R056, R057, R058, R047, R048

Type 2: R008, R009, R010, R011, R012, R020, R022, R024, R028, R040, R052, R055, R045, R046

Type 3: R019

Type 4: R033, R035, R036, R037, R039, R059, R060, R061, R062

Type 5: R013

Type 6: R014, R015, R016, R017

Type 7: R018

Type 8: R023

Type 9: R026

Type 10: R031, R032

Type 11: R034

Type 12: R038

Type 13: R054

A list of reactions used in additional figure 5 (B) for the simulation of single gene knockout. Information of each reaction below is available in additional file 1 and 2.

R001: G6P <-> bDG6P (5.3.1.9)

R002: G6P <-> F6P (5.3.1.9)

R003: bDG6P <-> F6P (5.3.1.9)

R004: GLC + ATP -> G6P + ADP (2.7.1.2)

R005: bDGLC <-> GLC (5.1.3.3)

R006: FDP -> F6P + PI (3.1.3.11)

R007: FDP <-> T3P1 + T3P2 (4.1.2.13)

R008: T3P1 <-> T3P2 (5.3.1.1)

R009: T3P1 + PI + NAD <-> NADH + 13DPG (1.2.1.12)

R010: 13DPG + ADP <-> 3PG + ATP (2.7.2.3)

R011: 3PG <-> 2PG (5.4.2.1)

R012: 2PG <-> PEP (4.2.1.11)

R013: PEP + ADP -> PYR + ATP (2.7.1.40)

R014: PYR + THPP -> 2HETHPP + CO2 (1.2.4.1)

R015: 2HETHPP + LIPO -> ADLIPO + THPP (1.2.4.1)

R016: COA + ADLIPO -> DLIPO + ACCOA (2.3.1.12)

R017: DLIPO + NAD -> LIPO + NADH (1.8.1.4)

R018: PYR -> ACAL + CO2 (4.1.1.1)

R019: ACAL + NADH <-> ETH + NAD (1.1.1.1)

R020: G6P <-> G1P (5.4.2.2)

R021: bDGLC + ATP -> bDG6P + ADP (2.7.1.2)

R022: ACCOA + OA -> COA + CIT (2.3.3.1)

R023: CIT <-> AC + OA (4.1.3.6)

R024: CIT <-> ICIT (4.2.1.3)

R025: ICIT + NADP <-> CO2 + NADPH + AKG (1.1.1.42)

R026: FUM <-> MAL (4.2.1.2)

R027: FUM + FADH2 <-> SUCC + FAD (1.3.99.1)

R028: SUCCOA + ADP + PI <-> ATP + COA + SUCC (6.2.1.5)

R029: GLCNDL -> GLCN (3.1.1.17)

R030: ATP + GLCN -> ADP + D6PGC (2.7.1.12)

R031: 2KD6PG -> T3P1 + PYR (4.1.2.14)

R032: D6PGC -> 2KD6PG (4.2.1.12)

R033: D6PGL -> D6PGC (3.1.1.31)

R034: D6PGC + NADP -> NADPH + CO2 + RL5P (1.1.1.44)

R035: bDG6P + NADP <-> D6PGL + NADPH (1.1.1.49)

R036: X5P + E4P <-> F6P + T3P1 (2.2.1.1)

R037: R5P + X5P <-> T3P1 + S7P (2.2.1.1)

R038: RL5P <-> X5P (5.1.3.1)

R039: RL5P <-> R5P (5.3.1.6)

R040: R5P <-> R1P (5.4.2.2)

R041: GLUC + NADP <-> 2kGLUC + NADPH (1.1.1.215)

R042: SUC -> GLC + LEVAN (2.4.1.10)

R043: SUC -> FRU + GLC (3.2.1.26)

R044: ATP + FRU <-> ADP + F6P (2.7.1.4)

R045: ATP + G1P -> ADPGLC + PPI (2.7.7.27)

R046: ADPGLC -> ADP + GLYCOGEN (2.4.1.21)

R047: GLYCOGEN + PI -> G1P (2.4.1.1)

R048: SUC6P -> FRU + G6P (3.2.1.26)

R049: RGT + MTGYX <-> SLGT (4.4.1.5)

R050: SLGT <-> RGT + LAC (3.1.2.6)

R051: PYR + NADH <-> NAD + LAC (1.1.1.28)

R052: PEP + CO2 -> OA + PI (4.1.1.31)

R053: PYR + COA -> ACCOA + FORT (2.3.1.54)

R054: MAL + NAD <-> CO2 + NADH + PYR (1.1.1.38)

R055: ACCOA + ATP + CO2 -> MALCOA + ADP + PI (6.4.1.2)

R056: NDP + PEP -> NTP + PYR (2.7.1.40)

R057: XYL <-> XYLU (5.3.1.5)

R058: ATP + XYLU <-> ADP + X5P (2.7.1.17)

R059: S7P + T3P1 <-> E4P + F6P (2.2.1.2)

R060: ARA <-> RIB (5.3.1.4)

R061: ATP + RIB <-> ADP + LRL5P (2.7.1.16)

R062: LRL5P <-> X5P (5.1.3.4)
