# Supplementary material for: The genome-scale metabolic network analysis of Zymomonas mobilis ZM4 explains physiological features and suggests ethanol and succinic acid production strategies
Source: Microb Cell Fact. 2010 Nov 24;9:94. doi: 10.1186/1475-2859-9-94 (PMC3004842; doi:10.1186/1475-2859-9-94)
Supplement: Additional file 6 — Double gene knockout targets for succinic acid production identified by constraints-based flux analysis [file 1475-2859-9-94-S6.PDF]

Additional file 6. Double gene knockout targets for succinic acid production identified by constraints-based flux analysis

Knockouts	Enzyme	max $v_{biomass}$	
		Biomass (h^{-1})	Succinic acid (mmol/gDCW/h)
Wild type strain		0.35	0
PYR → ACAL + CO_2<br>PYR + NADH ↔ LAC + NAD	pyruvate decarboxylase<br>lactate dehydrogenase	0.15	19.57
ACAL + NADH ↔ ETH + NAD<br>PYR + NADH ↔ LAC + NAD	alcohol dehydrogenase<br>lactate dehydrogenase	0.18	18.8
ACAL + NADH ↔ ETH + NAD<br>FUM ↔ MAL	alcohol dehydrogenase<br>fumarate hydratase	0.35	0.03

The reactions and corresponding enzymes for double gene knockout targets for producing succinic acid are listed. Glucose and oxygen uptake rate are fixed to 11.1 and 0 mmol/gDCW/h, respectively.
