# Supplementary figures and images for: The genome-scale metabolic network analysis of Zymomonas mobilis ZM4 explains physiological features and suggests ethanol and succinic acid production strategies
Source: Microb Cell Fact. 2010 Nov 24;9:94. doi: 10.1186/1475-2859-9-94 (PMC3004842; doi:10.1186/1475-2859-9-94)

Additional file 7. Batch culture profile of *Zymomonas mobilis* ZM4

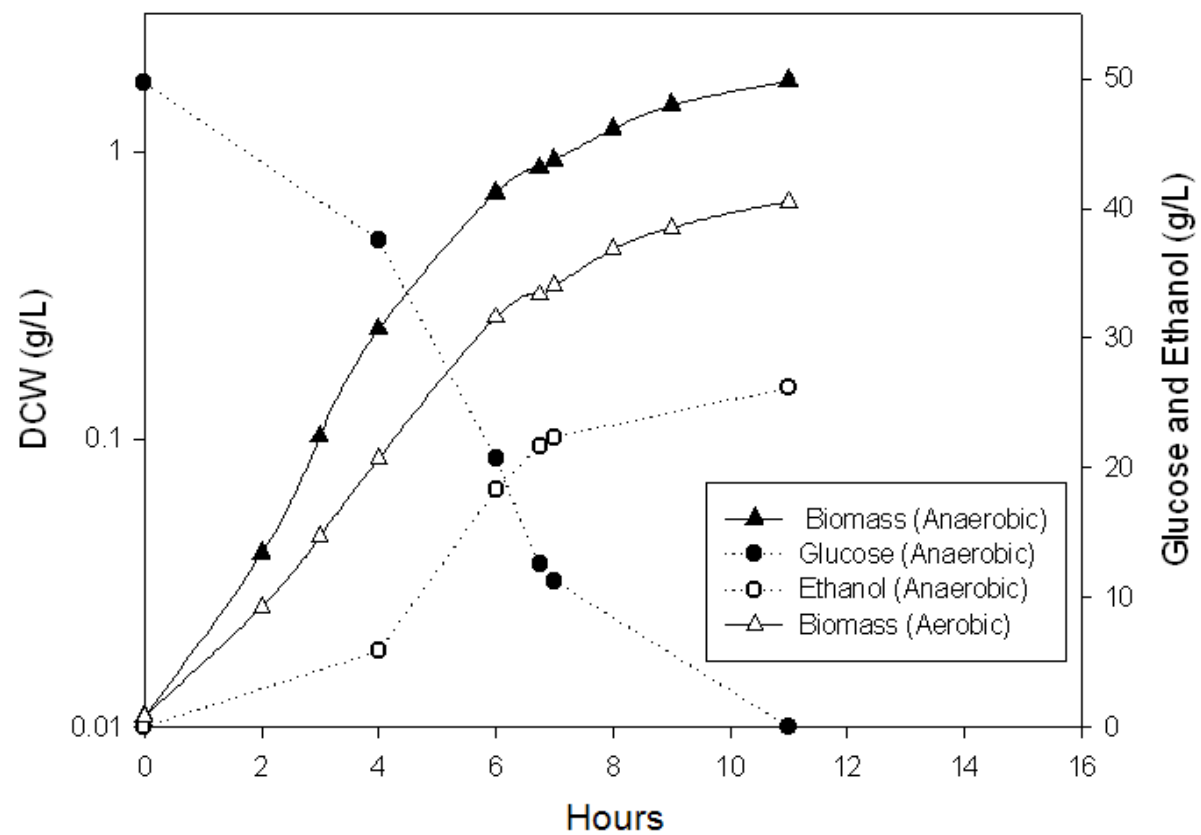

Supplement: Additional file 7 — Batch culture profile of Zymomonas mobilis ZM4 [file 1475-2859-9-94-S7.PDF]
